# Analyses using multiple imputation need to consider missing data in auxiliary variables

**DOI:** 10.1093/aje/kwae306

**Published:** 2024-08-27

**Authors:** Paul Madley-Dowd, Elinor Curnow, Rachael A Hughes, Rosie P Cornish, Kate Tilling, Jon Heron

**Affiliations:** Centre for Academic Mental Health, Population Health Sciences, Bristol Medical School, University of Bristol, Bristol, United Kingdom; MRC Integrative Epidemiology Unit at the University of Bristol, Bristol, United Kingdom; Population Health Sciences, Bristol Medical School, University of Bristol, Bristol, United Kingdom; MRC Integrative Epidemiology Unit at the University of Bristol, Bristol, United Kingdom; Population Health Sciences, Bristol Medical School, University of Bristol, Bristol, United Kingdom; MRC Integrative Epidemiology Unit at the University of Bristol, Bristol, United Kingdom; Population Health Sciences, Bristol Medical School, University of Bristol, Bristol, United Kingdom; MRC Integrative Epidemiology Unit at the University of Bristol, Bristol, United Kingdom; Population Health Sciences, Bristol Medical School, University of Bristol, Bristol, United Kingdom; MRC Integrative Epidemiology Unit at the University of Bristol, Bristol, United Kingdom; Population Health Sciences, Bristol Medical School, University of Bristol, Bristol, United Kingdom; Centre for Academic Mental Health, Population Health Sciences, Bristol Medical School, University of Bristol, Bristol, United Kingdom; MRC Integrative Epidemiology Unit at the University of Bristol, Bristol, United Kingdom; Population Health Sciences, Bristol Medical School, University of Bristol, Bristol, United Kingdom

**Keywords:** ALSPAC, auxiliary variables, bias, missing data, multiple imputation, simulation

## Abstract

Auxiliary variables are used in multiple imputation (MI) to reduce bias and increase efficiency. These variables may often themselves be incomplete. We explored how missing data in auxiliary variables influenced estimates obtained from MI. We implemented a simulation study with 3 different missing data mechanisms for the outcome. We then examined the impact of increasing proportions of missing data and different missingness mechanisms for the auxiliary variable on bias of an unadjusted linear regression coefficient and the fraction of missing information. We illustrate our findings with an applied example in the Avon Longitudinal Study of Parents and Children. We found that where complete records analyses were biased, increasing proportions of missing data in auxiliary variables, under any missing data mechanism, reduced the ability of MI including the auxiliary variable to mitigate this bias. Where there was no bias in the complete records analysis, inclusion of a missing not at random auxiliary variable in MI introduced bias of potentially important magnitude (up to 17% of the effect size in our simulation). Careful consideration of the quantity and nature of missing data in auxiliary variables needs to be made when selecting them for use in MI models.

## Introduction

Missing data can lead to bias in effect estimates and reduced statistical efficiency. Missing data mechanisms for a particular variable include missing completely at random (MCAR), where the probability of an observation being missing is independent of all other variables; missing at random (MAR), where conditional on observed data, the probability of an observation being missing is independent of unobserved data; and missing not at random (MNAR), where the probability of an observation being missing is dependent on unobserved data even after conditioning on observed data.^1^

Strategies for handling missing data include complete records analysis (CRA) and multiple imputation (MI). Complete records analysis analyzes only the subset with complete data for all variables in the analysis model. Multiple imputation creates multiple data sets with imputed values from predictive models (the imputation model), fits the analysis model in each imputed data set, and combines effect estimates using Rubin’s rules.^2^

Recent work has highlighted problems in the traditional categories of missing data types (MCAR, MAR, and MNAR) when data are missing in multiple variables and instead advised understanding when CRA and MI will lead to biased estimates.^3^ In general, unbiased CRA requires missingness to be conditionally independent of the analysis model outcome, given the observed covariate data,^4^ and unbiased MI requires that each partially observed variable is conditionally unrelated to missingness, given the observed data for its imputation model predictors (and assuming all imputation models are correctly specified and compatible with the analysis model).^5^

In MI, auxiliary variables are included in the imputation model but not the analysis model. They can reduce bias, compared to CRA, if they predict missingness in a variable and predict missing values, or improve the precision of effect estimates if they predict the missing values only.^6^^‑^^9^ For example, consider a study assessing the effect of maternal smoking during pregnancy (the exposure) on offspring intelligence quotient (IQ) scores (the outcome), adjusted for confounders. Intelligence quotient scores may be more likely to be missing for individuals with an intellectual disability, whose IQ scores would be lower on average than the general population. Such a scenario would lead to biased CRA estimates for the effect of maternal smoking during pregnancy on offspring IQ as missingness is dependent on the outcome.^4^ Including an auxiliary variable predictive of the missing outcome values, such as educational attainment obtained via linked educational records, in the imputation model (which would be expected to be positively correlated with IQ) would reduce the dependency between the partially observed outcome variable and missingness, thereby reducing bias in MI estimates.^10^ A directed acyclic graph (DAG)^11^ of this example is provided in Figure S1 (Appendix S1).

Historically, it was recommended to use as many auxiliary variables in the imputation model as possible to “reduce the chance of omitting an important cause of missingness,” reduce bias, and improve efficiency with minimal cost.^8^ This view has been challenged in its extreme by work showing that model performance degraded (in terms of bias and precision) as the number of included auxiliary variables approached the number of records with complete data.^9^ The inclusion of auxiliary variables can induce or exacerbate bias, for example, where the auxiliary variable is a collider and conditioning upon it would induce a dependency between a partially observed variable and missingness in that variable, thereby violating the MAR assumption,^12^^‑^^14^ or where data are MNAR and the auxiliary variable is strongly predictive of only missingness.^15^ Consideration of the causal relationships between each variable in the analysis model and the missingness mechanism is therefore essential when deciding which auxiliary variables should be included in imputation models.

Potential auxiliary variables may be incomplete. The amount of incomplete auxiliary data will not affect a CRA because auxiliary variables are not in the analysis model. However, completeness of auxiliary variables could affect (1) the strength of the conditional dependence between the partially observed variable and missingness (and therefore bias), and (2) the quantity of random noise introduced by imputing missing values of auxiliary variables. Prior work has briefly explored the impact of missing data in an auxiliary variable but has only investigated a single proportion of missing data (20%) and did not explore the impact of different missing data mechanisms.^10^ Further research is needed to investigate the impact of both (1) increasing proportions of missing auxiliary data and (2) different missing data mechanisms for auxiliary variables on bias and efficiency of estimates obtained from MI.

## Methods

As an applied example, we investigated the relationship between maternal smoking during pregnancy and offspring IQ at age 15 years in the Avon Longitudinal Study of Parents and Children (ALSPAC). The outcome (offspring IQ) was incomplete, and 2 auxiliary variables were available which are not in the main analysis model but are correlated with the outcome. These were IQ at age 8 and a continuous attainment score for secondary education. The former contained more missing data but had a higher correlation with the outcome than the latter. We wished to improve our decision-making about which auxiliary variables to include in the imputation models when the auxiliary variables contain missing data.

To aid our understanding, we undertook a simulation study that expands on the applied example to show how different missing data mechanisms for the outcome and auxiliary variables, with increasing proportions of missing data in auxiliary variables, may influence results obtained using MI. All analyses were conducted using Stata version 17.

### Applied example

Data were taken from ALSPAC^16^^‑^^18^ which recruited 14 541 pregnant women resident in Avon, United Kingdom, with expected dates of delivery April 1, 1991, to December 31, 1992. Of these pregnancies, 13 988 children were alive at 1 year of age. Inclusion criteria were being from a singleton pregnancy and surviving to 1 year of age, leaving a sample size of 13 826.

The substantive analysis was a linear regression of offspring IQ at age 15 on maternal smoking in pregnancy adjusted for the confounders maternal age, education, parity, and offspring sex. Maternal smoking during pregnancy was a self-reported binary variable collected at 18 weeks’ gestation, and offspring IQ was measured using the Wechsler Abbreviated Scale of Intelligence at age 15 years.^19^ The auxiliary variables used in the imputation models were IQ at age 8, measured using the Wechsler Intelligence Scale for Children—III^20^ and the capped Key Stage 4 (KS4) point score, a measure of educational attainment typically achieved at age 16 obtained from linkage to the National Pupil Database in 2011 and equal to the total score of an individual’s top 8 GCSE or equivalent qualifications ranked in terms of points. The DAG in Figure S1 of Appendix S1 shows the assumed relationships between variables and indicators for missing data; justification for these assumptions is also provided. To simplify this example, we excluded participants if they had missing data for any of the confounders. Participants with missing data in auxiliary variables were retained. The exposure was completely observed once participants with missing confounder information were excluded. Following exclusions, our sample size was 11 780.

We performed fully conditional specification (FCS) MI^21^ using Stata’s *mi impute chained* command with 1000 imputations and 25 burn-in iterations; estimates in each imputed data set were combined using Rubin’s rules.^2^ Linear regression was used as the imputation model for all variables with missing data. Six imputation models were investigated: (i) excluding all auxiliary variables, (ii) including IQ at age 8, (iii) including KS4 score, (iv) including IQ at age 8 and KS4 score, (v) including KS4 score cubed and a multiplicative term for KS4 score cubed and maternal education and (vi) including both IQ at age 8 and KS4 score cubed with a multiplicative term between KS4 score cubed and maternal education. In model v and vi, KS4 score was imputed using the cube roots of IQ and a multiplicative term with maternal education. Models v and vi were included to highlight the importance of correctly specifying the imputation model, as previous work has shown that IQ and KS4 attainment were related early and the relationship varied according to maternal education.^22^

We report the effect estimate and standard error of the exposure coefficient (ie, the effect of maternal smoking during pregnancy on offspring IQ at age 15) and the fraction of missing information (FMI). The FMI is a parameter-specific measure that quantifies the loss of information due to missing data while accounting for information recovered by MI.^2^^,^^23^ Values of FMI range between 0 and 1. Values close to 1 indicate high variability between imputed data sets, meaning that observed data in the imputation model does not provide much information about the missing data. Further detail on the FMI is provided in Appendix S2. A large number of imputations was used as the estimate of the FMI is highly variable.^24^

### Simulation study

#### Data generation model

Table 1 summarizes all simulation design factors and levels investigated. We generated 1000 independent simulated data sets of sample size 1000. Each data set consisted of continuous variables *Y* (the outcome), *X* (the exposure), and *Z* (an auxiliary variable correlated with *Y* but not *X*), simulated from a multivariate normal distribution. Each variable had nonzero mean ($\overline{Y\ }$= 6, $\overline{X\ }$= −3, $\overline{Z}$ = 2) and variance 1. The correlation of *Y* and *X* was held constant at 0.6, while the correlation of *Z* and *Y* was varied between 0.1 and 0.7 in increments of 0.2. We provide justification for our selected number of simulations, sample size, and variable distributions in Appendix S3.

**Table 1:** Simulation design factors and chosen levels. The simulation study was conducted under every combination of values.

**Factor**	**Values implemented**
% Missing data in the outcome *(Y)*	50%
Missing data mechanism for outcome	Mechanism 1) missingness in *Y* (*M_Y_*) caused by the exposure *(X)* and auxiliary variable *(Z)*
	Mechanism 2) missingness in *Y* caused by the outcome variable *(Y)* Mechanism 3) missingness in *Y* caused by the exposure variable only
% Missing data in the auxiliary variable	0%-90% in increments of 10%
Missing data mechanism for auxiliary variable	Mechanism 1) missingness in *Z* (*M_Z_*) not caused by any other variables
	Mechanism 2) missingness in *Z* caused by the auxiliary variable only
	Mechanism 3) missingness in *Z* caused by *W* only, where *W* was derived as a completely observed standardized normal variable caused by *Z* (random draw from standardized normal distribution +0.6 × *Z*, then standardized)
Correlation between exposure and outcome	0.6
Correlation between outcome and auxiliary	0.1, 0.3, 0.5, 0.7
Correlation between exposure and auxiliary	0
Models estimated	1) Complete records analysis
	2) Multiple imputation excluding auxiliaries—Imputation model for *Y*: *p(Y|X)*
	3) Multiple imputation with *Z* as an auxiliary for *Y—*Imputation model for *Y*: *p(Y|X, Z)* Imputation model for *Z*: for missing auxiliary mechanism 1 and 2—*p(Z|Y, X)* for missing auxiliary mechanism 3—*p(Z|Y, X,W)*

**Figure 1:**
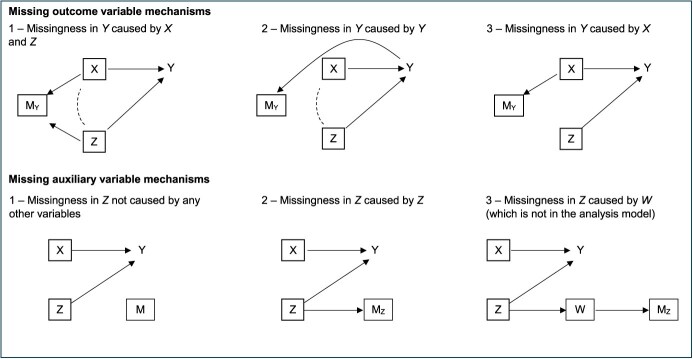
Directed acyclic graphs of the missing data mechanisms used for the outcome Y and auxiliary variable Z. Dashed arrows represent induced correlations that do not exist in the complete data. In outcome missingness mechanism 1, complete records analysis of a regression of Y on X is biased due to conditioning on the collider M_Y_ which induces correlation between X and Z. In outcome missingness mechanism 2, bias in complete records analysis occurs in the same way following conditioning on a child variable of the outcome Y which is a collider for X and Z. In outcome missingness mechanism 3, complete records analysis is unbiased. The missing data mechanisms for the outcome and auxiliary variables are presented separately for simplicity but can be combined to reflect the missing data in both variables.

We simulated 50% missing data in the outcome under 3 different mechanisms, displayed in Figure 1 as DAGs. The missing outcome mechanisms were as follows:

- Outcome mechanism (1) outcome missingness (*M_Y_*) was caused by the exposure *(X)* and auxiliary variable *(Z)*; it was therefore independent of the outcome *(Y)* given complete exposure and auxiliary.
- Outcome mechanism (2) outcome missingness was caused by the outcome itself.
- Outcome mechanism (3) outcome missingness was caused by the exposure variable only; it was therefore independent of the outcome variable given complete exposure.

In missing outcome mechanism 1, *Y* was set to missing if the cumulative distribution function (CDF) of *X* and *Z* was less than $\sqrt{0.5}$. In missing outcome mechanism 2, *Y* was set to missing if the CDF of *Y* was less than 0.5. This mechanism was chosen to reflect the scenario where data in IQ are likely to be missing dependent on intelligence, and we are using a proxy outcome such as educational attainment score to reduce the dependence of the outcome missingness on the outcome itself. In missing outcome mechanism 3, *Y* was set to missing if the CDF of *X* was less than 0.5. In outcome mechanisms 1 and 2, CRA is biased and MI using *Z* as an auxiliary would be implemented to reduce bias and improve efficiency. For outcome mechanism 3, CRA is unbiased, and MI using *Z* would be implemented for the purposes of improving efficiency only.

Missing data in the auxiliary variable was varied between 0 and 90%, simulated using 3 different missing data mechanisms (see Figure 1):

- Auxiliary mechanism (1) auxiliary missingness (*M_Z_*) was not caused by any other variable.
- Auxiliary mechanism (2) auxiliary missingness caused by the auxiliary itself.
- Auxiliary mechanism (3) auxiliary missingness caused by another variable, *W*, associated with the auxiliary; auxiliary missingness is therefore independent of the outcome and the auxiliary given complete *W.*

In missing auxiliary mechanism 1, *Z* was set to missing if a random draw from the uniform distribution bounded by 0 and 1 was less than *μ*, where *μ* was varied between 0 and 0.9 in increments of 0.1. In missing auxiliary mechanism 2, *Z* was set to missing if the CDF of *Z* was less than *μ*. In missing auxiliary mechanism 3, *W* was equal to 0.6 times *Z* plus a draw from a standard normal distribution (and then standardized), and *Z* was set to missing if the CDF of *W* was less than *μ*. Auxiliary mechanism 3 was included to explore the impact of making the auxiliary missingness independent of the partially observed outcome and auxiliary variables given another variable not included in the analysis model (akin to an auxiliary for the auxiliary). Ignoring missing data in other variables (here only the outcome is also missing), under missing auxiliary mechanism 1, the auxiliary variable is MCAR; under mechanism 2, the auxiliary is MNAR; and under mechanism 3, the auxiliary is MAR given *W*.

### Analysis and imputation models

The analysis model consisted of a linear regression of *Y* on *X.* The true value of the effect estimate for *X* was equal to 0.6. For each scenario, the effect of *X* on *Y* was estimated using (i) CRA and (ii) MI where *Z* was not included as an auxiliary variable–missing *Y* imputed under the conditional model p(Y|X). We further estimated the effect of *X* on *Y* using (iii) MI analysis where *Z* was included as an auxiliary variable for *Y* – missing *Y* imputed under the conditional model *p(Y|X,Z)*. For missing auxiliary mechanism 1 and 2, *Z* was imputed using the conditional model *p(Z|Y,X)*. For missing auxiliary mechanism 3, where *W* acted as a proxy for *Z* (but not *Y)*, *Z* was imputed using the conditional model *p(Z|Y, X, W)*. The FCS MI was performed using Stata’s *mi impute chained* command using 100 imputations and 10 burn-in iterations and estimates in each imputed data set were combined using Rubin’s rules.^2^ Linear regression was used as the imputation model for all variables.

### Simulations and metrics

We report the bias, standard error (SE), and FMI (for MI models) of the effect estimate of *X* on *Y* for each scenario with Monte Carlo SEs. Mean bias and SE were calculated using the *simsum* command in Stata^25^ and FMI for the exposure coefficient using *mi estimate*.

### Sensitivity analyses

Sensitivity analyses explored the impact of (1) the proportion of missing data in the outcome and (2) the strength of association between *X* and *Y* relative to *Z* and *Y.* We further explored the impact of omitting *W* from the imputation model under missing auxiliary mechanism 3. These analyses and their results are detailed in Appendix S3.

## Results

### Applied example

Supplementary Table S1 shows descriptive statistics for the sample. Data in IQ at age 15 (outcome) were missing for 60% of the sample, in IQ at age 8 (auxiliary) for 44.2%, and in KS4 scores (auxiliary) for 25.1%. The observed correlation between IQ at age 8 and 15 was 0.63, and between KS4 score and IQ age 15 was 0.59. Maternal smokers during pregnancy were more likely to have higher parity, lower levels of education, and slightly more likely to have male offspring.

Table 2 shows results for our applied example. We discuss the effect estimates here and the FMI in Appendix S1. In CRA, smoking during pregnancy was associated with a 0.87-point reduction (95% CI, −1.82 to 0.08) in offspring IQ score at age 15. Comparable effect estimates were found between CRA and MI excluding auxiliary variables (MI model i). Using IQ at age 8 as an auxiliary in MI model ii resulted in a greater effect size of a 1.13-point reduction (95% CI, −2.03 to −0.23). Use of KS4 score as an auxiliary (model iii) further increased the effect size to a 1.99-point reduction (95% CI, −2.87 to −1.10). Using both KS4 score and IQ at age 8 (model iv) provided a similar effect size and standard error to use of KS4 only. MI with KS4 score including nonlinear terms (model v) attenuated the effect estimate to a 1.40-point reduction (95% CI, −2.29 to −0.52). Including IQ at age 8 and KS4 score with nonlinear terms (model vi) resulted in a slightly higher estimate of a 1.49-point reduction (95% CI, −2.31 to −0.68) with the lowest standard error of all models.

**Table 2:** Model results for applied MI analyses.

**Model**	**Exposure coefficient (s.e.)** ^**a**^	**95% CI**	**FMI for exposure coefficient**
CRA	−0.87 (0.485)	−1.82 to 0.08	
i) MI excluding auxiliaries	−0.86 (0.485)	−1.81 to 0.09	0.70
ii) MI with IQ at age 8 as an auxiliary	−1.13 (0.459)	−2.03 to −0.23	0.66
iii) MI with KS4 score as an auxiliary	−1.99 (0.453)	−2.87 to −1.10	0.61
iv) MI with IQ at age 8 and KS4 score as auxiliaries	−1.93 (0.444)	−2.80 to −1.06	0.61
v) MI with KS4 score cubed as an auxiliary (including multiplicative term^b^)	−1.40 (0.452)	−2.29 to −0.52	0.64
vi) MI with IQ at age 8 and KS4 score cubed as auxiliaries (including multiplicative term^b^)	−1.49 (0.414)	−2.31 to −0.68	0.58

^a^Mean difference in IQ at age 15 among offspring of maternal smokers during pregnancy compared to nonsmokers during pregnancy.

^b^Multiplicative term between KS4 score cubed and maternal education.

### Simulation study

Supplementary Table S2 and Table S3 present the bias, SE, and Monte Carlo SE for CRA models, and additionally the FMI for MI models. As expected, CRA was biased under missing outcome mechanisms 1 and 2 and unbiased under mechanism 3. As the correlation between *Y* and *Z* increased, the bias in CRA increased for outcome mechanism 1 but remained constant for outcome mechanism 2. Models excluding auxiliaries were biased to a similar extent as CRA under mechanism 1 and 2, and both were unbiased under mechanism 3.

**Figure 2:**
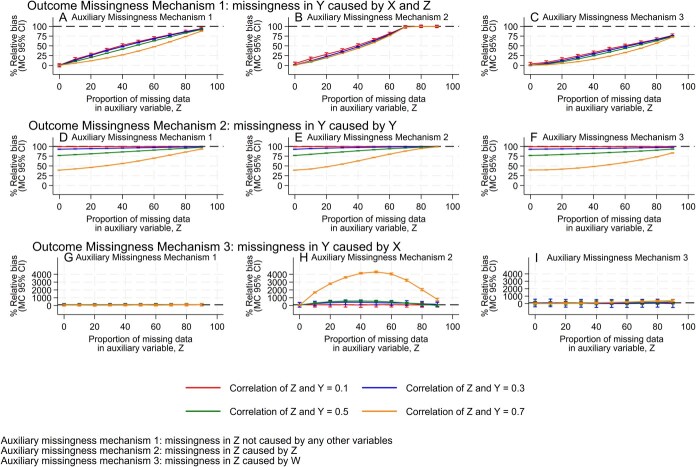
Plots of bias of the effect estimate of the exposure, X, across simulations against the proportion of missing data in the auxiliary variable, Z, for each level of correlation between the auxiliary, and outcome, Y. Bias is presented as relative to CRA. Solid lines correspond to bias in MI analysis including auxiliary variables, while dashed lines correspond to bias in CRA, which was approximately equivalent to MI excluding auxiliary variables. The quantity of bias in CRA and MI models is presented in Supplementary Table S2 and S3. Larger plots can be viewed in Appendix S4. Abbreviations: CI , confidence interval; MC, Monte Carlo.

Results for patterns of missing data in *Y* and Z, the SE and FMI are presented in Appendix S3, Figures S2-S4. We display plots of bias relative to CRA for all outcome and auxiliary missing data mechanism combinations in Figure 2. For missing outcome mechanism 1 (outcome missingness, *M_Y_*, caused by exposure and auxiliary; Figure 2A-C), where there was no missing data in the auxiliary variable, MI models including auxiliaries resulted in no bias under all auxiliary missing data mechanisms. As the proportion of missing auxiliary data increased, bias in MI models approached that of CRA. For a given proportion of missing auxiliary data, bias was closer to that of CRA for auxiliary variables with weaker correlation with the outcome than those with stronger correlation. Bias relative to CRA was greater when the auxiliary missingness, *M_Z_*, did not depend on any other variable (Plot A) than when it depended on another variable (*W*) not included in the analysis model (Plot C). This was true at each proportion of missing data, with bias in the latter case remaining below 80% of CRA even at 90% missing data in the auxiliary. When the auxiliary missingness depended on the auxiliary variable itself (Plot B), bias reached that of CRA by 70% missing auxiliary data.

Under missing outcome mechanism 2 (outcome missingness caused by its own value, where the auxiliary acts as a proxy; Figure 2D-F), MI including auxiliary variables never completely removed bias. Bias reduction was greater when the auxiliary variable strongly predicted the missing outcome—the weakest correlation (0.1) did not result in any bias reduction. Bias approached that of CRA as the missing data in the auxiliary variable increased. The missing data mechanism for the auxiliary variable did not have a large influence on the magnitude of bias reduction.

Under missing outcome mechanism 3 (outcome missingness caused by the exposure only; Figure 2G-I), no bias was observed when the auxiliary missingness data did not depend on any other variable (Plot G). When the auxiliary missingness was caused by the auxiliary itself (Plot H), bias was observed in MI models that included auxiliary variables. Bias was larger when the auxiliary had a stronger correlation with the outcome and increased as the proportion of missing auxiliary data increased from 0% to 50% but then declined as the proportion rose from 50% to 90%. At its greatest, bias in MI was over 4000% that of CRA (bias in CRA = −0.002) when the correlation between *Y* and *Z* was 0.7 and there was 50% missing data in the auxiliary. In terms of absolute bias in the MI estimate, this equates to around 0.1 units, or 17% of the true effect size. When auxiliary missingness was independent of the auxiliary variable itself given *W* (Plot I), bias was low for all proportions of missing auxiliary data and correlations between outcome and auxiliary.

The sensitivity analyses are reported in Appendix S3. Briefly, the observed patterns of bias did not change substantially through the introduction of more missing data in the outcome or by reducing the correlation between *X* and *Y.* Omission of *W* from the imputation model under missing auxiliary mechanism 3 led to smaller reductions in bias under missing outcome mechanisms 1 and 2 and an introduction of bias under outcome mechanism 3 that was similar to that seen in Figure 2(H).

## Discussion

In this simulation study, we demonstrated that increasing proportions of missing data in auxiliary variables limit their ability to reduce bias and recover information lost to missing data (as measured by the FMI) when included in imputation models. When MI is used for the purposes of improving efficiency only (ie, there is no bias in CRA), including an auxiliary variable with missing data dependent on its own value within an imputation model can induce bias. This bias was greatest when using an auxiliary that was a strong predictor of the outcome. Guidance on MI advises that such an auxiliary variable is desirable.^7^ We highlight the importance of considering the quantity and causes of missing data in such auxiliaries.

Based on the DAG we propose for the applied example (Appendix S1), IQ at age 15 was likely missing according to outcome missingness mechanism 2 in the simulation study (an outcome missing dependent on its own value). The CRA is therefore likely to be biased^4^; however, if the assumptions of our DAG are correct, using MI with auxiliary variables that act as proxies for the missing outcome can reduce this bias.^10^^,^^22^ We used 2 auxiliary variables in MI models, each with auxiliary missingness mechanism 2 in the simulation study (the auxiliary variables are missing dependent on their own value).

The true effect of maternal smoking in pregnancy on offspring IQ is unknown, as are the quantity of bias in CRA and which MI model achieved the greatest reduction in bias. If an analyst had to choose between the 2 auxiliaries, we would suggest the use of KS4 score over IQ at age 8, as they have similar observed correlation with the outcome variable, but KS4 is more complete. It is important to consider the nature of the relationship between an auxiliary variable and the variable being imputed (ie, correctly specifying the imputation model).^5^ Not accounting for the nonlinear relationship between KS4 score and IQ at age 15 in the imputation model (as in models 3 and 4) possibly led to overestimation of the effect.

In our example, we are not forced to choose between the 2 auxiliary variables and so consider model 6, which contained both, to provide the best effect estimate. Our reasons are that this model (1) captures the nonlinear relationship between the auxiliary and the outcome and (2) contains 2 auxiliary variables that are missing for different groups of people (see Appendix S1 for further detail), meaning that proxy information is available for a wider coverage of the distribution of missing outcome values. This highlights the need to consider the missing data mechanisms of the auxiliary variable and the variable to be imputed in context of one another.

In the applied example, the outcome and IQ age 8 were likely to be missing for similar groups of people, while the outcome and KS4 score were likely missing for 2 different groups. IQ (at age 8 and 15) was more likely to be missing for those with lower IQ (due to the socioeconomic patterning of participation in ALSPAC), whereas KS4 score was more likely to be missing for those attending an independent school (who were more likely to have higher IQ and KS4 scores). We cannot know if the difference between effect estimates obtained using imputation models including IQ at age 8 (model ii) vs KS4 score (including nonlinear terms; model v) is due to using an auxiliary with more missing data or the auxiliary having a more similar missing data mechanism to the outcome (both occurred in model ii). This was not explored in the simulation study but warrants further investigation as overlap in missing data between variables may affect the ability of the auxiliary to predict missing values of the incomplete variable. At the extreme, if the auxiliary is either (1) always missing when *Y* is missing or (2) always missing when *Y* is observed, then the auxiliary will be no use whatsoever.

In the simulation study, auxiliary and outcome missingness were simulated under similar mechanisms; lower values of the outcome and auxiliary were both more likely to be missing. Therefore, missing data in the outcome and auxiliary simultaneously is more likely in the simulation study and model ii of the applied example, but less likely in model v. Despite this, bias was still reduced by the inclusion of the incomplete auxiliary, *Z*, in the imputation model, but further investigation is needed to see if a greater reduction may have occurred with smaller overlap in missing data between the outcome and auxiliary.

### Limitations

Our simulation scenarios were simple, using linear regression to relate continuous variables. The influence of missing auxiliary data on bias and efficiency in models with interaction terms or logistic regression models may differ, for example, because of noncollapsibility of the effect estimate.

We used a deterministic method to induce missing data in the outcome and auxiliary variables (ie, an exact cut off for the CDF was used) as opposed to a stochastic process. We also used only a single mechanism for missing data in each variable in our simulations. More complex missing data mechanisms, including multiple reasons for missing data, may be more realistic. Bias in real-world studies may therefore be greater or lesser than in our simulation study, dependent on the nature of the missing data mechanisms.

Missing data in analysis model variables was only assessed for an incomplete outcome, and not for incomplete exposure or confounding variables. Bias may be different when missing data occur in such variables (compare results for ${\mathrm{\beta}}_{XY}$ to ${\mathrm{\beta}}_{YX}$ in the study by Collins et al.^8^), potentially as a result of Berkson error.^26^

Finally, we only used a single auxiliary variable in our simulation study. In practice, several auxiliary variables may be used, each of which may be incomplete. In this case, the missing data for each auxiliary variable need to be considered.

## Conclusions

Careful consideration is required for the use of auxiliary variables in multiple imputation models. We suggest the following guidance:

Use auxiliary variables that are completely observed, or have smaller amounts of missing data, in preference to those which are similarly correlated with the incomplete variable but are less complete themselves. It should be noted, however, that except in cases where CRA was unbiased, including an incomplete auxiliary variable that was strongly predictive of the incomplete analysis model variable in MI was seemingly no worse (in terms of bias and efficiency) than CRA or an analysis excluding auxiliary variables from MI.

Explore the missing data mechanisms of incomplete auxiliary variables in addition to incomplete analysis model variables.

Where auxiliary variables are incomplete, aim to use auxiliary variables that are most predictive of the partially observed variables and independent of their own missingness, conditional on the other predictors in the imputation model.

## Supplementary Material

Web_Material_kwae306

## Data Availability

The ALSPAC data used in the applied example cannot be shared publicly for ethical reasons. The study website contains details of all available data through a fully searchable data dictionary (http://www.bristol.ac.uk/alspac/researchers/our-data/). The scripts and folder structure used to run the applied example analysis and simulation study can be found online at https://github.com/pmadleydowd/Missing_auxiliary_variables. Data sets for the simulation study are found within this repository.
