# Supplementary material for: Analyses using multiple imputation need to consider missing data in auxiliary variables
Source: Am J Epidemiol. 2024 Aug 27;194(6):1756–63. doi: 10.1093/aje/kwae306 (PMC12133271; doi:10.1093/aje/kwae306)
Supplement: Web_Material_kwae306 [file web_material_kwae306.zip › Accepted_TECHNICAL_REVIEW_AJE-Supplementary materials.docx]

Supplementary materials

Authors: Paul Madley-Dowd, Elinor Curnow, Rachael A. Hughes, Rosie Cornish, Kate Tilling, Jon Heron

Contents

[**Supplementary tables referred to in manuscript** 4](#_Toc171514832)

[**Table S1**: Descriptive statistics for ALSPAC sample meeting inclusion criteria in the applied example. 4](#_Toc171514833)

[**Table S2**: Average bias, standard error, FMII (multiple imputation only) across simulations for complete records analysis and multiple imputation models excluding auxiliary variables. 5](#_Toc171514834)

[**Table S3**: Average bias, standard error and fraction of missing information (FMI) across simulations for multiple imputation models including auxiliary variables. 7](#_Toc171514835)

[**Appendix S1 - Applied example** 17](#_Toc171514836)

[Assessment of the assumed relationship between variables and missing data in the applied example 17](#_Toc171514837)

[**Figure S1**: Directed acyclic graph for the applied example. Lines in blue show relationships between variables that are part of the complete records analysis model. 17](#_Toc171514838)

[Covariates included in the analysis model 17](#_Toc171514839)

[Auxiliary variables 18](#_Toc171514840)

[Patterns of missing data and missing data mechanisms 19](#_Toc171514841)

[**Table S4**: Patterns of missing data in the outcome (IQ at age 15) and auxiliary variables (IQ at age 8 and KS4 score recorded at age 16). 21](#_Toc171514842)

[**Table S5**: Odds ratios for missing data in the outcome and auxiliary variables according to the variables included in the applied example. 21](#_Toc171514843)

[Fraction of missing information (FMI) results 22](#_Toc171514844)

[Sensitivity analyses 22](#_Toc171514845)

[Distribution of missing data according to included vs. excluded participants 22](#_Toc171514846)

[**Table S6**: Distribution of observed and missing data in the sample with all confounders observed vs the sample with some missing confounder information. 22](#_Toc171514847)

[Reducing the number of imputations used in the applied example 23](#_Toc171514848)

[**Table S7**: Model results for applied MI analyses using 100 imputations. 23](#_Toc171514849)

[**Appendix S2 - Further details on the fraction of missing information (FMI)** 24](#_Toc171514850)

[**Appendix S3 - Simulation study** 25](#_Toc171514851)

[Justification for the number of simulation repetitions, sample size and distribution of simulated variables 25](#_Toc171514852)

[Pattens of missing data 25](#_Toc171514853)

[Standard error (SE) results 26](#_Toc171514854)

[Fraction of missing information (FMI) results 27](#_Toc171514855)

[**Figure S2**: Plots of the **mean proportion with each missing data pattern** (Y and Z missing, Y only missing, Z only missing) across simulations against the proportion of missing data in the auxiliary variable, Z, for each level of correlation between the auxiliary, and outcome, Y. 28](#_Toc171514856)

[**Figure S3**: Plots of **mean SE** of the effect estimate of the exposure, X, across simulations against the proportion of missing data in the auxiliary variable, Z, for each level of correlation between the auxiliary, and outcome, Y. 29](#_Toc171514857)

[**Figure S4**: Plots of **mean FMI** of the effect estimate of the exposure, X, across simulations against the proportion of missing data in the auxiliary variable, Z, for each level of correlation between the auxiliary, and outcome, Y. 30](#_Toc171514858)

[Sensitivity analyses 31](#_Toc171514859)

[Methods 31](#_Toc171514860)

[Results 31](#_Toc171514861)

[Bias for sensitivity analysis i) and ii) 31](#_Toc171514862)

[FMI for sensitivity analyses i) and ii) 32](#_Toc171514863)

[**Table S8**: Sensitivity analysis – average bias, standard error and fraction of missing information across simulations in complete records analysis and MI models excluding auxiliary variables for sensitivity analysis i) and ii). 34](#_Toc171514864)

[**Table S9**: Sensitivity analysis – average bias, standard error and fraction of missing information across simulations in MI models including auxiliary variables for sensitivity analysis i) and ii). 35](#_Toc171514865)

[**Figure S5**: Plots of bias relative to CCA for the exposure (X) coefficient against the proportion of missing data in Z for sensitivity analysis that i) increased the missing data in the outcome to 75% (plots A-C) and ii) decreased the correlation between X and Y to 0.2 (plots D-E). 42](#_Toc171514866)

[**Figure S6**: Plots of the FMI for the exposure (X) coefficient against the proportion of missing data in Z for sensitivity analysis that i) increased the missing data in the outcome to 75% (plots A-C) and ii) decreased the correlation between X and Y to 0.2 (plots D-E). 43](#_Toc171514867)

[Bias and FMI when excluding W from the imputation model under missing auxiliary mechanism 3 44](#_Toc171514868)

[**Table S10**: Sensitivity analysis - average bias, standard error and fraction of missing information across simulations for the exposure (X) coefficient for multiple imputation models that excluded or included W in the imputation model under missing auxiliary mechanism 3 45](#_Toc171514869)

[**Figure S7**: Plots of bias (relative to CCA for outcome mechanisms 1 and 2, and absolute for outcome mechanism 3) for the exposure (X) coefficient against the proportion of missing data in Z for sensitivity analysis that excluded W from the imputation model under missing auxiliary mechanism 3 (plots A-C). 49](#_Toc171514870)

[**Figure S8**: Plots of the FMI for the exposure (X) coefficient against the proportion of missing data in Z for sensitivity analysis that excluded W from the imputation model under missing auxiliary mechanism 3 (plots A-C). 50](#_Toc171514871)

[**Appendix S4 - Individual plots for simulation study Figures 2, S3 and S4 by outcome missingness mechanism** 51](#_Toc171514872)

[Bias – Figure 2 51](#_Toc171514873)

[SE – Figure S3 54](#_Toc171514874)

[FMI – Figure S4 57](#_Toc171514875)

[References 60](#_Toc171514876)

# **Supplementary tables referred to in manuscript**

## **Table S1**: Descriptive statistics for ALSPAC sample meeting inclusion criteria in the applied example.

|  | Total | Non-smoker at 18 weeks' gestation | Current smoker at 18 weeks' gestation | Total with complete data in variable, N(%) | Non-smoker at 18 weeks' gestation with complete data in variable, N(%) | Current smoker at 18 weeks' gestation with complete data in variable, N(%) |
| --- | --- | --- | --- | --- | --- | --- |
|  | N=11,780 | N=8,812 | N=2,968 | N=11,780 | N=8,812 | N=2,968 |
| Offspring IQ at age 15 ^a^ | 94.6 (13.0) | 95.1 (13.0) | 92.0 (12.7) | 4,714 (40.0%) | 3,932 (44.6%) | 782 (26.3%) |
| Offspring IQ at age 8 ^a^ | 104.5 (16.4) | 105.2 (16.4) | 101.2 (16.0) | 6,567 (55.8%) | 5,328 (60.45) | 1,239 (41.7%) |
| Offspring KS4 score ^a^ | 325.2 (90.5) | 337.0 (84.1) | 289.4 (99.4) | 8,826 (74.9%) | 6,646 (75.4%) | 2,180 (75.5%) |
| Maternal age at birth ^a^ | 28.3 (4.9) | 28.9 (4.6) | 26.6 (5.1) | 11,780 (100%) | 8,812 (100%) | 2,968 (100%) |
| Parity Categories ^b^ |  |  |  | 11,780 (100%) | 8,812 (100%) | 2,968 (100%) |
| 0 | 5,295 (44.9%) | 3,965 (45.0%) | 1,330 (44.8%) |  |  |  |
| 1 | 4,166 (35.4%) | 3,219 (36.5%) | 947 (31.9%) |  |  |  |
| 2+ | 2,319 (19.7%) | 1,628 (18.5%) | 691 (23.3%) |  |  |  |
| Maternal highest education ^b^ |  |  |  | 11,780 (100%) | 8,812 (100%) | 2,968 (100%) |
| O-level or lower | 7567 (64.2%) | 5201 (59.0%) | 2366 (79.7%) |  |  |  |
| A-level/Degree or higher | 4,213 (35.8%) | 3,611 (41.0%) | 602 (20.3%) |  |  |  |
| Offspring assigned sex at birth ^b^ |  |  |  | 11,780 (100%) | 8,812 (100%) | 2,968 (100%) |
| Female | 5,714 (48.5%) | 4,334 (49.2%) | 1,380 (46.5%) |  |  |  |
| Male | 6,066 (51.5%) | 4,478 (50.8%) | 1,588 (53.5%) |  |  |  |

^a^ – mean (SD) for second to fourth columns
^b^ – N(%) for second to fourth columns

## **Table S2**: Average bias, standard error, FMII (multiple imputation only) across simulations for complete records analysis and multiple imputation models excluding auxiliary variables.

CRA = Complete Records Analysis; MCSE = Monte Carlo Standard Error

|  |  |  | CRA model | | MI model **excluding** auxiliary variable Z | | |
| --- | --- | --- | --- | --- | --- | --- | --- |
| Missing outcome mechanism | Missing auxiliary mechanism | Correlation between Z and Y | Bias (MCSE) | Standard error (MCSE) | Bias (MCSE) | Standard error (MCSE) | FMI (MCSE) |
| Mechanism 1 | Mechanism 1 | 0.1 | -0.047 (0.0008) | 0.0354 (0.00006) | -0.046 (0.0008) | 0.0354 (0.00007) | 0.486 (0.0015) |
|  |  | 0.3 | -0.139 (0.0008) | 0.0349 (0.00005) | -0.139 (0.0008) | 0.0350 (0.00007) | 0.490 (0.0014) |
|  |  | 0.5 | -0.231 (0.0008) | 0.0341 (0.00005) | -0.231 (0.0008) | 0.0342 (0.00007) | 0.489 (0.0015) |
|  |  | 0.7 | -0.323 (0.0008) | 0.0327 (0.00005) | -0.323 (0.0008) | 0.0329 (0.00007) | 0.490 (0.0015) |
|  | Mechanism 2 | 0.1 | -0.047 (0.0008) | 0.0353 (0.00005) | -0.047 (0.0008) | 0.0354 (0.00007) | 0.489 (0.0015) |
|  |  | 0.3 | -0.137 (0.0008) | 0.0349 (0.00005) | -0.137 (0.0008) | 0.0350 (0.00007) | 0.487 (0.0014) |
|  |  | 0.5 | -0.229 (0.0008) | 0.0341 (0.00005) | -0.229 (0.0008) | 0.0342 (0.00006) | 0.490 (0.0014) |
|  |  | 0.7 | -0.323 (0.0009) | 0.0327 (0.00005) | -0.323 (0.0009) | 0.0328 (0.00007) | 0.487 (0.0015) |
|  | Mechanism 3 | 0.1 | -0.047 (0.0008) | 0.0353 (0.00006) | -0.047 (0.0008) | 0.0354 (0.00007) | 0.490 (0.0015) |
|  |  | 0.3 | -0.140 (0.0008) | 0.0350 (0.00005) | -0.140 (0.0008) | 0.0351 (0.00007) | 0.490 (0.0015) |
|  |  | 0.5 | -0.229 (0.0008) | 0.0340 (0.00005) | -0.229 (0.0008) | 0.0342 (0.00006) | 0.490 (0.0014) |
|  |  | 0.7 | -0.323 (0.0008) | 0.0328 (0.00005) | -0.323 (0.0008) | 0.0330 (0.00007) | 0.490 (0.0015) |
| Mechanism 2 | Mechanism 1 | 0.1 | -0.317 (0.0008) | 0.0281 (0.00004) | -0.317 (0.0008) | 0.0283 (0.00006) | 0.620 (0.0013) |
|  |  | 0.3 | -0.317 (0.0008) | 0.0281 (0.00004) | -0.317 (0.0008) | 0.0283 (0.00006) | 0.620 (0.0013) |
|  |  | 0.5 | -0.317 (0.0008) | 0.0281 (0.00004) | -0.317 (0.0008) | 0.0283 (0.00006) | 0.620 (0.0013) |
|  |  | 0.7 | -0.317 (0.0008) | 0.0281 (0.00004) | -0.317 (0.0008) | 0.0283 (0.00006) | 0.620 (0.0013) |
|  | Mechanism 2 | 0.1 | -0.317 (0.0008) | 0.0281 (0.00004) | -0.317 (0.0008) | 0.0283 (0.00006) | 0.621 (0.0013) |
|  |  | 0.3 | -0.317 (0.0008) | 0.0280 (0.00004) | -0.317 (0.0008) | 0.0282 (0.00006) | 0.618 (0.0013) |
|  |  | 0.5 | -0.316 (0.0008) | 0.0281 (0.00004) | -0.316 (0.0008) | 0.0283 (0.00006) | 0.619 (0.0013) |
|  |  | 0.7 | -0.318 (0.0008) | 0.0281 (0.00004) | -0.318 (0.0008) | 0.0283 (0.00006) | 0.620 (0.0013) |
|  | Mechanism 3 | 0.1 | -0.318 (0.0009) | 0.0280 (0.00004) | -0.318 (0.0009) | 0.0281 (0.00006) | 0.617 (0.0014) |
|  |  | 0.3 | -0.318 (0.0008) | 0.0281 (0.00004) | -0.318 (0.0008) | 0.0283 (0.00006) | 0.619 (0.0013) |
|  |  | 0.5 | -0.316 (0.0008) | 0.0281 (0.00004) | -0.316 (0.0008) | 0.0282 (0.00006) | 0.617 (0.0013) |
|  |  | 0.7 | -0.317 (0.0008) | 0.0280 (0.00004) | -0.317 (0.0008) | 0.0282 (0.00006) | 0.620 (0.0013) |
| Mechanism 3 | Mechanism 1 | 0.1 | -0.002 (0.0017) | 0.0597 (0.00010) | -0.002 (0.0017) | 0.0600 (0.00015) | 0.822 (0.0008) |
|  |  | 0.3 | -0.002 (0.0017) | 0.0597 (0.00010) | -0.002 (0.0017) | 0.0600 (0.00015) | 0.822 (0.0008) |
|  |  | 0.5 | -0.002 (0.0017) | 0.0597 (0.00010) | -0.002 (0.0017) | 0.0600 (0.00015) | 0.822 (0.0008) |
|  |  | 0.7 | -0.002 (0.0017) | 0.0597 (0.00010) | -0.002 (0.0017) | 0.0600 (0.00015) | 0.822 (0.0008) |
|  | Mechanism 2 | 0.1 | 0.001 (0.0017) | 0.0597 (0.00010) | 0.001 (0.0017) | 0.0599 (0.00015) | 0.820 (0.0008) |
|  |  | 0.3 | -0.001 (0.0017) | 0.0596 (0.00010) | -0.001 (0.0017) | 0.0597 (0.00014) | 0.820 (0.0008) |
|  |  | 0.5 | 0.003 (0.0016) | 0.0599 (0.00010) | 0.003 (0.0017) | 0.0604 (0.00015) | 0.823 (0.0008) |
|  |  | 0.7 | -0.002 (0.0017) | 0.0597 (0.00010) | -0.003 (0.0017) | 0.0599 (0.00015) | 0.821 (0.0008) |
|  | Mechanism 3 | 0.1 | -0.003 (0.0017) | 0.0596 (0.00009) | -0.003 (0.0017) | 0.0601 (0.00015) | 0.822 (0.0008) |
|  |  | 0.3 | 0.001 (0.0017) | 0.0594 (0.00010) | 0.001 (0.0017) | 0.0598 (0.00015) | 0.821 (0.0008) |
|  |  | 0.5 | -0.001 (0.0017) | 0.0597 (0.00010) | -0.001 (0.0017) | 0.0601 (0.00015) | 0.822 (0.0008) |
|  |  | 0.7 | -0.002 (0.0017) | 0.0595 (0.00010) | -0.002 (0.0017) | 0.0598 (0.00015) | 0.821 (0.0007) |

## **Table S3**: Average bias, standard error and fraction of missing information (FMI) across simulations for multiple imputation models including auxiliary variables.

MCSE = Monte Carlo Standard Error; MI = Multiple imputation.

| Missing outcome mechanism | Missing auxiliary mechanism | Correlation between Z and Y | Proportion of missing auxiliary data | MI model **including** auxiliary variable Z | | |
| --- | --- | --- | --- | --- | --- | --- |
|  |  |  |  | Bias (MCSE) | Standard error (MCSE) | FMI (MCSE) |
| Mechanism 1 | Mechanism 1 | 0.1 | 0 | 0.000 (0.0010) | 0.0399 (0.00009) | 0.591 (0.0015) |
|  |  |  | 10 | -0.007 (0.0010) | 0.0394 (0.00009) | 0.581 (0.0014) |
|  |  |  | 20 | -0.013 (0.0009) | 0.0388 (0.00009) | 0.569 (0.0015) |
|  |  |  | 30 | -0.018 (0.0009) | 0.0383 (0.00008) | 0.559 (0.0015) |
|  |  |  | 40 | -0.024 (0.0009) | 0.0377 (0.00008) | 0.544 (0.0014) |
|  |  |  | 50 | -0.028 (0.0009) | 0.0374 (0.00008) | 0.536 (0.0015) |
|  |  |  | 60 | -0.033 (0.0008) | 0.0369 (0.00008) | 0.525 (0.0015) |
|  |  |  | 70 | -0.036 (0.0008) | 0.0366 (0.00007) | 0.517 (0.0015) |
|  |  |  | 80 | -0.040 (0.0008) | 0.0362 (0.00007) | 0.506 (0.0015) |
|  |  |  | 90 | -0.044 (0.0008) | 0.0358 (0.00007) | 0.496 (0.0015) |
|  |  | 0.3 | 0 | -0.000 (0.0009) | 0.0384 (0.00008) | 0.562 (0.0015) |
|  |  |  | 10 | -0.018 (0.0009) | 0.0381 (0.00008) | 0.559 (0.0015) |
|  |  |  | 20 | -0.036 (0.0009) | 0.0378 (0.00008) | 0.555 (0.0015) |
|  |  |  | 30 | -0.052 (0.0009) | 0.0374 (0.00008) | 0.548 (0.0015) |
|  |  |  | 40 | -0.067 (0.0009) | 0.0370 (0.00007) | 0.541 (0.0015) |
|  |  |  | 50 | -0.081 (0.0009) | 0.0367 (0.00007) | 0.533 (0.0015) |
|  |  |  | 60 | -0.094 (0.0008) | 0.0364 (0.00007) | 0.525 (0.0015) |
|  |  |  | 70 | -0.107 (0.0008) | 0.0360 (0.00007) | 0.517 (0.0015) |
|  |  |  | 80 | -0.119 (0.0008) | 0.0357 (0.00007) | 0.508 (0.0015) |
|  |  |  | 90 | -0.129 (0.0008) | 0.0353 (0.00007) | 0.498 (0.0015) |
|  |  | 0.5 | 0 | -0.000 (0.0008) | 0.0351 (0.00006) | 0.475 (0.0015) |
|  |  |  | 10 | -0.023 (0.0008) | 0.0353 (0.00007) | 0.490 (0.0016) |
|  |  |  | 20 | -0.047 (0.0008) | 0.0353 (0.00007) | 0.497 (0.0015) |
|  |  |  | 30 | -0.072 (0.0008) | 0.0353 (0.00007) | 0.503 (0.0016) |
|  |  |  | 40 | -0.096 (0.0008) | 0.0354 (0.00007) | 0.511 (0.0015) |
|  |  |  | 50 | -0.121 (0.0008) | 0.0352 (0.00007) | 0.512 (0.0016) |
|  |  |  | 60 | -0.146 (0.0008) | 0.0352 (0.00007) | 0.514 (0.0016) |
|  |  |  | 70 | -0.169 (0.0008) | 0.0350 (0.00007) | 0.512 (0.0015) |
|  |  |  | 80 | -0.192 (0.0008) | 0.0347 (0.00007) | 0.503 (0.0015) |
|  |  |  | 90 | -0.213 (0.0008) | 0.0346 (0.00007) | 0.499 (0.0015) |
|  |  | 0.7 | 0 | -0.001 (0.0005) | 0.0294 (0.00004) | 0.256 (0.0012) |
|  |  |  | 10 | -0.018 (0.0005) | 0.0300 (0.00004) | 0.286 (0.0013) |
|  |  |  | 20 | -0.038 (0.0005) | 0.0304 (0.00004) | 0.313 (0.0014) |
|  |  |  | 30 | -0.061 (0.0006) | 0.0309 (0.00005) | 0.341 (0.0015) |
|  |  |  | 40 | -0.087 (0.0006) | 0.0314 (0.00005) | 0.370 (0.0015) |
|  |  |  | 50 | -0.118 (0.0007) | 0.0319 (0.00005) | 0.402 (0.0015) |
|  |  |  | 60 | -0.154 (0.0007) | 0.0324 (0.00006) | 0.434 (0.0016) |
|  |  |  | 70 | -0.194 (0.0008) | 0.0329 (0.00006) | 0.464 (0.0017) |
|  |  |  | 80 | -0.238 (0.0008) | 0.0331 (0.00007) | 0.483 (0.0016) |
|  |  |  | 90 | -0.283 (0.0008) | 0.0331 (0.00007) | 0.493 (0.0016) |
|  | Mechanism 2 | 0.1 | 0 | -0.002 (0.0009) | 0.0398 (0.00009) | 0.593 (0.0015) |
|  |  |  | 10 | -0.008 (0.0010) | 0.0399 (0.00009) | 0.596 (0.0015) |
|  |  |  | 20 | -0.013 (0.0009) | 0.0394 (0.00008) | 0.586 (0.0014) |
|  |  |  | 30 | -0.018 (0.0009) | 0.0389 (0.00008) | 0.574 (0.0015) |
|  |  |  | 40 | -0.024 (0.0009) | 0.0382 (0.00008) | 0.558 (0.0016) |
|  |  |  | 50 | -0.031 (0.0008) | 0.0372 (0.00008) | 0.535 (0.0016) |
|  |  |  | 60 | -0.038 (0.0008) | 0.0361 (0.00007) | 0.507 (0.0015) |
|  |  |  | 70 | -0.046 (0.0008) | 0.0355 (0.00007) | 0.489 (0.0015) |
|  |  |  | 80 | -0.047 (0.0008) | 0.0354 (0.00007) | 0.489 (0.0015) |
|  |  |  | 90 | -0.047 (0.0008) | 0.0354 (0.00006) | 0.489 (0.0015) |
|  |  | 0.3 | 0 | 0.001 (0.0009) | 0.0384 (0.00008) | 0.560 (0.0015) |
|  |  |  | 10 | -0.016 (0.0009) | 0.0385 (0.00008) | 0.567 (0.0015) |
|  |  |  | 20 | -0.031 (0.0009) | 0.0384 (0.00008) | 0.565 (0.0015) |
|  |  |  | 30 | -0.047 (0.0009) | 0.0379 (0.00008) | 0.555 (0.0016) |
|  |  |  | 40 | -0.065 (0.0008) | 0.0373 (0.00008) | 0.542 (0.0015) |
|  |  |  | 50 | -0.085 (0.0008) | 0.0364 (0.00007) | 0.521 (0.0015) |
|  |  |  | 60 | -0.108 (0.0008) | 0.0356 (0.00007) | 0.499 (0.0015) |
|  |  |  | 70 | -0.135 (0.0008) | 0.0351 (0.00007) | 0.488 (0.0015) |
|  |  |  | 80 | -0.137 (0.0008) | 0.0351 (0.00007) | 0.487 (0.0014) |
|  |  |  | 90 | -0.137 (0.0008) | 0.0350 (0.00006) | 0.487 (0.0014) |
|  |  | 0.5 | 0 | 0.002 (0.0008) | 0.0352 (0.00007) | 0.477 (0.0016) |
|  |  |  | 10 | -0.021 (0.0008) | 0.0352 (0.00007) | 0.497 (0.0015) |
|  |  |  | 20 | -0.045 (0.0008) | 0.0352 (0.00007) | 0.504 (0.0016) |
|  |  |  | 30 | -0.071 (0.0008) | 0.0351 (0.00007) | 0.503 (0.0016) |
|  |  |  | 40 | -0.101 (0.0008) | 0.0349 (0.00007) | 0.499 (0.0015) |
|  |  |  | 50 | -0.136 (0.0008) | 0.0346 (0.00006) | 0.493 (0.0015) |
|  |  |  | 60 | -0.177 (0.0008) | 0.0342 (0.00007) | 0.486 (0.0014) |
|  |  |  | 70 | -0.226 (0.0008) | 0.0342 (0.00007) | 0.488 (0.0015) |
|  |  |  | 80 | -0.229 (0.0008) | 0.0342 (0.00006) | 0.489 (0.0015) |
|  |  |  | 90 | -0.229 (0.0008) | 0.0342 (0.00006) | 0.490 (0.0014) |
|  |  | 0.7 | 0 | -0.000 (0.0005) | 0.0295 (0.00004) | 0.262 (0.0012) |
|  |  |  | 10 | -0.034 (0.0006) | 0.0288 (0.00004) | 0.307 (0.0013) |
|  |  |  | 20 | -0.065 (0.0006) | 0.0289 (0.00004) | 0.337 (0.0013) |
|  |  |  | 30 | -0.100 (0.0007) | 0.0291 (0.00004) | 0.359 (0.0014) |
|  |  |  | 40 | -0.140 (0.0007) | 0.0295 (0.00005) | 0.380 (0.0015) |
|  |  |  | 50 | -0.187 (0.0008) | 0.0301 (0.00005) | 0.405 (0.0014) |
|  |  |  | 60 | -0.245 (0.0008) | 0.0311 (0.00006) | 0.439 (0.0014) |
|  |  |  | 70 | -0.317 (0.0009) | 0.0328 (0.00007) | 0.487 (0.0015) |
|  |  |  | 80 | -0.323 (0.0009) | 0.0328 (0.00007) | 0.489 (0.0015) |
|  |  |  | 90 | -0.323 (0.0009) | 0.0329 (0.00007) | 0.491 (0.0014) |
|  | Mechanism 3 | 0.1 | 0 | -0.002 (0.0010) | 0.0400 (0.00009) | 0.597 (0.0014) |
|  |  |  | 10 | -0.003 (0.0010) | 0.0398 (0.00009) | 0.594 (0.0014) |
|  |  |  | 20 | -0.007 (0.0010) | 0.0396 (0.00009) | 0.588 (0.0015) |
|  |  |  | 30 | -0.011 (0.0010) | 0.0391 (0.00009) | 0.580 (0.0015) |
|  |  |  | 40 | -0.015 (0.0010) | 0.0388 (0.00008) | 0.572 (0.0015) |
|  |  |  | 50 | -0.020 (0.0009) | 0.0384 (0.00008) | 0.565 (0.0014) |
|  |  |  | 60 | -0.024 (0.0009) | 0.0381 (0.00008) | 0.557 (0.0015) |
|  |  |  | 70 | -0.027 (0.0009) | 0.0378 (0.00008) | 0.550 (0.0015) |
|  |  |  | 80 | -0.031 (0.0009) | 0.0376 (0.00008) | 0.546 (0.0015) |
|  |  |  | 90 | -0.036 (0.0009) | 0.0377 (0.00008) | 0.548 (0.0015) |
|  |  | 0.3 | 0 | -0.001 (0.0009) | 0.0385 (0.00008) | 0.563 (0.0015) |
|  |  |  | 10 | -0.007 (0.0009) | 0.0385 (0.00008) | 0.565 (0.0014) |
|  |  |  | 20 | -0.017 (0.0009) | 0.0384 (0.00008) | 0.565 (0.0015) |
|  |  |  | 30 | -0.028 (0.0009) | 0.0382 (0.00008) | 0.562 (0.0015) |
|  |  |  | 40 | -0.041 (0.0009) | 0.0378 (0.00008) | 0.557 (0.0015) |
|  |  |  | 50 | -0.054 (0.0009) | 0.0376 (0.00008) | 0.553 (0.0015) |
|  |  |  | 60 | -0.067 (0.0009) | 0.0373 (0.00007) | 0.547 (0.0015) |
|  |  |  | 70 | -0.079 (0.0009) | 0.0370 (0.00008) | 0.541 (0.0016) |
|  |  |  | 80 | -0.092 (0.0009) | 0.0370 (0.00007) | 0.542 (0.0014) |
|  |  |  | 90 | -0.105 (0.0009) | 0.0378 (0.00008) | 0.560 (0.0015) |
|  |  | 0.5 | 0 | 0.001 (0.0008) | 0.0351 (0.00006) | 0.479 (0.0015) |
|  |  |  | 10 | -0.007 (0.0008) | 0.0353 (0.00007) | 0.489 (0.0016) |
|  |  |  | 20 | -0.021 (0.0008) | 0.0355 (0.00007) | 0.500 (0.0016) |
|  |  |  | 30 | -0.038 (0.0008) | 0.0355 (0.00007) | 0.508 (0.0016) |
|  |  |  | 40 | -0.057 (0.0008) | 0.0354 (0.00007) | 0.512 (0.0015) |
|  |  |  | 50 | -0.078 (0.0008) | 0.0352 (0.00007) | 0.514 (0.0015) |
|  |  |  | 60 | -0.100 (0.0008) | 0.0352 (0.00007) | 0.518 (0.0016) |
|  |  |  | 70 | -0.122 (0.0008) | 0.0352 (0.00007) | 0.520 (0.0015) |
|  |  |  | 80 | -0.144 (0.0008) | 0.0355 (0.00007) | 0.529 (0.0015) |
|  |  |  | 90 | -0.169 (0.0009) | 0.0376 (0.00010) | 0.576 (0.0018) |
|  |  | 0.7 | 0 | 0.001 (0.0005) | 0.0295 (0.00004) | 0.260 (0.0012) |
|  |  |  | 10 | -0.005 (0.0005) | 0.0300 (0.00004) | 0.288 (0.0013) |
|  |  |  | 20 | -0.016 (0.0005) | 0.0304 (0.00005) | 0.315 (0.0014) |
|  |  |  | 30 | -0.031 (0.0006) | 0.0308 (0.00005) | 0.344 (0.0014) |
|  |  |  | 40 | -0.050 (0.0006) | 0.0312 (0.00005) | 0.374 (0.0014) |
|  |  |  | 50 | -0.076 (0.0007) | 0.0316 (0.00005) | 0.401 (0.0015) |
|  |  |  | 60 | -0.106 (0.0007) | 0.0320 (0.00006) | 0.431 (0.0015) |
|  |  |  | 70 | -0.141 (0.0008) | 0.0326 (0.00006) | 0.460 (0.0015) |
|  |  |  | 80 | -0.184 (0.0009) | 0.0337 (0.00008) | 0.502 (0.0019) |
|  |  |  | 90 | -0.234 (0.0014) | 0.0385 (0.00019) | 0.600 (0.0030) |
| Mechanism 2 | Mechanism 1 | 0.1 | 0 | -0.315 (0.0008) | 0.0282 (0.00006) | 0.618 (0.0012) |
|  |  |  | 10 | -0.315 (0.0008) | 0.0282 (0.00006) | 0.618 (0.0014) |
|  |  |  | 20 | -0.315 (0.0008) | 0.0282 (0.00006) | 0.619 (0.0013) |
|  |  |  | 30 | -0.316 (0.0008) | 0.0282 (0.00006) | 0.618 (0.0013) |
|  |  |  | 40 | -0.316 (0.0008) | 0.0283 (0.00006) | 0.620 (0.0013) |
|  |  |  | 50 | -0.316 (0.0008) | 0.0282 (0.00006) | 0.618 (0.0013) |
|  |  |  | 60 | -0.316 (0.0008) | 0.0282 (0.00006) | 0.618 (0.0013) |
|  |  |  | 70 | -0.317 (0.0008) | 0.0283 (0.00006) | 0.619 (0.0013) |
|  |  |  | 80 | -0.317 (0.0008) | 0.0283 (0.00006) | 0.618 (0.0014) |
|  |  |  | 90 | -0.317 (0.0008) | 0.0283 (0.00006) | 0.619 (0.0014) |
|  |  | 0.3 | 0 | -0.295 (0.0008) | 0.0279 (0.00006) | 0.607 (0.0013) |
|  |  |  | 10 | -0.297 (0.0008) | 0.0280 (0.00006) | 0.608 (0.0014) |
|  |  |  | 20 | -0.299 (0.0008) | 0.0280 (0.00006) | 0.611 (0.0013) |
|  |  |  | 30 | -0.301 (0.0008) | 0.0280 (0.00006) | 0.611 (0.0013) |
|  |  |  | 40 | -0.304 (0.0008) | 0.0281 (0.00006) | 0.614 (0.0013) |
|  |  |  | 50 | -0.306 (0.0008) | 0.0281 (0.00006) | 0.614 (0.0013) |
|  |  |  | 60 | -0.308 (0.0008) | 0.0281 (0.00006) | 0.614 (0.0013) |
|  |  |  | 70 | -0.310 (0.0008) | 0.0282 (0.00006) | 0.617 (0.0013) |
|  |  |  | 80 | -0.313 (0.0008) | 0.0282 (0.00006) | 0.617 (0.0014) |
|  |  |  | 90 | -0.315 (0.0008) | 0.0283 (0.00006) | 0.618 (0.0014) |
|  |  | 0.5 | 0 | -0.243 (0.0007) | 0.0272 (0.00005) | 0.564 (0.0013) |
|  |  |  | 10 | -0.250 (0.0007) | 0.0273 (0.00006) | 0.572 (0.0014) |
|  |  |  | 20 | -0.257 (0.0008) | 0.0275 (0.00005) | 0.581 (0.0014) |
|  |  |  | 30 | -0.265 (0.0008) | 0.0276 (0.00006) | 0.587 (0.0013) |
|  |  |  | 40 | -0.272 (0.0008) | 0.0277 (0.00006) | 0.596 (0.0014) |
|  |  |  | 50 | -0.280 (0.0008) | 0.0279 (0.00006) | 0.601 (0.0014) |
|  |  |  | 60 | -0.287 (0.0008) | 0.0279 (0.00006) | 0.605 (0.0014) |
|  |  |  | 70 | -0.295 (0.0008) | 0.0281 (0.00006) | 0.612 (0.0013) |
|  |  |  | 80 | -0.302 (0.0008) | 0.0281 (0.00006) | 0.614 (0.0014) |
|  |  |  | 90 | -0.310 (0.0008) | 0.0282 (0.00006) | 0.617 (0.0014) |
|  |  | 0.7 | 0 | -0.125 (0.0005) | 0.0259 (0.00004) | 0.363 (0.0013) |
|  |  |  | 10 | -0.135 (0.0006) | 0.0263 (0.00004) | 0.398 (0.0014) |
|  |  |  | 20 | -0.146 (0.0006) | 0.0267 (0.00004) | 0.433 (0.0015) |
|  |  |  | 30 | -0.161 (0.0006) | 0.0270 (0.00005) | 0.470 (0.0014) |
|  |  |  | 40 | -0.178 (0.0007) | 0.0275 (0.00005) | 0.506 (0.0015) |
|  |  |  | 50 | -0.198 (0.0007) | 0.0278 (0.00006) | 0.539 (0.0015) |
|  |  |  | 60 | -0.222 (0.0008) | 0.0281 (0.00006) | 0.568 (0.0015) |
|  |  |  | 70 | -0.246 (0.0008) | 0.0284 (0.00006) | 0.594 (0.0014) |
|  |  |  | 80 | -0.272 (0.0008) | 0.0284 (0.00006) | 0.608 (0.0014) |
|  |  |  | 90 | -0.296 (0.0008) | 0.0284 (0.00006) | 0.617 (0.0014) |
|  | Mechanism 2 | 0.1 | 0 | -0.315 (0.0008) | 0.0282 (0.00006) | 0.618 (0.0012) |
|  |  |  | 10 | -0.315 (0.0008) | 0.0283 (0.00006) | 0.620 (0.0013) |
|  |  |  | 20 | -0.316 (0.0008) | 0.0283 (0.00006) | 0.620 (0.0012) |
|  |  |  | 30 | -0.316 (0.0008) | 0.0283 (0.00006) | 0.620 (0.0013) |
|  |  |  | 40 | -0.316 (0.0008) | 0.0282 (0.00006) | 0.617 (0.0013) |
|  |  |  | 50 | -0.317 (0.0008) | 0.0283 (0.00006) | 0.621 (0.0013) |
|  |  |  | 60 | -0.317 (0.0008) | 0.0283 (0.00006) | 0.619 (0.0013) |
|  |  |  | 70 | -0.317 (0.0008) | 0.0283 (0.00006) | 0.621 (0.0012) |
|  |  |  | 80 | -0.317 (0.0008) | 0.0283 (0.00006) | 0.620 (0.0013) |
|  |  |  | 90 | -0.317 (0.0008) | 0.0283 (0.00006) | 0.621 (0.0012) |
|  |  | 0.3 | 0 | -0.294 (0.0008) | 0.0278 (0.00006) | 0.605 (0.0013) |
|  |  |  | 10 | -0.299 (0.0008) | 0.0279 (0.00006) | 0.610 (0.0013) |
|  |  |  | 20 | -0.303 (0.0008) | 0.0279 (0.00006) | 0.610 (0.0014) |
|  |  |  | 30 | -0.306 (0.0008) | 0.0280 (0.00006) | 0.613 (0.0013) |
|  |  |  | 40 | -0.308 (0.0008) | 0.0279 (0.00006) | 0.612 (0.0013) |
|  |  |  | 50 | -0.310 (0.0008) | 0.0280 (0.00006) | 0.615 (0.0013) |
|  |  |  | 60 | -0.312 (0.0008) | 0.0280 (0.00006) | 0.613 (0.0013) |
|  |  |  | 70 | -0.314 (0.0008) | 0.0281 (0.00006) | 0.616 (0.0013) |
|  |  |  | 80 | -0.315 (0.0008) | 0.0281 (0.00006) | 0.615 (0.0013) |
|  |  |  | 90 | -0.316 (0.0008) | 0.0281 (0.00006) | 0.614 (0.0013) |
|  |  | 0.5 | 0 | -0.243 (0.0007) | 0.0272 (0.00005) | 0.564 (0.0015) |
|  |  |  | 10 | -0.252 (0.0008) | 0.0269 (0.00005) | 0.578 (0.0014) |
|  |  |  | 20 | -0.262 (0.0008) | 0.0271 (0.00005) | 0.589 (0.0014) |
|  |  |  | 30 | -0.271 (0.0008) | 0.0272 (0.00005) | 0.593 (0.0014) |
|  |  |  | 40 | -0.281 (0.0008) | 0.0274 (0.00006) | 0.599 (0.0014) |
|  |  |  | 50 | -0.289 (0.0008) | 0.0274 (0.00006) | 0.600 (0.0014) |
|  |  |  | 60 | -0.297 (0.0008) | 0.0277 (0.00006) | 0.607 (0.0014) |
|  |  |  | 70 | -0.304 (0.0008) | 0.0279 (0.00006) | 0.611 (0.0013) |
|  |  |  | 80 | -0.310 (0.0008) | 0.0280 (0.00006) | 0.614 (0.0014) |
|  |  |  | 90 | -0.314 (0.0008) | 0.0281 (0.00006) | 0.615 (0.0013) |
|  |  | 0.7 | 0 | -0.125 (0.0005) | 0.0259 (0.00004) | 0.361 (0.0013) |
|  |  |  | 10 | -0.134 (0.0006) | 0.0248 (0.00004) | 0.434 (0.0015) |
|  |  |  | 20 | -0.152 (0.0007) | 0.0245 (0.00004) | 0.467 (0.0014) |
|  |  |  | 30 | -0.174 (0.0007) | 0.0245 (0.00004) | 0.487 (0.0014) |
|  |  |  | 40 | -0.200 (0.0007) | 0.0247 (0.00004) | 0.508 (0.0014) |
|  |  |  | 50 | -0.227 (0.0008) | 0.0251 (0.00004) | 0.528 (0.0014) |
|  |  |  | 60 | -0.255 (0.0008) | 0.0257 (0.00005) | 0.548 (0.0014) |
|  |  |  | 70 | -0.280 (0.0008) | 0.0264 (0.00005) | 0.570 (0.0013) |
|  |  |  | 80 | -0.301 (0.0008) | 0.0272 (0.00005) | 0.592 (0.0013) |
|  |  |  | 90 | -0.315 (0.0008) | 0.0280 (0.00006) | 0.612 (0.0014) |
|  | Mechanism 3 | 0.1 | 0 | -0.315 (0.0009) | 0.0281 (0.00006) | 0.616 (0.0013) |
|  |  |  | 10 | -0.315 (0.0009) | 0.0281 (0.00006) | 0.616 (0.0013) |
|  |  |  | 20 | -0.316 (0.0009) | 0.0280 (0.00006) | 0.615 (0.0013) |
|  |  |  | 30 | -0.316 (0.0009) | 0.0281 (0.00006) | 0.615 (0.0013) |
|  |  |  | 40 | -0.316 (0.0009) | 0.0281 (0.00006) | 0.615 (0.0013) |
|  |  |  | 50 | -0.316 (0.0009) | 0.0281 (0.00006) | 0.615 (0.0014) |
|  |  |  | 60 | -0.316 (0.0009) | 0.0281 (0.00006) | 0.617 (0.0013) |
|  |  |  | 70 | -0.316 (0.0009) | 0.0281 (0.00006) | 0.618 (0.0014) |
|  |  |  | 80 | -0.317 (0.0009) | 0.0281 (0.00006) | 0.617 (0.0014) |
|  |  |  | 90 | -0.317 (0.0009) | 0.0281 (0.00006) | 0.616 (0.0013) |
|  |  | 0.3 | 0 | -0.295 (0.0008) | 0.0280 (0.00006) | 0.608 (0.0013) |
|  |  |  | 10 | -0.296 (0.0008) | 0.0280 (0.00006) | 0.610 (0.0013) |
|  |  |  | 20 | -0.298 (0.0008) | 0.0280 (0.00006) | 0.611 (0.0013) |
|  |  |  | 30 | -0.299 (0.0008) | 0.0281 (0.00006) | 0.614 (0.0013) |
|  |  |  | 40 | -0.301 (0.0008) | 0.0280 (0.00006) | 0.613 (0.0013) |
|  |  |  | 50 | -0.303 (0.0008) | 0.0281 (0.00006) | 0.616 (0.0013) |
|  |  |  | 60 | -0.305 (0.0008) | 0.0281 (0.00006) | 0.616 (0.0013) |
|  |  |  | 70 | -0.307 (0.0008) | 0.0281 (0.00006) | 0.614 (0.0013) |
|  |  |  | 80 | -0.309 (0.0008) | 0.0282 (0.00006) | 0.618 (0.0013) |
|  |  |  | 90 | -0.311 (0.0008) | 0.0283 (0.00006) | 0.619 (0.0013) |
|  |  | 0.5 | 0 | -0.242 (0.0008) | 0.0272 (0.00005) | 0.562 (0.0014) |
|  |  |  | 10 | -0.246 (0.0008) | 0.0272 (0.00005) | 0.572 (0.0014) |
|  |  |  | 20 | -0.250 (0.0008) | 0.0273 (0.00006) | 0.580 (0.0014) |
|  |  |  | 30 | -0.255 (0.0008) | 0.0274 (0.00006) | 0.587 (0.0014) |
|  |  |  | 40 | -0.261 (0.0008) | 0.0274 (0.00006) | 0.592 (0.0013) |
|  |  |  | 50 | -0.266 (0.0008) | 0.0275 (0.00006) | 0.595 (0.0014) |
|  |  |  | 60 | -0.272 (0.0008) | 0.0277 (0.00006) | 0.602 (0.0014) |
|  |  |  | 70 | -0.278 (0.0008) | 0.0277 (0.00006) | 0.604 (0.0014) |
|  |  |  | 80 | -0.285 (0.0008) | 0.0279 (0.00006) | 0.609 (0.0014) |
|  |  |  | 90 | -0.294 (0.0009) | 0.0284 (0.00006) | 0.622 (0.0014) |
|  |  | 0.7 | 0 | -0.125 (0.0005) | 0.0258 (0.00004) | 0.362 (0.0014) |
|  |  |  | 10 | -0.127 (0.0006) | 0.0260 (0.00004) | 0.397 (0.0014) |
|  |  |  | 20 | -0.132 (0.0006) | 0.0263 (0.00004) | 0.437 (0.0014) |
|  |  |  | 30 | -0.141 (0.0007) | 0.0264 (0.00005) | 0.469 (0.0015) |
|  |  |  | 40 | -0.153 (0.0007) | 0.0266 (0.00005) | 0.500 (0.0015) |
|  |  |  | 50 | -0.169 (0.0007) | 0.0268 (0.00005) | 0.527 (0.0014) |
|  |  |  | 60 | -0.187 (0.0008) | 0.0270 (0.00005) | 0.552 (0.0014) |
|  |  |  | 70 | -0.208 (0.0008) | 0.0272 (0.00005) | 0.572 (0.0014) |
|  |  |  | 80 | -0.232 (0.0008) | 0.0277 (0.00006) | 0.593 (0.0015) |
|  |  |  | 90 | -0.264 (0.0009) | 0.0295 (0.00009) | 0.642 (0.0019) |
| Mechanism 3 | Mechanism 1 | 0.1 | 0 | -0.002 (0.0017) | 0.0595 (0.00015) | 0.819 (0.0008) |
|  |  |  | 10 | -0.002 (0.0017) | 0.0598 (0.00015) | 0.820 (0.0008) |
|  |  |  | 20 | -0.002 (0.0017) | 0.0598 (0.00015) | 0.821 (0.0008) |
|  |  |  | 30 | -0.002 (0.0017) | 0.0599 (0.00015) | 0.821 (0.0008) |
|  |  |  | 40 | -0.002 (0.0017) | 0.0599 (0.00015) | 0.821 (0.0008) |
|  |  |  | 50 | -0.002 (0.0017) | 0.0599 (0.00015) | 0.821 (0.0008) |
|  |  |  | 60 | -0.002 (0.0017) | 0.0601 (0.00015) | 0.822 (0.0008) |
|  |  |  | 70 | -0.002 (0.0017) | 0.0599 (0.00015) | 0.821 (0.0008) |
|  |  |  | 80 | -0.003 (0.0017) | 0.0600 (0.00015) | 0.821 (0.0008) |
|  |  |  | 90 | -0.002 (0.0017) | 0.0602 (0.00015) | 0.823 (0.0008) |
|  |  | 0.3 | 0 | -0.002 (0.0016) | 0.0563 (0.00014) | 0.797 (0.0009) |
|  |  |  | 10 | -0.002 (0.0016) | 0.0569 (0.00014) | 0.801 (0.0009) |
|  |  |  | 20 | -0.002 (0.0016) | 0.0572 (0.00014) | 0.804 (0.0008) |
|  |  |  | 30 | -0.002 (0.0016) | 0.0576 (0.00014) | 0.806 (0.0008) |
|  |  |  | 40 | -0.002 (0.0017) | 0.0578 (0.00014) | 0.808 (0.0008) |
|  |  |  | 50 | -0.002 (0.0017) | 0.0583 (0.00015) | 0.811 (0.0008) |
|  |  |  | 60 | -0.002 (0.0017) | 0.0587 (0.00015) | 0.814 (0.0008) |
|  |  |  | 70 | -0.002 (0.0017) | 0.0589 (0.00015) | 0.815 (0.0008) |
|  |  |  | 80 | -0.003 (0.0017) | 0.0593 (0.00015) | 0.817 (0.0008) |
|  |  |  | 90 | -0.002 (0.0017) | 0.0598 (0.00015) | 0.821 (0.0008) |
|  |  | 0.5 | 0 | -0.002 (0.0014) | 0.0494 (0.00011) | 0.736 (0.0011) |
|  |  |  | 10 | -0.002 (0.0014) | 0.0503 (0.00012) | 0.746 (0.0011) |
|  |  |  | 20 | -0.002 (0.0014) | 0.0512 (0.00012) | 0.755 (0.0010) |
|  |  |  | 30 | -0.002 (0.0015) | 0.0521 (0.00012) | 0.763 (0.0010) |
|  |  |  | 40 | -0.002 (0.0015) | 0.0529 (0.00013) | 0.770 (0.0010) |
|  |  |  | 50 | -0.002 (0.0015) | 0.0540 (0.00013) | 0.780 (0.0010) |
|  |  |  | 60 | -0.002 (0.0016) | 0.0551 (0.00013) | 0.789 (0.0009) |
|  |  |  | 70 | -0.002 (0.0016) | 0.0562 (0.00014) | 0.796 (0.0009) |
|  |  |  | 80 | -0.003 (0.0016) | 0.0574 (0.00014) | 0.805 (0.0009) |
|  |  |  | 90 | -0.003 (0.0017) | 0.0589 (0.00014) | 0.815 (0.0008) |
|  |  | 0.7 | 0 | -0.001 (0.0009) | 0.0365 (0.00006) | 0.517 (0.0014) |
|  |  |  | 10 | -0.001 (0.0009) | 0.0376 (0.00007) | 0.545 (0.0014) |
|  |  |  | 20 | -0.001 (0.0010) | 0.0389 (0.00007) | 0.574 (0.0014) |
|  |  |  | 30 | -0.001 (0.0010) | 0.0402 (0.00008) | 0.601 (0.0013) |
|  |  |  | 40 | -0.001 (0.0011) | 0.0417 (0.00009) | 0.629 (0.0013) |
|  |  |  | 50 | -0.002 (0.0011) | 0.0436 (0.00009) | 0.661 (0.0013) |
|  |  |  | 60 | -0.001 (0.0012) | 0.0456 (0.00010) | 0.691 (0.0012) |
|  |  |  | 70 | -0.002 (0.0013) | 0.0482 (0.00011) | 0.723 (0.0011) |
|  |  |  | 80 | -0.002 (0.0014) | 0.0512 (0.00012) | 0.754 (0.0010) |
|  |  |  | 90 | -0.002 (0.0016) | 0.0554 (0.00014) | 0.791 (0.0009) |
|  | Mechanism 2 | 0.1 | 0 | 0.001 (0.0017) | 0.0596 (0.00015) | 0.818 (0.0008) |
|  |  |  | 10 | 0.001 (0.0017) | 0.0599 (0.00015) | 0.820 (0.0008) |
|  |  |  | 20 | 0.001 (0.0017) | 0.0600 (0.00015) | 0.821 (0.0008) |
|  |  |  | 30 | 0.001 (0.0017) | 0.0600 (0.00015) | 0.821 (0.0008) |
|  |  |  | 40 | 0.001 (0.0017) | 0.0601 (0.00015) | 0.821 (0.0008) |
|  |  |  | 50 | 0.001 (0.0017) | 0.0602 (0.00015) | 0.822 (0.0008) |
|  |  |  | 60 | 0.001 (0.0017) | 0.0602 (0.00015) | 0.822 (0.0008) |
|  |  |  | 70 | 0.001 (0.0017) | 0.0601 (0.00015) | 0.821 (0.0008) |
|  |  |  | 80 | 0.001 (0.0017) | 0.0601 (0.00014) | 0.822 (0.0008) |
|  |  |  | 90 | 0.001 (0.0017) | 0.0600 (0.00015) | 0.821 (0.0008) |
|  |  | 0.3 | 0 | -0.001 (0.0016) | 0.0564 (0.00014) | 0.798 (0.0009) |
|  |  |  | 10 | -0.003 (0.0016) | 0.0575 (0.00014) | 0.806 (0.0008) |
|  |  |  | 20 | -0.004 (0.0016) | 0.0581 (0.00014) | 0.810 (0.0008) |
|  |  |  | 30 | -0.005 (0.0016) | 0.0587 (0.00015) | 0.813 (0.0008) |
|  |  |  | 40 | -0.005 (0.0017) | 0.0591 (0.00014) | 0.817 (0.0008) |
|  |  |  | 50 | -0.004 (0.0016) | 0.0593 (0.00015) | 0.817 (0.0008) |
|  |  |  | 60 | -0.004 (0.0017) | 0.0596 (0.00015) | 0.819 (0.0008) |
|  |  |  | 70 | -0.003 (0.0017) | 0.0598 (0.00015) | 0.821 (0.0008) |
|  |  |  | 80 | -0.002 (0.0017) | 0.0598 (0.00015) | 0.820 (0.0008) |
|  |  |  | 90 | -0.002 (0.0017) | 0.0599 (0.00015) | 0.821 (0.0008) |
|  |  | 0.5 | 0 | 0.002 (0.0013) | 0.0497 (0.00011) | 0.739 (0.0011) |
|  |  |  | 10 | -0.010 (0.0014) | 0.0517 (0.00012) | 0.760 (0.0010) |
|  |  |  | 20 | -0.015 (0.0014) | 0.0535 (0.00013) | 0.776 (0.0010) |
|  |  |  | 30 | -0.018 (0.0015) | 0.0550 (0.00013) | 0.788 (0.0009) |
|  |  |  | 40 | -0.018 (0.0015) | 0.0563 (0.00013) | 0.797 (0.0009) |
|  |  |  | 50 | -0.017 (0.0015) | 0.0575 (0.00014) | 0.806 (0.0008) |
|  |  |  | 60 | -0.014 (0.0016) | 0.0584 (0.00014) | 0.811 (0.0008) |
|  |  |  | 70 | -0.010 (0.0016) | 0.0592 (0.00014) | 0.816 (0.0008) |
|  |  |  | 80 | -0.005 (0.0016) | 0.0596 (0.00015) | 0.818 (0.0008) |
|  |  |  | 90 | -0.001 (0.0016) | 0.0600 (0.00015) | 0.821 (0.0008) |
|  |  | 0.7 | 0 | -0.001 (0.0008) | 0.0365 (0.00006) | 0.516 (0.0014) |
|  |  |  | 10 | -0.039 (0.0009) | 0.0379 (0.00007) | 0.576 (0.0014) |
|  |  |  | 20 | -0.066 (0.0010) | 0.0399 (0.00008) | 0.619 (0.0014) |
|  |  |  | 30 | -0.085 (0.0011) | 0.0421 (0.00009) | 0.658 (0.0013) |
|  |  |  | 40 | -0.097 (0.0012) | 0.0449 (0.00010) | 0.695 (0.0012) |
|  |  |  | 50 | -0.101 (0.0013) | 0.0483 (0.00012) | 0.733 (0.0012) |
|  |  |  | 60 | -0.095 (0.0015) | 0.0520 (0.00013) | 0.766 (0.0011) |
|  |  |  | 70 | -0.076 (0.0016) | 0.0561 (0.00014) | 0.797 (0.0009) |
|  |  |  | 80 | -0.048 (0.0017) | 0.0592 (0.00014) | 0.817 (0.0008) |
|  |  |  | 90 | -0.019 (0.0017) | 0.0602 (0.00015) | 0.823 (0.0008) |
|  | Mechanism 3 | 0.1 | 0 | -0.003 (0.0017) | 0.0596 (0.00015) | 0.819 (0.0008) |
|  |  |  | 10 | -0.002 (0.0017) | 0.0596 (0.00014) | 0.819 (0.0008) |
|  |  |  | 20 | -0.003 (0.0017) | 0.0598 (0.00015) | 0.820 (0.0008) |
|  |  |  | 30 | -0.002 (0.0017) | 0.0597 (0.00015) | 0.820 (0.0008) |
|  |  |  | 40 | -0.003 (0.0017) | 0.0599 (0.00015) | 0.821 (0.0008) |
|  |  |  | 50 | -0.002 (0.0017) | 0.0596 (0.00014) | 0.819 (0.0008) |
|  |  |  | 60 | -0.002 (0.0017) | 0.0597 (0.00015) | 0.820 (0.0008) |
|  |  |  | 70 | -0.002 (0.0017) | 0.0598 (0.00014) | 0.820 (0.0008) |
|  |  |  | 80 | -0.003 (0.0017) | 0.0597 (0.00015) | 0.820 (0.0008) |
|  |  |  | 90 | -0.002 (0.0017) | 0.0598 (0.00015) | 0.821 (0.0008) |
|  |  | 0.3 | 0 | -0.000 (0.0016) | 0.0564 (0.00014) | 0.799 (0.0009) |
|  |  |  | 10 | -0.001 (0.0016) | 0.0565 (0.00014) | 0.799 (0.0009) |
|  |  |  | 20 | -0.000 (0.0016) | 0.0566 (0.00014) | 0.800 (0.0009) |
|  |  |  | 30 | 0.000 (0.0016) | 0.0570 (0.00014) | 0.803 (0.0009) |
|  |  |  | 40 | -0.000 (0.0016) | 0.0575 (0.00014) | 0.806 (0.0008) |
|  |  |  | 50 | 0.000 (0.0016) | 0.0574 (0.00014) | 0.806 (0.0009) |
|  |  |  | 60 | 0.000 (0.0016) | 0.0576 (0.00014) | 0.807 (0.0008) |
|  |  |  | 70 | -0.000 (0.0016) | 0.0580 (0.00014) | 0.810 (0.0008) |
|  |  |  | 80 | 0.000 (0.0016) | 0.0584 (0.00014) | 0.812 (0.0008) |
|  |  |  | 90 | -0.000 (0.0017) | 0.0588 (0.00015) | 0.815 (0.0008) |
|  |  | 0.5 | 0 | -0.001 (0.0013) | 0.0495 (0.00011) | 0.737 (0.0011) |
|  |  |  | 10 | -0.000 (0.0013) | 0.0500 (0.00011) | 0.743 (0.0010) |
|  |  |  | 20 | -0.001 (0.0014) | 0.0508 (0.00012) | 0.750 (0.0010) |
|  |  |  | 30 | -0.001 (0.0014) | 0.0516 (0.00012) | 0.758 (0.0010) |
|  |  |  | 40 | -0.001 (0.0014) | 0.0524 (0.00012) | 0.765 (0.0010) |
|  |  |  | 50 | -0.002 (0.0015) | 0.0530 (0.00013) | 0.771 (0.0010) |
|  |  |  | 60 | -0.001 (0.0015) | 0.0540 (0.00013) | 0.779 (0.0009) |
|  |  |  | 70 | -0.002 (0.0015) | 0.0549 (0.00013) | 0.786 (0.0009) |
|  |  |  | 80 | -0.002 (0.0015) | 0.0558 (0.00014) | 0.793 (0.0009) |
|  |  |  | 90 | -0.002 (0.0016) | 0.0573 (0.00014) | 0.804 (0.0009) |
|  |  | 0.7 | 0 | -0.000 (0.0008) | 0.0364 (0.00006) | 0.515 (0.0014) |
|  |  |  | 10 | -0.000 (0.0008) | 0.0374 (0.00007) | 0.542 (0.0014) |
|  |  |  | 20 | -0.000 (0.0009) | 0.0384 (0.00007) | 0.565 (0.0013) |
|  |  |  | 30 | -0.001 (0.0009) | 0.0397 (0.00008) | 0.592 (0.0014) |
|  |  |  | 40 | -0.002 (0.0010) | 0.0410 (0.00008) | 0.619 (0.0013) |
|  |  |  | 50 | -0.003 (0.0011) | 0.0427 (0.00009) | 0.649 (0.0012) |
|  |  |  | 60 | -0.005 (0.0012) | 0.0446 (0.00009) | 0.678 (0.0013) |
|  |  |  | 70 | -0.006 (0.0012) | 0.0470 (0.00011) | 0.709 (0.0013) |
|  |  |  | 80 | -0.008 (0.0014) | 0.0500 (0.00013) | 0.742 (0.0012) |
|  |  |  | 90 | -0.009 (0.0015) | 0.0533 (0.00015) | 0.772 (0.0012) |

# **Appendix S1 - Applied example**

## Assessment of the assumed relationship between variables and missing data in the applied example


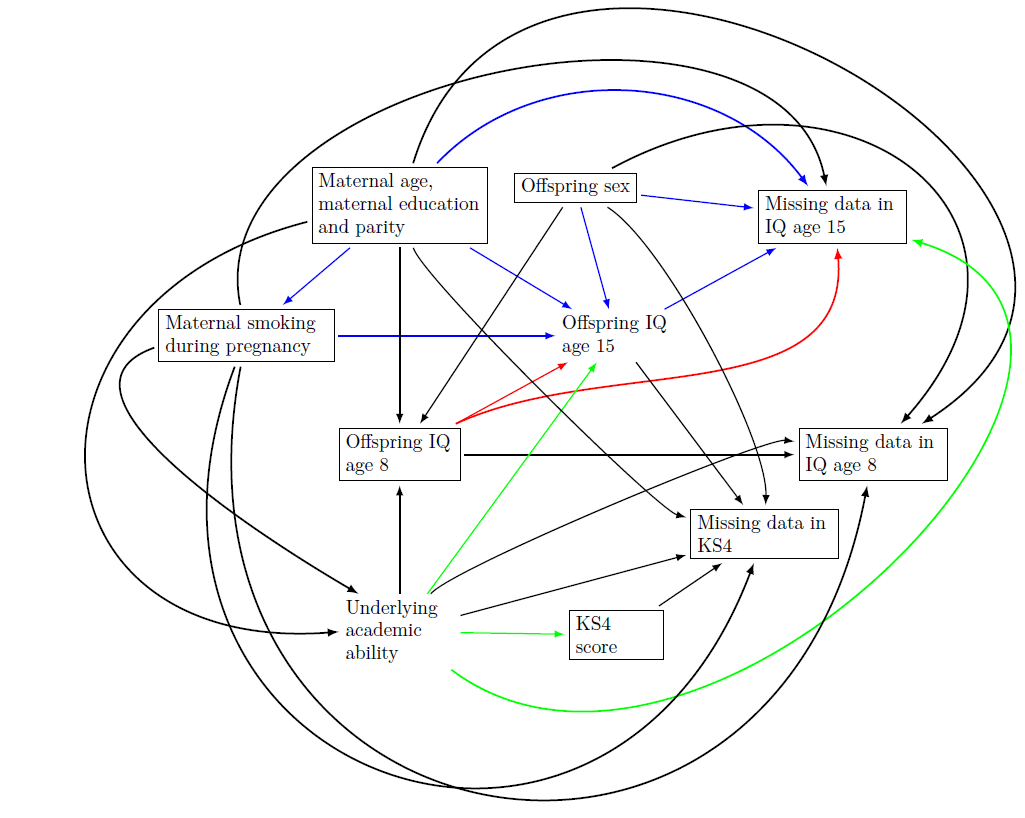


### **Figure S1**: Directed acyclic graph for the applied example. Lines in blue show relationships between variables that are part of the complete records analysis model.

Lines in red and green represent a biasing pathways that are closed through the inclusion of IQ at age 8 and KS4 score respectively in the imputation model.

Figure S1 shows a DAG of the assumed relationship between variables and missing data indicators in the applied example. The DAG has been simplified, which suffices for our purposes, and not all relationships are represented. Below we justify the assumed relationship between each node and detail some of the assumed parametric relationships that cannot be captured by DAGs (because DAGs are not parametric in nature).

### Covariates included in the analysis model

Confounding variables were recorded using maternal questionnaire during the pregnancy period. Maternal age, parity and education proxy for socioeconomic position which is associated with maternal smoking in pregnancy ^2,3^ and offspring IQ ^4-6^. Offspring sex may explain some of the variation in IQ ^7^, likely as a result of sex-differences in conscientiousness or school children’s ability to take cognitive tests rather than sex differences in average intelligence, though sex will not influence the exposure. As such we do not consider this a confounder. It can, however, be used to reduce standard error of the exposure coefficient.

### Auxiliary variables

Figure S1 shows that the auxiliary variable offspring IQ at age 8 is on the causal pathway between the exposure, maternal smoking during pregnancy, and the outcome, IQ at age 15. Inclusion of IQ at age 8 in the analysis model would therefore lead to estimation of a direct effect of exposure on outcome not via IQ at age 8. Thus we do not include IQ at age 8 in the analysis model, as we wish to estimate the total effect of exposure on outcome.

In contrast, KS4 scores are obtained after the measurement of the outcome at the age of 16. Adjustment for KS4 score in the analysis model would result in collider bias as the variable is a descendent of both the exposure and the outcome.

The auxiliary variables therefore have to act as proxies for missing outcome information within the imputation model ^8,9^ to ensure estimation of the effect we are interested in and to prevent collider bias. Inclusion of either IQ at age 8 or KS4 score in the imputation model will remove a portion of the relationship between the outcome and its own missing data indicator thereby reducing bias from missing data (as a result of blocking the open path in red along IQ at age 15 ← IQ at age 8 → missing data in IQ at age 15, or the path in green along IQ at age 15 ← underlying academic ability proxied for by KS4 scores at age 16 → missing data in IQ at age 15). It is important to note that inclusion of these auxiliary variables in the imputation model does not completely sever the relationship between the outcome and its missing data indicator as a result of the direct arrow from IQ at age 15 to missing data in IQ at age 15. Some bias from missing data will therefore remain and is unable to be removed without additional data or additional assumptions.

In Figure S1 we did not include an arrow from IQ at age 15 to missing data in IQ at age 8 as a later measurement cannot cause missing data in an earlier measurement. IQ at age 15 could plausibly cause missing KS4 scores, captured at age 16. Combined with the fact that we consider it likely that both auxiliary variables may be a cause of their own missingness, it is possible that including these auxiliary variables in the imputation model may create pathways from the outcome to the missing data indicator for the auxiliary variables, thereby introducing some bias. More pathways exist for KS4 score as an auxiliary than IQ at age 8, if IQ at age 15 were to cause missing data in KS4 score, though any bias may be smaller due to the lower proportion of missing data in KS4 score than IQ at age 8. We consider the amount of bias introduced by inclusion of the auxiliary variables to be less than the amount removed by closing pathways from IQ at age 15 to its own missing data indicator.

It has previously been shown that IQ and KS4 attainment are related in a non-linear way and that the relationship varies according to maternal education ^9^ – this cannot be represented within the DAG but needs to be considered when imputing values of IQ from KS4 attainment.

### Patterns of missing data and missing data mechanisms

Data in the outcome (IQ at age 15) were observed for 40% of participants and were observed in the auxiliary variables IQ at age 8 and KS4 score for 56% and 75% of participants respectively (see Table S1). The patterns of missing data in the outcome and each auxiliary variable are presented in Table S4. For 30% of participants, data in the outcome and both auxiliary variables were observed, while for approximately 10% of participants the outcome was observed with missing data in one or more of the auxiliary variables. The outcome was missing with at least one auxiliary variable observed for 46% of participants. In 41% of participants the outcome was missing but KS4 score was observed. In contrast, 20% of participants had missing outcome but IQ at age 8 observed. KS4 score can therefore be used to predict a greater proportion of missing values in the outcome than can IQ at age 8. Use of IQ at age 8 and KS4 scores as auxiliary variables in the imputation model will provide information on missing outcome data for an additional 5% of participants in comparison to using KS4 score alone. For 14% of participants there was missing data in the outcome and all auxiliary variables.

We attempt to provide evidence for the assumed relationships presented in Figure S1 by providing odds ratios (OR) in Table S5 for missing data in the outcome and each of the auxiliary variables according to each variable included in the applied example. It is important to note that the relationships between variables with missing data and missingness cannot be verified (and further that we have excluded participants with missing confounder data for simplicity of the example and so these results may not hold for ALSPAC more generally).

Table S5 shows that missing data in IQ variables (at age 8 and 15) was more likely among offspring with lower IQ and lower KS4 scores. Missing IQ was also more likely for offspring of mothers who were younger, had higher parity and had lower education levels. In contrast, KS4 scores were more likely to be missing among offspring with higher IQ at age 15 (but not age 8), and had mothers with higher levels of education. Parity and maternal age did not have strong evidence for an association with missing data in KS4 scores. Male sex and having a mother who smoked during pregnancy was associated with increased odds of missing data in IQ variables (at both ages, though the effect of sex was stronger at age 15 than age 8) and KS4 score. Maternal smoking during pregnancy was a strong predictor of missing data in IQ at age 15 (OR = 1.79; 95% CI = 1.62-1.96), missing data in IQ at age 8 (OR = 1.64; 95% CI = 1.50-1.79) and missing data in KS4 score (OR = 1.22; 95% CI = 1.10-1.34) after adjusting for maternal socioeconomic variables (education level, age and parity) suggesting that there may be a direct link from the exposure variable to missing data in the outcome and missing data in the auxiliary variables that cannot be accounted for by our measured confounding variables.

Prior work in ALSPAC has shown that socioeconomic position and sex predicted offspring participation in ALSPAC in late teenage years ^10^. The outcome (IQ at age 15) may therefore be more likely to be missing for those with lower IQ due to the relationship between lower socioeconomic position and lower IQ scores ^4-6^. In contrast linked educational data (capturing KS4 scores) could not be obtained for participants attending an independent or private school. These are schools where students are from a higher socioeconomic position, are more likely to have higher educational attainment and therefore have higher KS4 scores. The relationship between offspring sex and missing data in IQ at age 8 was smaller in magnitude than the relationship with missing data at age 15. This may be because participants are more likely to be brought to clinics for IQ testing by their parents. Missing data in KS4 scores was more likely among offspring who’s assigned sex at birth was male. This may be due to a larger proportion of males than females permanently excluded from school ^11^.

#### **Table S4**: Patterns of missing data in the outcome (IQ at age 15) and auxiliary variables (IQ at age 8 and KS4 score recorded at age 16).

|  | Pattern of missing data across variables | | |
| --- | --- | --- | --- |
| Percent with pattern | IQ age 15 | IQ age 8 | KS4 score |
| 30% | Observed | Observed | Observed |
| 26% | Missing | Missing | Observed |
| 15% | Missing | Observed | Observed |
| 14% | Missing | Missing | Missing |
| 5% | Observed | Observed | Missing |
| 5% | Missing | Observed | Missing |
| 4% | Observed | Missing | Observed |
| <1% | Observed | Missing | Missing |

N (%) observed: IQ at age 15 = 4,714 (40.0%); IQ age 8 = 6,567 (55.8%); KS4 score = 8,826 (74.9%)

#### **Table S5**: Odds ratios for missing data in the outcome and auxiliary variables according to the variables included in the applied example.

|  | OR (95% CI) for missing data in IQ at age 15 | OR (95% CI) for missing data in IQ at age 8 | OR (95% CI) for missing data in KS4 score |
| --- | --- | --- | --- |
| Offspring IQ at age 15 | - | 0.97 (0.96-0.98) | 1.02 (1.01-1.03) |
| Offspring IQ at age 8 | 0.98 (0.97-0.98) | - | 1.00 (1.00-1.01) |
| Offspring KS4 score | 0.99 (0.99-0.99) | 0.99 (0.99-0.99) | - |
| Maternal smoking during pregnancy | 2.35 (2.15-2.57) | 2.23 (2.06-2.41) | 1.13 (1.04-1.24) |
| Maternal age at birth | 0.92 (0.92-0.93) | 0.91 (0.90-0.91) | 1.01 (1.00-1.01) |
| Parity Categories |  |  |  |
| 0 | Ref | Ref | Ref |
| 1 | 1.19 (1.10-1.29) | 1.05 (0.97-1.14) | 0.85 (0.87-1.04) |
| 2+ | 1.65 (1.50-1.83) | 1.43 (1.30-1.57) | 1.04 (0.94-1.15) |
| Maternal highest education |  |  |  |
| O-level or lower | Ref | Ref | Ref |
| A-level/Degree or higher | 0.45 (0.42-0.49) | 0.44 (0.41-0.48) | 1.37 (1.26-1.49) |
| Offspring assigned sex at birth |  |  |  |
| Female | Ref | Ref | Ref |
| Male | 1.26 (1.18-1.35) | 1.12 (1.05-1.20) | 1.28 (1.19-1.37) |

## Fraction of missing information (FMI) results

The exposure coefficient had the largest FMI of all covariates in all MI models. Inclusion of auxiliary variables led to a reduction in the exposure FMI compared to MI excluding auxiliary variables. This shows that, despite the missing data in the auxiliary variables, their inclusion provided more information about the relationship between the exposure and missing outcome variable than excluding them from the imputation model. Inclusion of non-linear terms in the imputation model when KS4 score was used as an auxiliary led to an increase in the FMI (compare model iii to model v) suggesting that correct specification of the model does not necessarily reduce the FMI.

## Sensitivity analyses

### Distribution of missing data according to included vs. excluded participants

Supplementary Table S6 shows descriptives for included versus excluded participants with missing values in the confounders. Missing data in the outcome and auxiliary variables was substantially more likely when confounder variables were also missing.

#### **Table S6**: Distribution of observed and missing data in the sample with all confounders observed vs the sample with some missing confounder information.

Participants with missing values in the confounders were more likely to have a larger number of missing variables for the outcome, exposure and auxiliary variables.

|  | Total | All confounders observed (included) | Some confounders missing (excluded) |
| --- | --- | --- | --- |
|  | N=13,826 | N=11,780 | N=2,046 |
| Offspring IQ at age 15 |  |  |  |
| Observed | 4,983 (36.0%) | 4,714 (40.0%) | 269 (13.1%) |
| Missing | 8,843 (64.0%) | 7,066 (60.0%) | 1,777 (86.9%) |
| Offspring IQ at age 8 |  |  |  |
| Observed | 6,986 (50.5%) | 6,567 (55.7%) | 419 (20.5%) |
| Missing | 6,840 (49.5%) | 5,213 (44.3%) | 1,627 (79.5%) |
| Offspring KS4 score |  |  |  |
| Observed | 10,139 (73.3%) | 8,826 (74.9%) | 1,313 (64.2%) |
| Missing | 3,687 (26.7%) | 2,954 (25.1%) | 733 (35.8%) |
| Maternal smoking during pregnancy |  |  |  |
| Observed | 13,010 (94.1%) | 11,780 (100.0%) | 1,230 (60.1%) |
| Missing | 816 (5.9%) | 0 (0.0%) | 816 (39.9%) |
| Maternal age |  |  |  |
| Observed | 13,826 (100.0%) | 11,780 (100.0%) | 2,046 (100.0%) |
| Missing | 0 (0.0%) | 0 (0.0%) | 0 (0.0%) |
| Parity |  |  |  |
| Observed | 12,784 (92.5%) | 11,780 (100.0%) | 1,004 (49.1%) |
| Missing | 1,042 (7.5%) | 0 (0.0%) | 1,042 (50.9%) |
| Maternal education |  |  |  |
| Observed | 12,276 (88.8%) | 11,780 (100.0%) | 496 (24.2%) |
| Missing | 1,550 (11.2%) | 0 (0.0%) | 1,550 (75.8%) |

### Reducing the number of imputations used in the applied example

The number of imputations used in the applied example was larger than would feasibly be used in an applied study. We therefore reran analyses using 100 imputations. The results, which do not differ substantially from models using 1000 imputations, are presented in Supplementary Table S7.

#### **Table S7**: Model results for applied MI analyses using 100 imputations.

| Model | Exposure coefficient (s.e.) ^a^ | 95% CI | FMI for exposure coefficient |
| --- | --- | --- | --- |
| CRA | -0.87 (0.485) | -1.82, 0.08 | - |
| i) MI excluding auxiliaries | -0.81 (0.483) | -1.76, 0.15 | 0.70 |
| ii) MI with IQ at age 8 as an auxiliary | -1.13 (0.448) | -2.01,-0.24 | 0.65 |
| iii) MI with KS4 score as an auxiliary | -1.99 (0.431) | -2.83,-1.14 | 0.57 |
| iv) MI with IQ at age 8 and KS4 score as auxiliaries | -1.94 (0.428) | -2.78,-1.09 | 0.58 |
| v) MI with KS4 score cubed as an auxiliary (including multiplicative term ^b^) | -1.52 (0.467) | -2.44,-0.60 | 0.67 |
| vi) MI with IQ at age 8 and KS4 score cubed as auxiliaries (including multiplicative term ^b^) | -1.45 (0.407) | -2.25,-0.65 | 0.57 |

^a^ Mean difference in IQ at age 15 among offspring of maternal smokers during pregnancy compared to non-smokers during pregnancy
^b^ Multiplicative term between KS4 score cubed and maternal education

# **Appendix S2 - Further details on the fraction of missing information (FMI)**

For an estimate $\hat{\beta}$, which could be a regression coefficient that has been averaged across $m$ imputed datasets (i.e. $\hat{\beta}=(1/m\boldsymbol{)}\sum_{\boldsymbol{k=1}}^{\boldsymbol{m}} \hat{\beta}_{k}$) the FMI is given by

$$FMI\boldsymbol{=}\frac{\boldsymbol{B}}{\boldsymbol{(W+B)}}.$$

Here $\boldsymbol{W}$ is the within imputation variance of $\hat{\beta}$ and$\boldsymbol{B}$ is the between imputation variance of$\hat{\beta}$. These values are derived as follows. For$\hat{V}_{k}$, the squared standard error of$\hat{\beta}$in the$k^{th}$ imputed dataset, the within imputation variance across $m$imputations is given by

$$\boldsymbol{W}=(1/m\boldsymbol{)}\sum_{k=1}^{m} \hat{V}_{k}.$$

In other words, $\boldsymbol{W}$ is calculated as the average squared standard error of $\hat{\beta}$. The between imputation variance is given by

$$\boldsymbol{B=}\left( \frac{1}{m-1} \right)\sum_{k=1}^{m} (\hat{\beta}_{k}- \hat{\beta})^{2}.$$

This is the square of the standard deviation of the estimated regression coefficient in each imputed dataset relative to the mean value across all imputed datasets.

The total variance,$var(\hat{\beta})$ is equal to$\boldsymbol{W}+\left( 1+\frac{1}{m} \right)\boldsymbol{B}$. Hence for large numbers of imputations$m$*,* the FMI is the fraction of the total variance that is attributable to between imputation variance.

Being a fraction of the total variance, the FMI can take values between 0 and 1. Low values (i.e. close to 0) indicate that much of the “missing” information is in fact captured by other, more completely observed, variables. In the absence of any missing data the FMI is 0 because the between imputation variance,$\boldsymbol{B,}$ would be 0 since all imputation datasets would be identical. Of course, if we have no missing data we would not use multiple imputation and therefore would not estimate the FMI.

# **Appendix S3 - Simulation study**

## Justification for the number of simulation repetitions, sample size and distribution of simulated variables

We justify the number of simulations used based on the approach taken by Keogh and Morris and displayed in their supplement S5^1^. We are primarily interested in bias, the Monte Carlo standard error for which is

$$\text{MCSE}=\sqrt{\frac{\text{Var}}{\text{reps}}.}$$

Assuming that the variance of bias for any given model is 0.1, and aiming for an MCSE of 0.01 on estimated bias would require 1000 simulation repetitions as was used in the present study.

We selected the sample size of the simulations to be small enough to feasibly repeat over this many repetitions. This sample size is small compared to many real world studies and yet larger compared to many others. Exploration of the impact of different sample sizes is left for future work.

In our selection of the distribution of the variables in the simulation study we used a multivariate normal distribution with means $\bar{Y}=6$, $\bar{X}=-3$ and $\bar{Z}=2$ and variance 1. The means were chosen arbitrarily but selected to be non-zero as prior experience suggests using zero means can lead to unbiased estimates in situations in which this would generally not be the case. The use of multivariate normal distribution, as opposed to simulating variables using structural equation models, ensured that Z and X provided independent information on Y. This was an ideal scenario for the simulation study but unlikely in real world data.

## Pattens of missing data

The patterns of missing data in Y and Z averaged over simulations are presented in Figure S2. Observations where Y and Z are missing are presented as solid lines, where Y only is missing are presented as dashed lines and where Z only is missing are presented as dotted lines.

For auxiliary missingness mechanism 1 (missingness in Z not caused by any other variable) in all outcome missingness mechanisms (plots A, D and G) and for all auxiliary missingness mechanisms in outcome missingness mechanism 3 (missingness in Y caused by X; plots G-I) the correlation of Z and Y did not influence the missing data pattern. Further, for these plots the proportion with Z and Y missing is equal to the proportion missing Z only resulting in the solid and dotted lines being overlayed. This shows that the for half of the observations missing Z, Y was also missing.

For outcome missingness mechanism 1 (missingness in Y caused by X and Z, plots A-C), the correlation of Z and Y did not influence the missing data pattern but for auxiliary missingness mechanism 2 (missingness in Z caused by Z) and 3 (missingness in Z caused by W), the proportion with missing Y was more than half among those missing Z as indicated by the solid line being higher than the dotted line. This distribution changed as the proportion missing Z increased.

Finally, for outcome missingness mechanism 2 (missingness in Y caused by Y) under auxiliary missingness mechanism 2 and 3 (plots E and F) the missing data pattern did depend on the correlation of Y and Z. For these two combinations of missing data mechanisms the distribution of missing Y was also more than half among those missing Z (indicated by the solid line being higher than the dotted line). This proportion changed as the proportion missing Z increased.

## Standard error (SE) results

The SE for the exposure coefficient for all outcome and auxiliary missing data mechanism combinations are presented in Figure S3. The SE of the CRA depended on the outcome missingness mechanism and was further dependent on the correlation of Z and Y in outcome missingness mechanism 1 (plots A-C).

For outcome missingness mechanism 1 low correlations between Z and Y lead to higher SEs in MI models including auxiliary variables than in CRA under all missing auxiliary mechanisms. As the proportion of missing data in Z increased, the SE in MI models including Z in the imputation model approached the SE of CRA. This shows that when the auxiliary variable causes missing data in the variable to be imputed (here, the outcome), low correlation between the auxiliary and the variable to be imputed can reduce efficiency, even when there is no missing data in the auxiliary. Where the auxiliary variable is not a predictor of missingness in the outcome (missing outcome mechanisms 2 and 3) weak correlations between the auxiliary and the variable to be imputed do not increase the SE (thereby reducing efficiency) beyond that of CRA.

For all missingness mechanism combinations (with the exception of outcome mechanism 2 combined with auxiliary mechanism 2, plot E), increasing proportions of missing auxiliary data led the SE in MI models to approach the SE of CRA. For plot E with high correlation between Z and Y the SE of MI models reduced as the proportion missing Z increased to 30%, then the SE began to approach that of CRA as the proportion missing Z increased further. This unusual finding may be to do with the unique pattern of missing data as displayed in Figure S2E. It should be noted that for some combinations, at extreme proportions of missing auxiliary data (90%) the SE became greater than the SE of CRA despite initially reducing the SE (as in plot C and F).

## Fraction of missing information (FMI) results

We present the FMI for the exposure coefficient for all outcome and auxiliary missing data mechanism combinations in Figure S4. The FMI of MI models excluding auxiliary variables (horizontal dashed line in each plot) was largest for data simulated under outcome missingness mechanism 3 and smallest under mechanism 1. For all missing outcome mechanisms, lower correlation between Z and Y (reflecting a weaker ability of Z to predict Y) led to higher FMI in MI models including auxiliary variables with no missing auxiliary data. Under outcome mechanism 1 (Figure S4 A-C) inclusion of complete auxiliaries with low correlation between Z and Y (0.3 or 0.1) led to FMI values that were greater than MI models excluding auxiliary variables. When auxiliary variables were missing under auxiliary mechanisms 1 and 2, as the proportion of missing data in the auxiliary increased, the FMI of models with different correlations between Z and Y all converged to the value obtained in models excluding auxiliary variables. For missing auxiliary mechanism 3, the FMI of all MI models including auxiliary variables converged to a value greater than that observed in MI models excluding auxiliary variables. The behaviour of the FMI with increasing proportion of missing auxiliary data was similar between outcome mechanism 2 (Figure S4 D-F) and outcome mechanism 3 (Figure S4 G-I). In both cases, for all missing auxiliary mechanisms, low correlation between Z and Y in MI including auxiliary variables led to FMI values closer to the FMI of MI excluding auxiliary variables at all proportions of missing data. As the proportion of missing data in the auxiliary variable increased the FMI of models including auxiliary variables converged to the value of MI models excluding auxiliary variables.


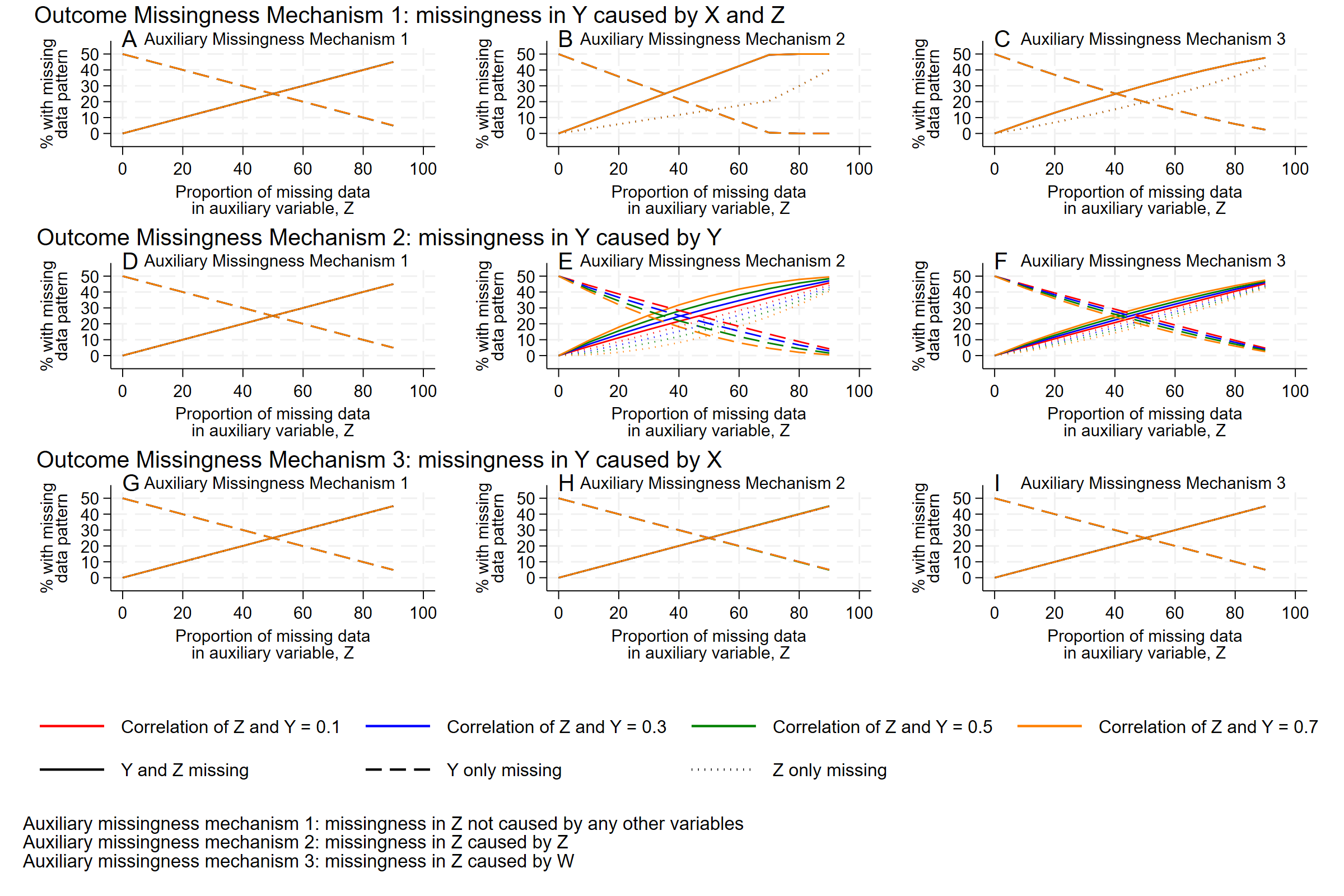


### **Figure S2**: Plots of the **mean proportion with each missing data pattern** (Y and Z missing, Y only missing, Z only missing) across simulations against the proportion of missing data in the auxiliary variable, Z, for each level of correlation between the auxiliary, and outcome, Y.

Monte Carlo 95% confidence intervals were omitted to improve clarity of the figure but were narrow in all plots.


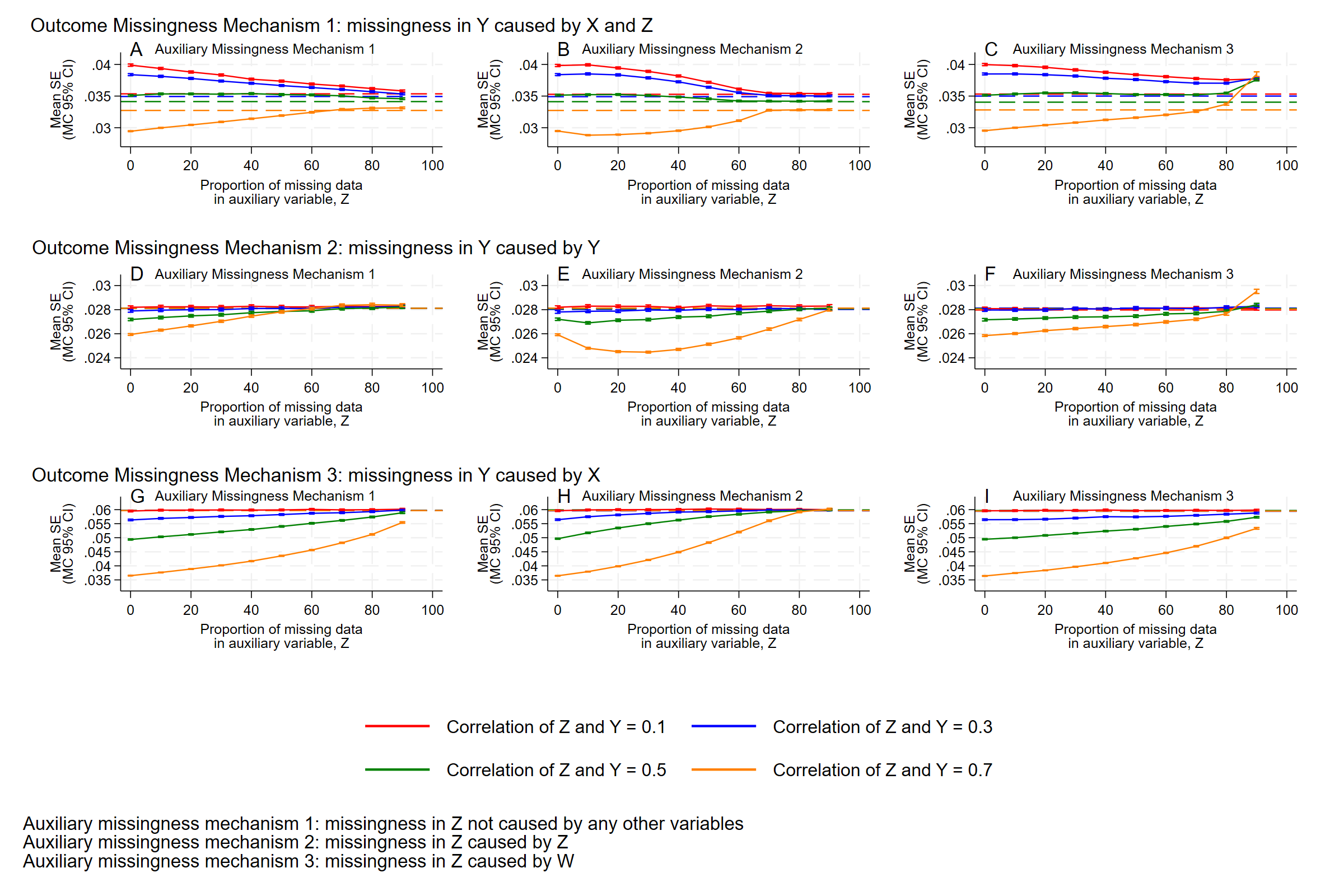


### **Figure S3**: Plots of **mean SE** of the effect estimate of the exposure, X, across simulations against the proportion of missing data in the auxiliary variable, Z, for each level of correlation between the auxiliary, and outcome, Y.

Solid lines correspond to the SE of MI models including auxiliary variables while dashed lines correspond to the SE in complete records analysis. CI = confidence interval, MC = Monte Carlo, SE = Standard error


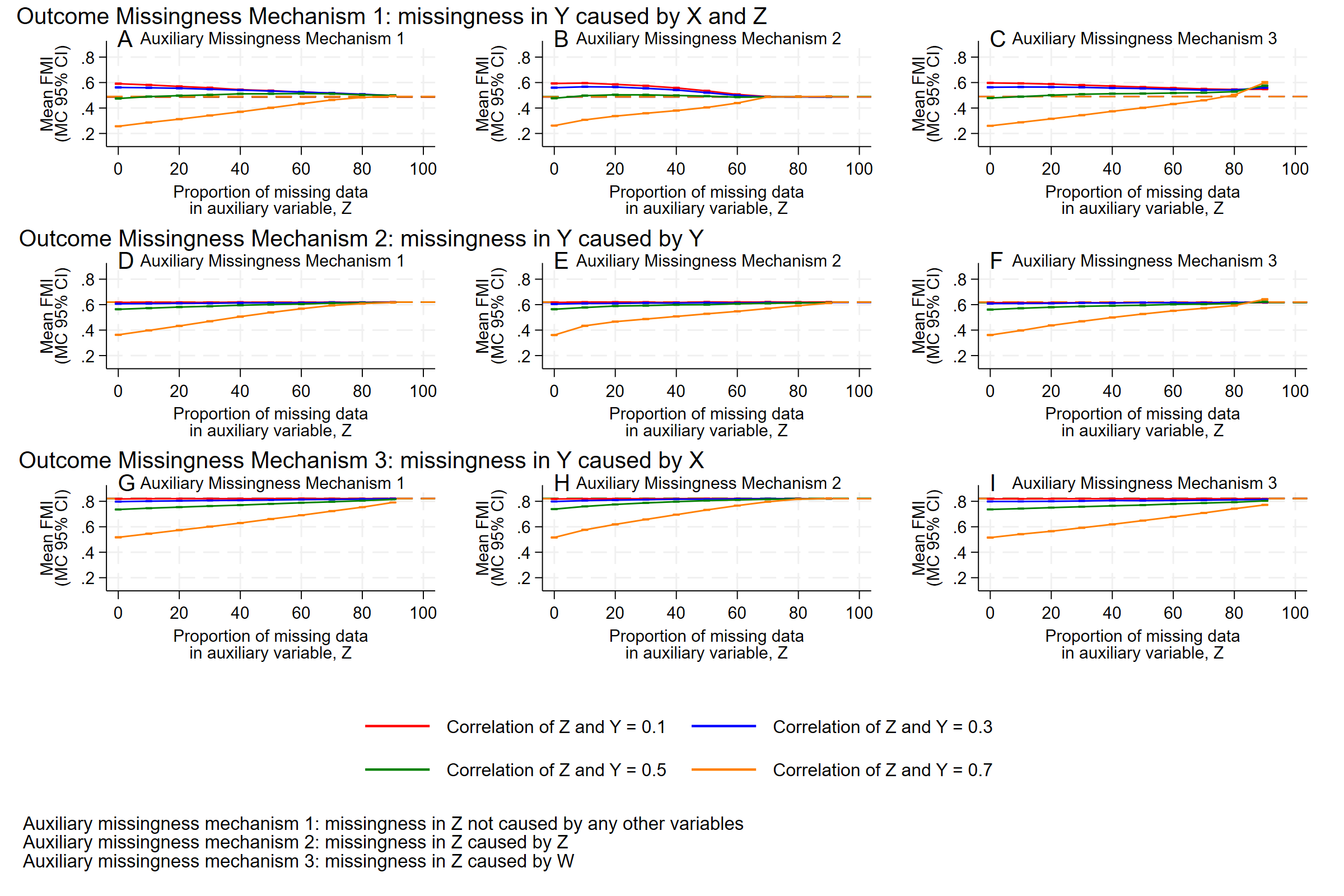


### **Figure S4**: Plots of **mean FMI** of the effect estimate of the exposure, X, across simulations against the proportion of missing data in the auxiliary variable, Z, for each level of correlation between the auxiliary, and outcome, Y.

Solid lines correspond to FMI of MI including auxiliary variables while dashed lines correspond to FMI of MI excluding auxiliary variables. FMI = fraction of missing information, CI = confidence interval, MC = Monte Carlo

## Sensitivity analyses

### Methods

In sensitivity analyses we explored the impact of i) the proportion of missing data in the outcome and ii) the strength of association between X and Y relative to Z and Y. We therefore repeated the simulation study using i) 75% missing data in the outcome and ii) a correlation between X and Y of 0.2. Our sensitivity analyses were conducted under missing auxiliary mechanism 3 (missingness in the auxiliary caused by another variable, W) for missing outcome mechanisms 1 and 2, and under missing auxiliary mechanism 2 (missingness in the auxiliary caused by the auxiliary itself) for missing outcome mechanism 3. The mechanisms explored were selected based on the results of the main simulation results as the most important scenarios to understand. For outcome mechanism 1 and 2 we chose to assess sensitivity under the scenario where we can break the dependency between the outcome and missing data in the auxiliary as these outcome mechanisms are biased under a CRA and we want to understand how the change in parameters influenced our ability to **reduce** bias. For outcome mechanism 3 there was no bias in CRA but there was under some MI models, and so we wanted to understand how modification of the parameters influenced the quantity of bias **introduced**.

We additionally explored the impact of excluding W from the imputation model for Z under missing auxiliary mechanism 3 for all missing outcome mechanisms. This scenario is an example where inclusion of the auxiliary, Z, introduces a dependency between the outcome and a missing data indicator (missing data in Z) as Z was missing dependent on W.

### Results

#### Bias for sensitivity analysis i) and ii)

The bias under CRA and MI for sensitivity analyses assessing the impact of i) the proportion of missing data in the outcome and ii) the strength of association between X and Y relative to Z and Y are presented in Tables S8 and S9 and in Figure S5. Under outcome missingness mechanism 1 and 2, bias in CRA (and equivalently bias in MI models excluding auxiliary variables) was increased when the proportion of missing data in the outcome variable was increased from 50% to 75%. In contrast, when the correlation between X and Y was reduced from 0.6 to 0.2, the bias in CRA remained approximately equivalent under missing outcome mechanism 1 but was reduced under missing outcome mechanism 2. Bias was approximately equal to zero for all CRA models under missing outcome mechanism 3.

Under outcome mechanism 1 (missingness in the outcome caused by the exposure and auxiliary variable), bias relative to CRA remained approximately the same as in the main analyses when the proportion of missing outcome was increased (compare Figure S5A to Figure 2C) and when the correlation between X and Y was reduced (compare Figure S5D to Figure 2C), though the reduction in relative bias for the largest correlation between Z and Y was less pronounced in sensitivity analysis ii). This suggests that MI is consistent in its ability to reduce bias when the auxiliary variable reduces the dependence between the outcome and missingness in the outcome without introducing a dependency with missingness in the auxiliary variable, irrespective of changes in the proportion of missing outcome data or reductions in the strength of association between exposure and outcome.

For outcome mechanism 2 (missingness in the outcome is caused by the outcome itself), bias relative to CRA increased when the proportion of missing outcome data was increased from 50 to 75% (compare Figure S5B to Figure 2F) and when the correlation between X and Y was reduced from 0.6 to 0.2 (compare Figure S5E to Figure 2F). This was true for all proportions of missing auxiliary data and all strengths of correlation between Y and Z. This suggests that when we use auxiliary variables to act as proxy variables for the outcome, the ability of MI to reduce bias relative to CRA is influenced by other factors, even when the auxiliary is independent of its own missingness given complete W.

For outcome mechanism 3 (missingness in the outcome is caused by the exposure variable only), where there was (close to) zero bias in the CRA, the bias relative to CRA in sensitivity analysis for MI using auxiliary variables missing dependent on their own value (auxiliary mechanism 2) was many times larger than that of the primary analyses (approx. 12000% vs 4300%) when the proportion of missing outcome data was increased to 75%. The reason for this was a slightly smaller amount of bias in CRA for sensitivity analyses (0.001) than in primary analyses (-0.002) which inflated relative estimates. This highlights the importance of looking at absolute bias for outcome mechanism 3 combined with auxiliary mechanism 2. In terms of absolute bias increasing the proportion of missing data in the outcome to 75% actually decreased the amount of bias from -0.101 in primary analyses to -0.074 in sensitivity analyses. The maximum quantity of bias remained at around 50% missing data in the auxiliary variable, even when the proportion of missing data in the outcome was increased to be greater than 50%, suggesting that this is a pattern not introduced as a result of our simulation design choice to use 50% missing outcome data.

#### FMI for sensitivity analyses i) and ii)

Results for the FMI for sensitivity analyses i) and ii) are presented in Tables S8 and S9 and in Figure S6. Under missing outcome mechanism 1 (missingness in the outcome caused by the exposure and auxiliary variable), in primary and sensitivity analyses MI models with large proportions of auxiliary data missing under auxiliary mechanism 3 led to FMI values that were larger than values from models in which auxiliary variables were excluded. The proportion of missing auxiliary data at which including the auxiliary variable made the FMI worse than if the auxiliary was excluded, varied according to factors such as how much missing data in the outcome there was (compare Figure S6A to Figure S4C) and the strength of association between exposure and outcome (compare Figure S6D to Figure S4C). The FMI increased for all correlations between Z and Y and all proportions of missing auxiliary variable when the proportion of the outcome that was missing increased from 50% to 75%, highlighting the impact of the proportion of missing data in the analysis model on the FMI, irrespective of the amount of missing data in the auxiliary variable. When the correlation of X and Y was reduced to 0.2, the reduction in FMI at low proportions of missing auxiliary data with higher correlations between Z and Y was smaller, suggesting that there is a reduced ability for the auxiliary to retain missing information on the exposure-outcome effect estimate when this effect is truly smaller.

When the outcome was missing dependent on its own value (missing outcome mechanism 2) in primary analyses the FMI was approximately equal to the MI model excluding auxiliary variables for weaker correlations (≤0.3) between auxiliary and outcome (see Figure S4F). For stronger correlations (≥0.5) the FMI was lower than the MI model excluding auxiliary variables while the proportion of missing data in the auxiliary variable remained lower than 50%. Sensitivity analysis showed that a similar pattern was found when the proportion of missing data in the outcome increased to 75% (compare Figure S6B to Figure S4F) or when the correlation between exposure and outcome was reduced (compare Figure S6E to Figure S4F).

Under outcome mechanism 3 where the missingness in the outcome was caused by the exposure, the FMI behaved in a comparable manner to mechanism 2, though the maximum value of the FMI was greater than that observed under mechanism 1 or 2 (compare Plot C to A and B and Plot F to D and E in Figure S6). The FMI was greater in sensitivity analyses where the proportion of missing outcome was increased from 50% to 75% (compare Figure S6C to Figure S4H) but remained at a similar value to primary analyses when the correlation between X and Y was reduced (compare Figure S6F to Figure S4H).

#### **Table S8**: Sensitivity analysis – average bias, standard error and fraction of missing information across simulations in complete records analysis and MI models excluding auxiliary variables for sensitivity analysis i) and ii).

| Missing auxiliary mechanism | Missing outcome mechanism | Sensitivity analysis | Correlation between  Z and Y | CRA models | | MI models excluding auxiliaries | | |
| --- | --- | --- | --- | --- | --- | --- | --- | --- |
|  |  |  |  | Bias (MCSE) | SE (MCSE) | Bias (MCSE) | SE (MCSE) | FMI(MCSE) |
| Mechanism 3 – missingness in auxiliary, Z, caused by another variable, W | Mechanism 1 – missingness in outcome, Y, is caused by the exposure | 1 – more missing outcome (75%) | 0.1 | -0.060 (0.0011) | 0.0452 (0.00010) | -0.060 (0.0011) | 0.0454 (0.00012) | 0.688 (0.0013) |
|  |  |  | 0.3 | -0.177 (0.0012) | 0.0448 (0.00010) | -0.177 (0.0012) | 0.0451 (0.00012) | 0.687 (0.0014) |
|  |  |  | 0.5 | -0.295 (0.0013) | 0.0438 (0.00010) | -0.295 (0.0013) | 0.0441 (0.00012) | 0.688 (0.0013) |
|  |  |  | 0.7 | -0.414 (0.0012) | 0.0424 (0.00010) | -0.414 (0.0012) | 0.0429 (0.00012) | 0.690 (0.0014) |
|  |  | 2 – smaller X-Y correlation (0.2) | 0.1 | -0.046 (0.0010) | 0.0432 (0.00006) | -0.046 (0.0010) | 0.0434 (0.00008) | 0.490 (0.0015) |
|  |  |  | 0.3 | -0.138 (0.0009) | 0.0431 (0.00007) | -0.138 (0.0009) | 0.0432 (0.00008) | 0.490 (0.0015) |
|  |  |  | 0.5 | -0.230 (0.0010) | 0.0423 (0.00007) | -0.230 (0.0010) | 0.0424 (0.00008) | 0.487 (0.0015) |
|  |  |  | 0.7 | -0.321 (0.0010) | 0.0413 (0.00007) | -0.321 (0.0010) | 0.0415 (0.00008) | 0.490 (0.0014) |
|  | Mechanism 2 – missingness in outcome, Y, is caused by the outcome itself | 1 – more missing outcome (75%) | 0.1 | -0.400 (0.0012) | 0.0345 (0.00008) | -0.400 (0.0012) | 0.0348 (0.00011) | 0.823 (0.0009) |
|  |  |  | 0.3 | -0.399 (0.0012) | 0.0345 (0.00009) | -0.398 (0.0012) | 0.0348 (0.00011) | 0.821 (0.0009) |
|  |  |  | 0.5 | -0.402 (0.0011) | 0.0344 (0.00008) | -0.402 (0.0011) | 0.0347 (0.00011) | 0.822 (0.0009) |
|  |  |  | 0.7 | -0.401 (0.0011) | 0.0343 (0.00008) | -0.401 (0.0011) | 0.0346 (0.00011) | 0.822 (0.0009) |
|  |  | 2 – smaller X-Y correlation (0.2) | 0.1 | -0.126 (0.0010) | 0.0272 (0.00004) | -0.126 (0.0010) | 0.0273 (0.00006) | 0.516 (0.0014) |
|  |  |  | 0.3 | -0.126 (0.0010) | 0.0272 (0.00005) | -0.126 (0.0010) | 0.0273 (0.00005) | 0.519 (0.0014) |
|  |  |  | 0.5 | -0.127 (0.0009) | 0.0273 (0.00005) | -0.127 (0.0010) | 0.0275 (0.00006) | 0.520 (0.0014) |
|  |  |  | 0.7 | -0.126 (0.0010) | 0.0272 (0.00004) | -0.126 (0.0010) | 0.0273 (0.00005) | 0.517 (0.0014) |
| Mechanism 2 – missingness in auxiliary, Z, caused by the auxiliary itself | Mechanism 3 – missingness in outcome, Y, is caused by the exposure variable, X, only | 1 – more missing outcome (75%) | 0.1 | 0.005 (0.0032) | 0.1045 (0.00027) | 0.004 (0.0032) | 0.1054 (0.00036) | 0.942 (0.0003) |
|  |  |  | 0.3 | 0.004 (0.0032) | 0.1042 (0.00026) | 0.004 (0.0033) | 0.1052 (0.00034) | 0.942 (0.0003) |
|  |  |  | 0.5 | 0.003 (0.0032) | 0.1042 (0.00027) | 0.002 (0.0032) | 0.1049 (0.00036) | 0.942 (0.0003) |
|  |  |  | 0.7 | 0.001 (0.0032) | 0.1038 (0.00026) | 0.001 (0.0032) | 0.1045 (0.00035) | 0.942 (0.0003) |
|  |  | 2 – smaller X-Y correlation (0.2) | 0.1 | -0.000 (0.0022) | 0.0730 (0.00012) | 0.000 (0.0022) | 0.0736 (0.00018) | 0.823 (0.0008) |
|  |  |  | 0.3 | 0.003 (0.0021) | 0.0729 (0.00012) | 0.003 (0.0021) | 0.0737 (0.00018) | 0.823 (0.0008) |
|  |  |  | 0.5 | -0.000 (0.0021) | 0.0732 (0.00012) | -0.000 (0.0021) | 0.0735 (0.00018) | 0.822 (0.0008) |
|  |  |  | 0.7 | -0.002 (0.0021) | 0.0731 (0.00012) | -0.002 (0.0021) | 0.0737 (0.00019) | 0.822 (0.0008) |

CRA = Complete Records Analysis; FMI = Fraction of Missing Information; MCSE = Monte Carlo Standard Error; MI = Multiple imputation; SE = Standard Error

#### **Table S9**: Sensitivity analysis – average bias, standard error and fraction of missing information across simulations in MI models including auxiliary variables for sensitivity analysis i) and ii).

| Missing auxiliary mechanism | Missing outcome mechanism | Sensitivity analysis | Correlation between  Z and Y | Proportion of missing auxiliary data | MI models including auxiliaries | | |
| --- | --- | --- | --- | --- | --- | --- | --- |
|  |  |  |  |  | Bias (MCSE) | SE (MCSE) | FMI(MCSE) |
| Mechanism 3 – missingness in auxiliary, Z, caused by another variable, W | Mechanism 1 – missingness in outcome, Y, is caused by the exposure | 1 – more missing outcome (75%) | 0.1 | 0 | -0.001 (0.0015) | 0.0563 (0.00017) | 0.794 (0.0011) |
|  |  |  |  | 10 | -0.003 (0.0015) | 0.0564 (0.00018) | 0.795 (0.0011) |
|  |  |  |  | 20 | -0.007 (0.0015) | 0.0562 (0.00018) | 0.793 (0.0012) |
|  |  |  |  | 30 | -0.012 (0.0015) | 0.0555 (0.00018) | 0.788 (0.0012) |
|  |  |  |  | 40 | -0.017 (0.0015) | 0.0546 (0.00017) | 0.782 (0.0011) |
|  |  |  |  | 50 | -0.023 (0.0014) | 0.0535 (0.00016) | 0.773 (0.0012) |
|  |  |  |  | 60 | -0.029 (0.0014) | 0.0525 (0.00016) | 0.764 (0.0012) |
|  |  |  |  | 70 | -0.034 (0.0013) | 0.0515 (0.00016) | 0.755 (0.0013) |
|  |  |  |  | 80 | -0.040 (0.0013) | 0.0506 (0.00015) | 0.747 (0.0013) |
|  |  |  |  | 90 | -0.046 (0.0013) | 0.0501 (0.00015) | 0.742 (0.0013) |
|  |  |  | 0.3 | 0 | 0.000 (0.0015) | 0.0534 (0.00016) | 0.770 (0.0012) |
|  |  |  |  | 10 | -0.005 (0.0015) | 0.0537 (0.00017) | 0.772 (0.0012) |
|  |  |  |  | 20 | -0.015 (0.0015) | 0.0537 (0.00017) | 0.773 (0.0012) |
|  |  |  |  | 30 | -0.029 (0.0015) | 0.0531 (0.00016) | 0.771 (0.0012) |
|  |  |  |  | 40 | -0.044 (0.0015) | 0.0530 (0.00016) | 0.771 (0.0012) |
|  |  |  |  | 50 | -0.062 (0.0015) | 0.0520 (0.00016) | 0.764 (0.0013) |
|  |  |  |  | 60 | -0.079 (0.0014) | 0.0514 (0.00015) | 0.759 (0.0012) |
|  |  |  |  | 70 | -0.097 (0.0014) | 0.0504 (0.00015) | 0.750 (0.0013) |
|  |  |  |  | 80 | -0.115 (0.0013) | 0.0497 (0.00015) | 0.743 (0.0013) |
|  |  |  |  | 90 | -0.134 (0.0013) | 0.0498 (0.00015) | 0.743 (0.0013) |
|  |  |  | 0.5 | 0 | -0.001 (0.0013) | 0.0469 (0.00013) | 0.703 (0.0014) |
|  |  |  |  | 10 | -0.006 (0.0013) | 0.0476 (0.00014) | 0.712 (0.0015) |
|  |  |  |  | 20 | -0.019 (0.0013) | 0.0480 (0.00014) | 0.720 (0.0014) |
|  |  |  |  | 30 | -0.037 (0.0013) | 0.0484 (0.00015) | 0.729 (0.0014) |
|  |  |  |  | 40 | -0.061 (0.0014) | 0.0486 (0.00015) | 0.736 (0.0014) |
|  |  |  |  | 50 | -0.089 (0.0014) | 0.0487 (0.00014) | 0.741 (0.0013) |
|  |  |  |  | 60 | -0.120 (0.0014) | 0.0485 (0.00015) | 0.743 (0.0013) |
|  |  |  |  | 70 | -0.152 (0.0014) | 0.0484 (0.00015) | 0.743 (0.0013) |
|  |  |  |  | 80 | -0.185 (0.0013) | 0.0479 (0.00014) | 0.739 (0.0013) |
|  |  |  |  | 90 | -0.220 (0.0013) | 0.0494 (0.00016) | 0.751 (0.0013) |
|  |  |  | 0.7 | 0 | -0.000 (0.0008) | 0.0353 (0.00007) | 0.482 (0.0018) |
|  |  |  |  | 10 | -0.003 (0.0008) | 0.0360 (0.00007) | 0.503 (0.0017) |
|  |  |  |  | 20 | -0.010 (0.0009) | 0.0368 (0.00008) | 0.525 (0.0018) |
|  |  |  |  | 30 | -0.021 (0.0009) | 0.0378 (0.00009) | 0.556 (0.0018) |
|  |  |  |  | 40 | -0.038 (0.0010) | 0.0390 (0.00009) | 0.588 (0.0018) |
|  |  |  |  | 50 | -0.064 (0.0011) | 0.0404 (0.00010) | 0.625 (0.0018) |
|  |  |  |  | 60 | -0.101 (0.0012) | 0.0420 (0.00012) | 0.663 (0.0017) |
|  |  |  |  | 70 | -0.152 (0.0013) | 0.0437 (0.00014) | 0.699 (0.0017) |
|  |  |  |  | 80 | -0.214 (0.0013) | 0.0450 (0.00014) | 0.724 (0.0015) |
|  |  |  |  | 90 | -0.286 (0.0014) | 0.0487 (0.00019) | 0.761 (0.0015) |
|  |  | 2 – smaller X-Y correlation (0.2) | 0.1 | 0 | 0.001 (0.0012) | 0.0489 (0.00011) | 0.595 (0.0014) |
|  |  |  |  | 10 | -0.001 (0.0012) | 0.0489 (0.00010) | 0.596 (0.0015) |
|  |  |  |  | 20 | -0.005 (0.0012) | 0.0485 (0.00010) | 0.590 (0.0015) |
|  |  |  |  | 30 | -0.009 (0.0012) | 0.0480 (0.00010) | 0.580 (0.0015) |
|  |  |  |  | 40 | -0.013 (0.0012) | 0.0475 (0.00010) | 0.572 (0.0015) |
|  |  |  |  | 50 | -0.018 (0.0011) | 0.0471 (0.00010) | 0.564 (0.0015) |
|  |  |  |  | 60 | -0.022 (0.0011) | 0.0465 (0.00009) | 0.555 (0.0014) |
|  |  |  |  | 70 | -0.026 (0.0011) | 0.0461 (0.00010) | 0.547 (0.0016) |
|  |  |  |  | 80 | -0.030 (0.0011) | 0.0460 (0.00009) | 0.544 (0.0015) |
|  |  |  |  | 90 | -0.034 (0.0011) | 0.0461 (0.00009) | 0.546 (0.0015) |
|  |  |  | 0.3 | 0 | -0.000 (0.0011) | 0.0479 (0.00010) | 0.577 (0.0014) |
|  |  |  |  | 10 | -0.007 (0.0011) | 0.0479 (0.00010) | 0.577 (0.0014) |
|  |  |  |  | 20 | -0.017 (0.0011) | 0.0477 (0.00010) | 0.575 (0.0015) |
|  |  |  |  | 30 | -0.029 (0.0011) | 0.0475 (0.00010) | 0.572 (0.0015) |
|  |  |  |  | 40 | -0.042 (0.0011) | 0.0469 (0.00010) | 0.565 (0.0015) |
|  |  |  |  | 50 | -0.055 (0.0011) | 0.0465 (0.00010) | 0.558 (0.0015) |
|  |  |  |  | 60 | -0.067 (0.0010) | 0.0461 (0.00010) | 0.550 (0.0015) |
|  |  |  |  | 70 | -0.079 (0.0010) | 0.0459 (0.00010) | 0.546 (0.0015) |
|  |  |  |  | 80 | -0.092 (0.0010) | 0.0457 (0.00009) | 0.544 (0.0015) |
|  |  |  |  | 90 | -0.104 (0.0010) | 0.0463 (0.00010) | 0.554 (0.0015) |
|  |  |  | 0.5 | 0 | 0.001 (0.0010) | 0.0451 (0.00009) | 0.522 (0.0016) |
|  |  |  |  | 10 | -0.009 (0.0010) | 0.0453 (0.00009) | 0.530 (0.0016) |
|  |  |  |  | 20 | -0.023 (0.0010) | 0.0453 (0.00009) | 0.534 (0.0016) |
|  |  |  |  | 30 | -0.042 (0.0011) | 0.0453 (0.00009) | 0.538 (0.0016) |
|  |  |  |  | 40 | -0.063 (0.0011) | 0.0450 (0.00009) | 0.537 (0.0015) |
|  |  |  |  | 50 | -0.084 (0.0010) | 0.0448 (0.00009) | 0.536 (0.0016) |
|  |  |  |  | 60 | -0.105 (0.0010) | 0.0445 (0.00009) | 0.533 (0.0016) |
|  |  |  |  | 70 | -0.126 (0.0010) | 0.0442 (0.00009) | 0.528 (0.0015) |
|  |  |  |  | 80 | -0.148 (0.0010) | 0.0444 (0.00009) | 0.534 (0.0015) |
|  |  |  |  | 90 | -0.171 (0.0011) | 0.0463 (0.00011) | 0.568 (0.0017) |
|  |  |  | 0.7 | 0 | 0.003 (0.0009) | 0.0409 (0.00007) | 0.422 (0.0015) |
|  |  |  |  | 10 | -0.007 (0.0009) | 0.0414 (0.00007) | 0.439 (0.0015) |
|  |  |  |  | 20 | -0.023 (0.0009) | 0.0417 (0.00008) | 0.455 (0.0016) |
|  |  |  |  | 30 | -0.045 (0.0009) | 0.0419 (0.00008) | 0.469 (0.0016) |
|  |  |  |  | 40 | -0.070 (0.0010) | 0.0419 (0.00008) | 0.480 (0.0015) |
|  |  |  |  | 50 | -0.098 (0.0010) | 0.0419 (0.00008) | 0.488 (0.0015) |
|  |  |  |  | 60 | -0.129 (0.0010) | 0.0421 (0.00008) | 0.498 (0.0015) |
|  |  |  |  | 70 | -0.161 (0.0010) | 0.0421 (0.00009) | 0.504 (0.0016) |
|  |  |  |  | 80 | -0.196 (0.0010) | 0.0427 (0.00009) | 0.519 (0.0016) |
|  |  |  |  | 90 | -0.235 (0.0012) | 0.0461 (0.00013) | 0.582 (0.0020) |
|  | Mechanism 2 – missingness in outcome, Y, is caused by the outcome itself | 1 – more missing outcome (75%) | 0.1 | 0 | -0.399 (0.0012) | 0.0348 (0.00011) | 0.822 (0.0008) |
|  |  |  |  | 10 | -0.398 (0.0012) | 0.0351 (0.00011) | 0.824 (0.0009) |
|  |  |  |  | 20 | -0.399 (0.0012) | 0.0348 (0.00011) | 0.821 (0.0009) |
|  |  |  |  | 30 | -0.399 (0.0012) | 0.0349 (0.00011) | 0.822 (0.0009) |
|  |  |  |  | 40 | -0.399 (0.0012) | 0.0350 (0.00011) | 0.824 (0.0009) |
|  |  |  |  | 50 | -0.399 (0.0012) | 0.0348 (0.00011) | 0.822 (0.0009) |
|  |  |  |  | 60 | -0.399 (0.0012) | 0.0350 (0.00011) | 0.824 (0.0009) |
|  |  |  |  | 70 | -0.400 (0.0012) | 0.0349 (0.00011) | 0.823 (0.0008) |
|  |  |  |  | 80 | -0.400 (0.0012) | 0.0350 (0.00011) | 0.823 (0.0009) |
|  |  |  |  | 90 | -0.400 (0.0012) | 0.0349 (0.00011) | 0.823 (0.0008) |
|  |  |  | 0.3 | 0 | -0.378 (0.0012) | 0.0348 (0.00011) | 0.819 (0.0009) |
|  |  |  |  | 10 | -0.379 (0.0012) | 0.0346 (0.00011) | 0.818 (0.0009) |
|  |  |  |  | 20 | -0.381 (0.0012) | 0.0346 (0.00011) | 0.819 (0.0009) |
|  |  |  |  | 30 | -0.382 (0.0012) | 0.0349 (0.00011) | 0.822 (0.0009) |
|  |  |  |  | 40 | -0.383 (0.0012) | 0.0346 (0.00011) | 0.819 (0.0009) |
|  |  |  |  | 50 | -0.385 (0.0012) | 0.0347 (0.00011) | 0.821 (0.0009) |
|  |  |  |  | 60 | -0.386 (0.0012) | 0.0348 (0.00011) | 0.821 (0.0009) |
|  |  |  |  | 70 | -0.388 (0.0012) | 0.0349 (0.00011) | 0.822 (0.0009) |
|  |  |  |  | 80 | -0.390 (0.0012) | 0.0349 (0.00011) | 0.822 (0.0009) |
|  |  |  |  | 90 | -0.392 (0.0012) | 0.0350 (0.00011) | 0.823 (0.0009) |
|  |  |  | 0.5 | 0 | -0.332 (0.0011) | 0.0338 (0.00010) | 0.800 (0.0010) |
|  |  |  |  | 10 | -0.335 (0.0011) | 0.0338 (0.00010) | 0.804 (0.0010) |
|  |  |  |  | 20 | -0.339 (0.0011) | 0.0341 (0.00010) | 0.810 (0.0009) |
|  |  |  |  | 30 | -0.344 (0.0011) | 0.0342 (0.00010) | 0.814 (0.0009) |
|  |  |  |  | 40 | -0.349 (0.0011) | 0.0342 (0.00010) | 0.816 (0.0009) |
|  |  |  |  | 50 | -0.354 (0.0011) | 0.0343 (0.00010) | 0.818 (0.0009) |
|  |  |  |  | 60 | -0.359 (0.0011) | 0.0344 (0.00010) | 0.820 (0.0009) |
|  |  |  |  | 70 | -0.365 (0.0011) | 0.0345 (0.00010) | 0.821 (0.0009) |
|  |  |  |  | 80 | -0.372 (0.0011) | 0.0345 (0.00010) | 0.822 (0.0008) |
|  |  |  |  | 90 | -0.381 (0.0011) | 0.0348 (0.00011) | 0.824 (0.0009) |
|  |  |  | 0.7 | 0 | -0.193 (0.0009) | 0.0311 (0.00007) | 0.660 (0.0016) |
|  |  |  |  | 10 | -0.196 (0.0009) | 0.0316 (0.00008) | 0.684 (0.0015) |
|  |  |  |  | 20 | -0.203 (0.0010) | 0.0323 (0.00008) | 0.714 (0.0015) |
|  |  |  |  | 30 | -0.214 (0.0011) | 0.0331 (0.00009) | 0.746 (0.0014) |
|  |  |  |  | 40 | -0.228 (0.0011) | 0.0338 (0.00009) | 0.773 (0.0013) |
|  |  |  |  | 50 | -0.246 (0.0012) | 0.0343 (0.00010) | 0.795 (0.0011) |
|  |  |  |  | 60 | -0.266 (0.0012) | 0.0347 (0.00010) | 0.809 (0.0010) |
|  |  |  |  | 70 | -0.288 (0.0012) | 0.0347 (0.00011) | 0.817 (0.0010) |
|  |  |  |  | 80 | -0.312 (0.0012) | 0.0348 (0.00011) | 0.823 (0.0009) |
|  |  |  |  | 90 | -0.342 (0.0011) | 0.0356 (0.00012) | 0.830 (0.0009) |
|  |  | 2 – smaller X-Y correlation (0.2) | 0.1 | 0 | -0.125 (0.0010) | 0.0273 (0.00006) | 0.516 (0.0014) |
|  |  |  |  | 10 | -0.125 (0.0010) | 0.0273 (0.00006) | 0.516 (0.0014) |
|  |  |  |  | 20 | -0.125 (0.0010) | 0.0273 (0.00006) | 0.515 (0.0014) |
|  |  |  |  | 30 | -0.125 (0.0010) | 0.0273 (0.00006) | 0.515 (0.0014) |
|  |  |  |  | 40 | -0.125 (0.0010) | 0.0273 (0.00006) | 0.516 (0.0015) |
|  |  |  |  | 50 | -0.125 (0.0010) | 0.0273 (0.00006) | 0.516 (0.0014) |
|  |  |  |  | 60 | -0.125 (0.0010) | 0.0273 (0.00006) | 0.515 (0.0014) |
|  |  |  |  | 70 | -0.125 (0.0010) | 0.0273 (0.00006) | 0.515 (0.0015) |
|  |  |  |  | 80 | -0.125 (0.0010) | 0.0273 (0.00006) | 0.515 (0.0014) |
|  |  |  |  | 90 | -0.126 (0.0010) | 0.0273 (0.00005) | 0.516 (0.0014) |
|  |  |  | 0.3 | 0 | -0.121 (0.0009) | 0.0271 (0.00006) | 0.508 (0.0014) |
|  |  |  |  | 10 | -0.121 (0.0010) | 0.0271 (0.00006) | 0.510 (0.0014) |
|  |  |  |  | 20 | -0.122 (0.0010) | 0.0271 (0.00005) | 0.510 (0.0014) |
|  |  |  |  | 30 | -0.122 (0.0010) | 0.0271 (0.00006) | 0.510 (0.0015) |
|  |  |  |  | 40 | -0.122 (0.0010) | 0.0271 (0.00005) | 0.511 (0.0014) |
|  |  |  |  | 50 | -0.123 (0.0010) | 0.0272 (0.00006) | 0.513 (0.0014) |
|  |  |  |  | 60 | -0.123 (0.0010) | 0.0272 (0.00005) | 0.515 (0.0014) |
|  |  |  |  | 70 | -0.123 (0.0010) | 0.0272 (0.00006) | 0.514 (0.0014) |
|  |  |  |  | 80 | -0.124 (0.0010) | 0.0273 (0.00006) | 0.515 (0.0015) |
|  |  |  |  | 90 | -0.124 (0.0010) | 0.0272 (0.00005) | 0.514 (0.0014) |
|  |  |  | 0.5 | 0 | -0.112 (0.0009) | 0.0268 (0.00005) | 0.484 (0.0014) |
|  |  |  |  | 10 | -0.113 (0.0009) | 0.0268 (0.00005) | 0.490 (0.0014) |
|  |  |  |  | 20 | -0.114 (0.0009) | 0.0269 (0.00005) | 0.495 (0.0014) |
|  |  |  |  | 30 | -0.115 (0.0009) | 0.0269 (0.00005) | 0.499 (0.0014) |
|  |  |  |  | 40 | -0.116 (0.0009) | 0.0269 (0.00005) | 0.500 (0.0015) |
|  |  |  |  | 50 | -0.117 (0.0009) | 0.0270 (0.00005) | 0.504 (0.0014) |
|  |  |  |  | 60 | -0.118 (0.0009) | 0.0270 (0.00005) | 0.506 (0.0014) |
|  |  |  |  | 70 | -0.119 (0.0009) | 0.0271 (0.00005) | 0.509 (0.0014) |
|  |  |  |  | 80 | -0.120 (0.0009) | 0.0272 (0.00005) | 0.512 (0.0014) |
|  |  |  |  | 90 | -0.122 (0.0009) | 0.0272 (0.00006) | 0.512 (0.0014) |
|  |  |  | 0.7 | 0 | -0.091 (0.0008) | 0.0264 (0.00005) | 0.412 (0.0014) |
|  |  |  |  | 10 | -0.093 (0.0008) | 0.0263 (0.00005) | 0.428 (0.0014) |
|  |  |  |  | 20 | -0.095 (0.0008) | 0.0263 (0.00005) | 0.444 (0.0015) |
|  |  |  |  | 30 | -0.097 (0.0008) | 0.0264 (0.00005) | 0.458 (0.0014) |
|  |  |  |  | 40 | -0.099 (0.0009) | 0.0264 (0.00005) | 0.470 (0.0014) |
|  |  |  |  | 50 | -0.102 (0.0009) | 0.0264 (0.00005) | 0.477 (0.0014) |
|  |  |  |  | 60 | -0.105 (0.0009) | 0.0265 (0.00005) | 0.482 (0.0014) |
|  |  |  |  | 70 | -0.108 (0.0009) | 0.0266 (0.00005) | 0.490 (0.0015) |
|  |  |  |  | 80 | -0.112 (0.0009) | 0.0267 (0.00005) | 0.496 (0.0014) |
|  |  |  |  | 90 | -0.116 (0.0009) | 0.0270 (0.00005) | 0.503 (0.0015) |
| Mechanism 2 – missingness in auxiliary, Z, caused by the auxiliary itself | Mechanism 3 – missingness in outcome, Y, is caused by the exposure variable, X, only | 1 – more missing outcome (75%) | 0.1 | 0 | 0.004 (0.0032) | 0.1045 (0.00035) | 0.941 (0.0004) |
|  |  |  |  | 10 | 0.005 (0.0032) | 0.1052 (0.00036) | 0.942 (0.0003) |
|  |  |  |  | 20 | 0.005 (0.0033) | 0.1051 (0.00035) | 0.942 (0.0003) |
|  |  |  |  | 30 | 0.004 (0.0033) | 0.1056 (0.00035) | 0.942 (0.0003) |
|  |  |  |  | 40 | 0.005 (0.0033) | 0.1054 (0.00034) | 0.942 (0.0003) |
|  |  |  |  | 50 | 0.005 (0.0033) | 0.1056 (0.00035) | 0.942 (0.0003) |
|  |  |  |  | 60 | 0.004 (0.0033) | 0.1054 (0.00036) | 0.942 (0.0003) |
|  |  |  |  | 70 | 0.005 (0.0033) | 0.1055 (0.00035) | 0.942 (0.0003) |
|  |  |  |  | 80 | 0.005 (0.0033) | 0.1061 (0.00035) | 0.943 (0.0003) |
|  |  |  |  | 90 | 0.003 (0.0032) | 0.1060 (0.00036) | 0.943 (0.0003) |
|  |  |  | 0.3 | 0 | 0.002 (0.0031) | 0.0983 (0.00032) | 0.933 (0.0004) |
|  |  |  |  | 10 | 0.001 (0.0031) | 0.1002 (0.00033) | 0.936 (0.0004) |
|  |  |  |  | 20 | -0.000 (0.0032) | 0.1015 (0.00034) | 0.937 (0.0004) |
|  |  |  |  | 30 | 0.001 (0.0032) | 0.1025 (0.00033) | 0.939 (0.0003) |
|  |  |  |  | 40 | 0.000 (0.0032) | 0.1030 (0.00034) | 0.939 (0.0003) |
|  |  |  |  | 50 | 0.001 (0.0032) | 0.1039 (0.00034) | 0.940 (0.0003) |
|  |  |  |  | 60 | 0.001 (0.0033) | 0.1041 (0.00035) | 0.941 (0.0003) |
|  |  |  |  | 70 | 0.002 (0.0033) | 0.1048 (0.00035) | 0.941 (0.0003) |
|  |  |  |  | 80 | 0.002 (0.0033) | 0.1047 (0.00034) | 0.941 (0.0003) |
|  |  |  |  | 90 | 0.003 (0.0032) | 0.1051 (0.00035) | 0.942 (0.0003) |
|  |  |  | 0.5 | 0 | 0.002 (0.0025) | 0.0835 (0.00028) | 0.907 (0.0006) |
|  |  |  |  | 10 | -0.007 (0.0027) | 0.0886 (0.00030) | 0.918 (0.0005) |
|  |  |  |  | 20 | -0.012 (0.0028) | 0.0923 (0.00032) | 0.924 (0.0005) |
|  |  |  |  | 30 | -0.015 (0.0029) | 0.0949 (0.00033) | 0.929 (0.0004) |
|  |  |  |  | 40 | -0.015 (0.0030) | 0.0978 (0.00034) | 0.933 (0.0004) |
|  |  |  |  | 50 | -0.012 (0.0030) | 0.0997 (0.00034) | 0.935 (0.0004) |
|  |  |  |  | 60 | -0.009 (0.0031) | 0.1015 (0.00035) | 0.938 (0.0004) |
|  |  |  |  | 70 | -0.005 (0.0032) | 0.1030 (0.00034) | 0.940 (0.0003) |
|  |  |  |  | 80 | -0.002 (0.0032) | 0.1044 (0.00036) | 0.941 (0.0003) |
|  |  |  |  | 90 | 0.000 (0.0032) | 0.1050 (0.00035) | 0.942 (0.0003) |
|  |  |  | 0.7 | 0 | 0.000 (0.0016) | 0.0556 (0.00016) | 0.792 (0.0011) |
|  |  |  |  | 10 | -0.029 (0.0018) | 0.0592 (0.00019) | 0.829 (0.0010) |
|  |  |  |  | 20 | -0.051 (0.0020) | 0.0637 (0.00021) | 0.854 (0.0009) |
|  |  |  |  | 30 | -0.065 (0.0021) | 0.0689 (0.00023) | 0.874 (0.0008) |
|  |  |  |  | 40 | -0.073 (0.0023) | 0.0753 (0.00027) | 0.892 (0.0007) |
|  |  |  |  | 50 | -0.074 (0.0026) | 0.0827 (0.00029) | 0.909 (0.0006) |
|  |  |  |  | 60 | -0.064 (0.0029) | 0.0913 (0.00032) | 0.924 (0.0005) |
|  |  |  |  | 70 | -0.043 (0.0030) | 0.0989 (0.00032) | 0.935 (0.0004) |
|  |  |  |  | 80 | -0.021 (0.0032) | 0.1027 (0.00033) | 0.940 (0.0003) |
|  |  |  |  | 90 | -0.006 (0.0032) | 0.1043 (0.00034) | 0.941 (0.0003) |
|  |  | 2 – smaller X-Y correlation (0.2) | 0.1 | 0 | -0.001 (0.0022) | 0.0732 (0.00018) | 0.820 (0.0008) |
|  |  |  |  | 10 | -0.001 (0.0022) | 0.0735 (0.00018) | 0.822 (0.0008) |
|  |  |  |  | 20 | -0.000 (0.0022) | 0.0732 (0.00017) | 0.821 (0.0007) |
|  |  |  |  | 30 | -0.001 (0.0022) | 0.0733 (0.00018) | 0.821 (0.0008) |
|  |  |  |  | 40 | -0.000 (0.0022) | 0.0735 (0.00019) | 0.822 (0.0008) |
|  |  |  |  | 50 | -0.000 (0.0022) | 0.0735 (0.00018) | 0.822 (0.0008) |
|  |  |  |  | 60 | -0.000 (0.0022) | 0.0737 (0.00018) | 0.823 (0.0008) |
|  |  |  |  | 70 | -0.000 (0.0022) | 0.0735 (0.00018) | 0.822 (0.0007) |
|  |  |  |  | 80 | -0.000 (0.0022) | 0.0736 (0.00018) | 0.823 (0.0008) |
|  |  |  |  | 90 | -0.001 (0.0022) | 0.0734 (0.00019) | 0.821 (0.0008) |
|  |  |  | 0.3 | 0 | 0.003 (0.0020) | 0.0705 (0.00018) | 0.806 (0.0008) |
|  |  |  |  | 10 | 0.002 (0.0020) | 0.0713 (0.00017) | 0.810 (0.0008) |
|  |  |  |  | 20 | 0.002 (0.0020) | 0.0717 (0.00018) | 0.813 (0.0008) |
|  |  |  |  | 30 | 0.001 (0.0020) | 0.0723 (0.00018) | 0.816 (0.0008) |
|  |  |  |  | 40 | 0.001 (0.0021) | 0.0727 (0.00018) | 0.818 (0.0008) |
|  |  |  |  | 50 | 0.001 (0.0021) | 0.0731 (0.00019) | 0.820 (0.0008) |
|  |  |  |  | 60 | 0.001 (0.0021) | 0.0733 (0.00019) | 0.821 (0.0008) |
|  |  |  |  | 70 | 0.002 (0.0021) | 0.0733 (0.00018) | 0.821 (0.0008) |
|  |  |  |  | 80 | 0.002 (0.0021) | 0.0734 (0.00019) | 0.821 (0.0008) |
|  |  |  |  | 90 | 0.002 (0.0021) | 0.0734 (0.00019) | 0.821 (0.0008) |
|  |  |  | 0.5 | 0 | -0.001 (0.0018) | 0.0655 (0.00016) | 0.775 (0.0009) |
|  |  |  |  | 10 | -0.009 (0.0019) | 0.0677 (0.00017) | 0.789 (0.0009) |
|  |  |  |  | 20 | -0.012 (0.0019) | 0.0691 (0.00017) | 0.798 (0.0009) |
|  |  |  |  | 30 | -0.014 (0.0020) | 0.0700 (0.00017) | 0.803 (0.0008) |
|  |  |  |  | 40 | -0.014 (0.0020) | 0.0710 (0.00018) | 0.808 (0.0008) |
|  |  |  |  | 50 | -0.012 (0.0020) | 0.0717 (0.00017) | 0.812 (0.0008) |
|  |  |  |  | 60 | -0.010 (0.0021) | 0.0725 (0.00018) | 0.816 (0.0008) |
|  |  |  |  | 70 | -0.008 (0.0021) | 0.0730 (0.00018) | 0.819 (0.0008) |
|  |  |  |  | 80 | -0.005 (0.0021) | 0.0735 (0.00019) | 0.821 (0.0008) |
|  |  |  |  | 90 | -0.003 (0.0021) | 0.0734 (0.00018) | 0.821 (0.0008) |
|  |  |  | 0.7 | 0 | -0.000 (0.0015) | 0.0560 (0.00012) | 0.692 (0.0012) |
|  |  |  |  | 10 | -0.024 (0.0016) | 0.0591 (0.00013) | 0.726 (0.0011) |
|  |  |  |  | 20 | -0.036 (0.0017) | 0.0616 (0.00014) | 0.748 (0.0010) |
|  |  |  |  | 30 | -0.043 (0.0018) | 0.0637 (0.00016) | 0.764 (0.0010) |
|  |  |  |  | 40 | -0.045 (0.0019) | 0.0660 (0.00016) | 0.780 (0.0009) |
|  |  |  |  | 50 | -0.042 (0.0019) | 0.0679 (0.00017) | 0.791 (0.0009) |
|  |  |  |  | 60 | -0.037 (0.0020) | 0.0699 (0.00017) | 0.802 (0.0008) |
|  |  |  |  | 70 | -0.029 (0.0020) | 0.0716 (0.00017) | 0.812 (0.0008) |
|  |  |  |  | 80 | -0.019 (0.0021) | 0.0729 (0.00018) | 0.818 (0.0008) |
|  |  |  |  | 90 | -0.009 (0.0021) | 0.0735 (0.00018) | 0.821 (0.0008) |

CRA = Complete Records Analysis; FMI = Fraction of Missing Information; MCSE = Monte Carlo Standard Error; MI = Multiple imputation; SE = Standard Error


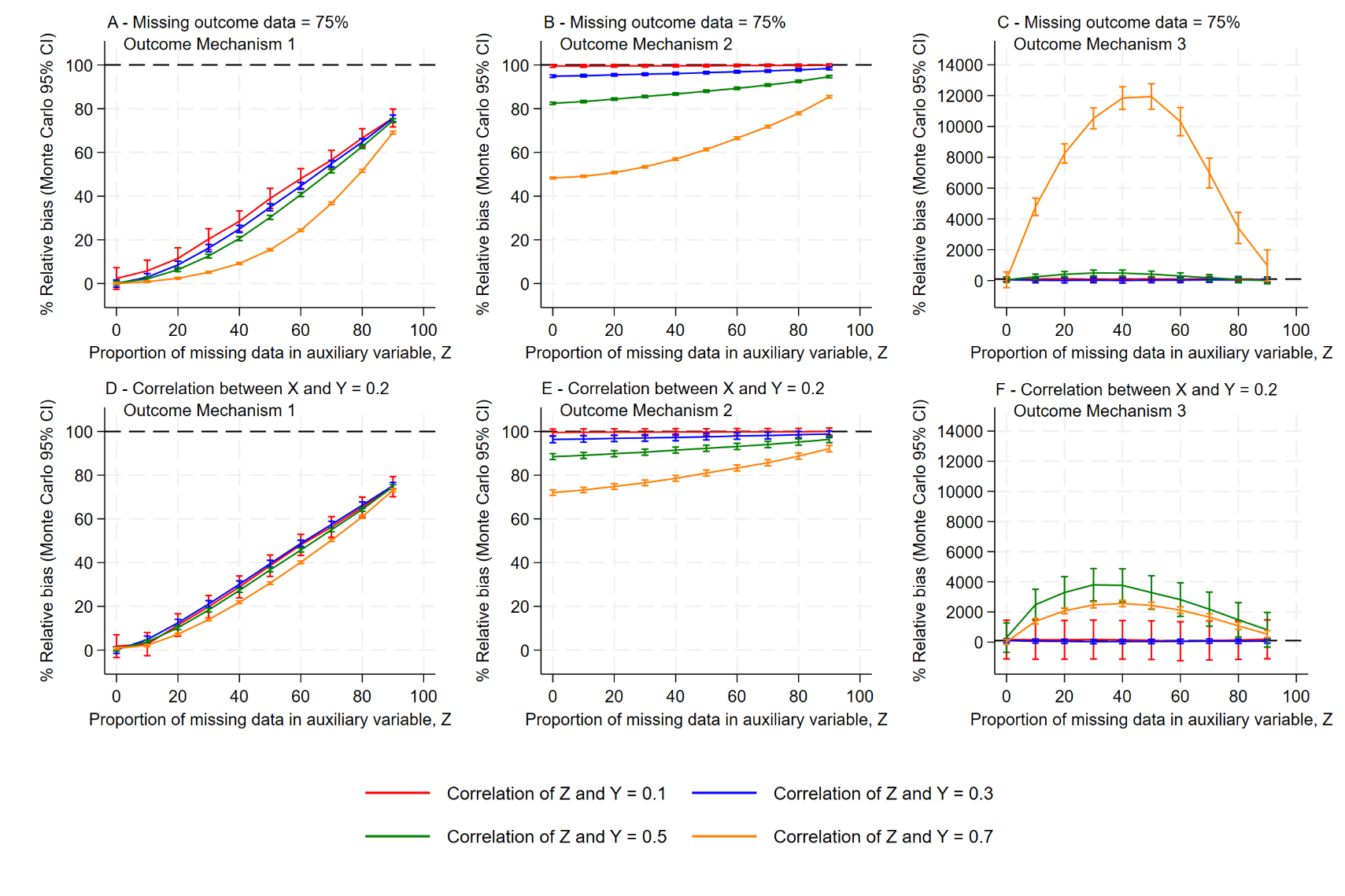


#### **Figure S5**: Plots of bias relative to CCA for the exposure (X) coefficient against the proportion of missing data in Z for sensitivity analysis that i) increased the missing data in the outcome to 75% (plots A-C) and ii) decreased the correlation between X and Y to 0.2 (plots D-E).

The missing auxiliary missing data mechanism is auxiliary mechanism 3 for plots A, B, D and E and auxiliary mechanism 2 for plots C and F.


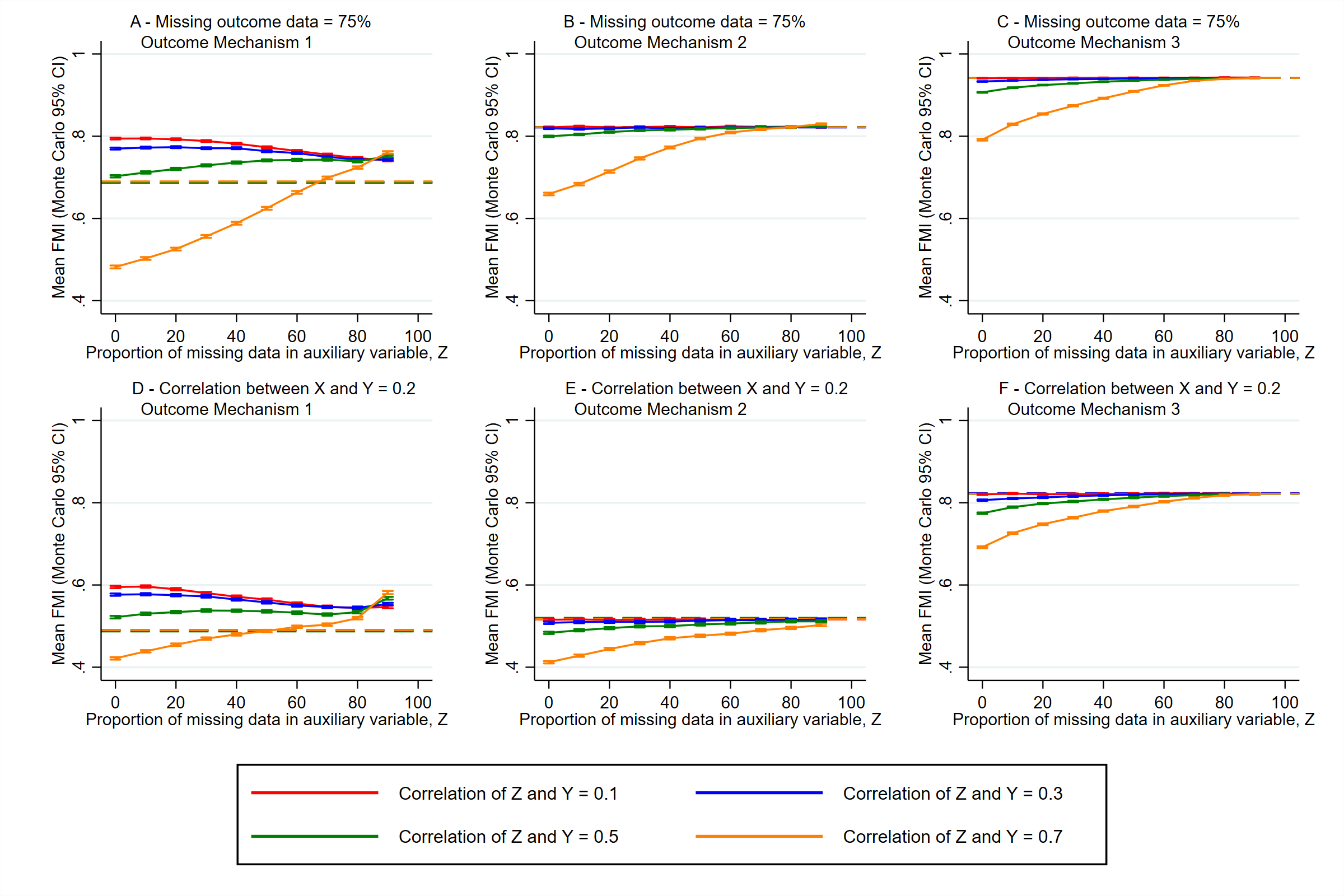


#### **Figure S6**: Plots of the FMI for the exposure (X) coefficient against the proportion of missing data in Z for sensitivity analysis that i) increased the missing data in the outcome to 75% (plots A-C) and ii) decreased the correlation between X and Y to 0.2 (plots D-E).

The missing auxiliary missing data mechanism is auxiliary mechanism 3 for plots A, B, D and E and auxiliary mechanism 2 for plots C and F.

#### Bias and FMI when excluding W from the imputation model under missing auxiliary mechanism 3

We additionally assessed the impact of excluding W from the imputation model under missing auxiliary mechanism 3 in which Z was independent of its own missingness conditional on W. Values of bias, SE and the FMI under MI models that excluded or included W are presented in Table S10 while plots of bias for this sensitivity analysis are presented in Figure S7 and plots of the FMI are presented in Figure S8. For Figure S7 we present bias as relative to CRA for outcome mechanisms 1 and 2 (where CRA is biased) and as absolute bias for outcome mechanism 3 (where CRA is unbiased) to highlight the magnitude of bias being introduced and to avoid showing relative values many thousand times a very small amount of bias in CRA. When W was excluded from the imputation model bias was increased at all proportions of missing auxiliary data for all missing outcome mechanisms, compared to when W was included in the imputation model (which makes Z independent of its own missingness). For auxiliary mechanism 3, as in primary analysis under auxiliary mechanism 2, the bias decreased after the amount of missing data in the auxiliary reached a certain point (here that value was 60% as opposed to 50% in primary analyses).

Inclusion of W in the imputation model led to FMI values that were larger than in MI models excluding all auxiliaries for large proportions of missing data in Z (>80%) under missing outcome mechanism 1 and 2; this was more pronounced when there were stronger correlations between Z and Y. Exclusion of W from the imputation model lead to the FMI converging to the FMI value of models excluding all auxiliaries as the proportion of missing data in Z increased.

#### **Table S10**: Sensitivity analysis - average bias, standard error and fraction of missing information across simulations for the exposure (X) coefficient for multiple imputation models that excluded or included W in the imputation model under missing auxiliary mechanism 3

| Missing outcome mechanism | Correlation between Z and Y | Proportion of missing auxiliary data | Imputation model **excluding** W | | | MI model **including** W | | |
| --- | --- | --- | --- | --- | --- | --- | --- | --- |
|  |  |  | Bias (MCSE) | Standard error (MCSE) | FMI (MCSE) | Bias (MCSE) | Standard error (MCSE) | FMI (MCSE) |
| Mechanism 1 | 0.1 | 0 | -0.001 (0.0010) | 0.0399 (0.00009) | 0.594 (0.0014) | -0.002 (0.0010) | 0.0400 (0.00009) | 0.597 (0.0014) |
|  |  | 10 | -0.005 (0.0010) | 0.0398 (0.00009) | 0.590 (0.0015) | -0.003 (0.0010) | 0.0398 (0.00009) | 0.594 (0.0014) |
|  |  | 20 | -0.009 (0.0009) | 0.0393 (0.00009) | 0.582 (0.0015) | -0.007 (0.0010) | 0.0396 (0.00009) | 0.588 (0.0015) |
|  |  | 30 | -0.013 (0.0009) | 0.0389 (0.00008) | 0.572 (0.0015) | -0.011 (0.0010) | 0.0391 (0.00009) | 0.580 (0.0015) |
|  |  | 40 | -0.018 (0.0009) | 0.0384 (0.00008) | 0.561 (0.0015) | -0.015 (0.0010) | 0.0388 (0.00008) | 0.572 (0.0015) |
|  |  | 50 | -0.023 (0.0009) | 0.0379 (0.00008) | 0.551 (0.0015) | -0.020 (0.0009) | 0.0384 (0.00008) | 0.565 (0.0014) |
|  |  | 60 | -0.028 (0.0008) | 0.0374 (0.00008) | 0.538 (0.0015) | -0.024 (0.0009) | 0.0381 (0.00008) | 0.557 (0.0015) |
|  |  | 70 | -0.032 (0.0008) | 0.0368 (0.00007) | 0.523 (0.0015) | -0.027 (0.0009) | 0.0378 (0.00008) | 0.550 (0.0015) |
|  |  | 80 | -0.037 (0.0008) | 0.0364 (0.00007) | 0.512 (0.0015) | -0.031 (0.0009) | 0.0376 (0.00008) | 0.546 (0.0015) |
|  |  | 90 | -0.041 (0.0008) | 0.0359 (0.00007) | 0.499 (0.0015) | -0.036 (0.0009) | 0.0377 (0.00008) | 0.548 (0.0015) |
|  | 0.3 | 0 | -0.000 (0.0009) | 0.0385 (0.00008) | 0.565 (0.0015) | -0.001 (0.0009) | 0.0385 (0.00008) | 0.563 (0.0015) |
|  |  | 10 | -0.012 (0.0009) | 0.0384 (0.00008) | 0.562 (0.0015) | -0.007 (0.0009) | 0.0385 (0.00008) | 0.565 (0.0014) |
|  |  | 20 | -0.024 (0.0009) | 0.0381 (0.00008) | 0.557 (0.0015) | -0.017 (0.0009) | 0.0384 (0.00008) | 0.565 (0.0015) |
|  |  | 30 | -0.037 (0.0009) | 0.0377 (0.00008) | 0.550 (0.0015) | -0.028 (0.0009) | 0.0382 (0.00008) | 0.562 (0.0015) |
|  |  | 40 | -0.051 (0.0009) | 0.0374 (0.00008) | 0.544 (0.0015) | -0.041 (0.0009) | 0.0378 (0.00008) | 0.557 (0.0015) |
|  |  | 50 | -0.066 (0.0009) | 0.0370 (0.00008) | 0.535 (0.0016) | -0.054 (0.0009) | 0.0376 (0.00008) | 0.553 (0.0015) |
|  |  | 60 | -0.080 (0.0008) | 0.0366 (0.00008) | 0.526 (0.0016) | -0.067 (0.0009) | 0.0373 (0.00007) | 0.547 (0.0015) |
|  |  | 70 | -0.095 (0.0008) | 0.0361 (0.00007) | 0.515 (0.0015) | -0.079 (0.0009) | 0.0370 (0.00008) | 0.541 (0.0016) |
|  |  | 80 | -0.110 (0.0008) | 0.0357 (0.00007) | 0.505 (0.0015) | -0.092 (0.0009) | 0.0370 (0.00007) | 0.542 (0.0014) |
|  |  | 90 | -0.125 (0.0008) | 0.0353 (0.00007) | 0.496 (0.0015) | -0.105 (0.0009) | 0.0378 (0.00008) | 0.560 (0.0015) |
|  | 0.5 | 0 | 0.001 (0.0008) | 0.0352 (0.00007) | 0.476 (0.0016) | 0.001 (0.0008) | 0.0351 (0.00006) | 0.479 (0.0015) |
|  |  | 10 | -0.017 (0.0008) | 0.0353 (0.00007) | 0.483 (0.0016) | -0.007 (0.0008) | 0.0353 (0.00007) | 0.489 (0.0016) |
|  |  | 20 | -0.036 (0.0008) | 0.0351 (0.00007) | 0.483 (0.0016) | -0.021 (0.0008) | 0.0355 (0.00007) | 0.500 (0.0016) |
|  |  | 30 | -0.056 (0.0008) | 0.0350 (0.00007) | 0.483 (0.0016) | -0.038 (0.0008) | 0.0355 (0.00007) | 0.508 (0.0016) |
|  |  | 40 | -0.079 (0.0008) | 0.0350 (0.00007) | 0.487 (0.0015) | -0.057 (0.0008) | 0.0354 (0.00007) | 0.512 (0.0015) |
|  |  | 50 | -0.103 (0.0008) | 0.0350 (0.00007) | 0.491 (0.0016) | -0.078 (0.0008) | 0.0352 (0.00007) | 0.514 (0.0015) |
|  |  | 60 | -0.128 (0.0008) | 0.0348 (0.00007) | 0.489 (0.0016) | -0.100 (0.0008) | 0.0352 (0.00007) | 0.518 (0.0016) |
|  |  | 70 | -0.154 (0.0008) | 0.0346 (0.00007) | 0.488 (0.0016) | -0.122 (0.0008) | 0.0352 (0.00007) | 0.520 (0.0015) |
|  |  | 80 | -0.181 (0.0008) | 0.0345 (0.00007) | 0.488 (0.0015) | -0.144 (0.0008) | 0.0355 (0.00007) | 0.529 (0.0015) |
|  |  | 90 | -0.208 (0.0008) | 0.0343 (0.00007) | 0.487 (0.0015) | -0.169 (0.0009) | 0.0376 (0.00010) | 0.576 (0.0018) |
|  | 0.7 | 0 | 0.000 (0.0005) | 0.0295 (0.00004) | 0.261 (0.0012) | 0.001 (0.0005) | 0.0295 (0.00004) | 0.260 (0.0012) |
|  |  | 10 | -0.024 (0.0005) | 0.0295 (0.00004) | 0.284 (0.0013) | -0.005 (0.0005) | 0.0300 (0.00004) | 0.288 (0.0013) |
|  |  | 20 | -0.047 (0.0005) | 0.0299 (0.00004) | 0.310 (0.0013) | -0.016 (0.0005) | 0.0304 (0.00005) | 0.315 (0.0014) |
|  |  | 30 | -0.073 (0.0006) | 0.0302 (0.00004) | 0.331 (0.0013) | -0.031 (0.0006) | 0.0308 (0.00005) | 0.344 (0.0014) |
|  |  | 40 | -0.101 (0.0006) | 0.0306 (0.00005) | 0.355 (0.0013) | -0.050 (0.0006) | 0.0312 (0.00005) | 0.374 (0.0014) |
|  |  | 50 | -0.132 (0.0006) | 0.0310 (0.00005) | 0.380 (0.0013) | -0.076 (0.0007) | 0.0316 (0.00005) | 0.401 (0.0015) |
|  |  | 60 | -0.167 (0.0007) | 0.0314 (0.00005) | 0.402 (0.0014) | -0.106 (0.0007) | 0.0320 (0.00006) | 0.431 (0.0015) |
|  |  | 70 | -0.205 (0.0007) | 0.0317 (0.00006) | 0.424 (0.0014) | -0.141 (0.0008) | 0.0326 (0.00006) | 0.460 (0.0015) |
|  |  | 80 | -0.246 (0.0007) | 0.0322 (0.00006) | 0.450 (0.0015) | -0.184 (0.0009) | 0.0337 (0.00008) | 0.502 (0.0019) |
|  |  | 90 | -0.288 (0.0008) | 0.0325 (0.00006) | 0.470 (0.0014) | -0.234 (0.0014) | 0.0385 (0.00019) | 0.600 (0.0030) |
| Mechanism 2 | 0.1 | 0 | -0.314 (0.0008) | 0.0282 (0.00006) | 0.618 (0.0013) | -0.315 (0.0009) | 0.0281 (0.00006) | 0.616 (0.0013) |
|  |  | 10 | -0.314 (0.0008) | 0.0282 (0.00006) | 0.618 (0.0013) | -0.315 (0.0009) | 0.0281 (0.00006) | 0.616 (0.0013) |
|  |  | 20 | -0.315 (0.0008) | 0.0281 (0.00006) | 0.616 (0.0013) | -0.316 (0.0009) | 0.0280 (0.00006) | 0.615 (0.0013) |
|  |  | 30 | -0.315 (0.0008) | 0.0282 (0.00006) | 0.617 (0.0013) | -0.316 (0.0009) | 0.0281 (0.00006) | 0.615 (0.0013) |
|  |  | 40 | -0.315 (0.0008) | 0.0281 (0.00006) | 0.615 (0.0013) | -0.316 (0.0009) | 0.0281 (0.00006) | 0.615 (0.0013) |
|  |  | 50 | -0.315 (0.0008) | 0.0282 (0.00006) | 0.618 (0.0013) | -0.316 (0.0009) | 0.0281 (0.00006) | 0.615 (0.0014) |
|  |  | 60 | -0.316 (0.0008) | 0.0282 (0.00006) | 0.619 (0.0013) | -0.316 (0.0009) | 0.0281 (0.00006) | 0.617 (0.0013) |
|  |  | 70 | -0.316 (0.0008) | 0.0282 (0.00006) | 0.619 (0.0013) | -0.316 (0.0009) | 0.0281 (0.00006) | 0.618 (0.0014) |
|  |  | 80 | -0.316 (0.0008) | 0.0281 (0.00006) | 0.617 (0.0013) | -0.317 (0.0009) | 0.0281 (0.00006) | 0.617 (0.0014) |
|  |  | 90 | -0.316 (0.0008) | 0.0281 (0.00006) | 0.615 (0.0013) | -0.317 (0.0009) | 0.0281 (0.00006) | 0.616 (0.0013) |
|  | 0.3 | 0 | -0.294 (0.0008) | 0.0278 (0.00006) | 0.606 (0.0013) | -0.295 (0.0008) | 0.0280 (0.00006) | 0.608 (0.0013) |
|  |  | 10 | -0.297 (0.0008) | 0.0278 (0.00005) | 0.605 (0.0013) | -0.296 (0.0008) | 0.0280 (0.00006) | 0.610 (0.0013) |
|  |  | 20 | -0.299 (0.0008) | 0.0279 (0.00006) | 0.609 (0.0014) | -0.298 (0.0008) | 0.0280 (0.00006) | 0.611 (0.0013) |
|  |  | 30 | -0.301 (0.0008) | 0.0279 (0.00006) | 0.610 (0.0013) | -0.299 (0.0008) | 0.0281 (0.00006) | 0.614 (0.0013) |
|  |  | 40 | -0.303 (0.0008) | 0.0279 (0.00006) | 0.610 (0.0013) | -0.301 (0.0008) | 0.0280 (0.00006) | 0.613 (0.0013) |
|  |  | 50 | -0.305 (0.0008) | 0.0280 (0.00006) | 0.612 (0.0013) | -0.303 (0.0008) | 0.0281 (0.00006) | 0.616 (0.0013) |
|  |  | 60 | -0.308 (0.0008) | 0.0280 (0.00006) | 0.614 (0.0013) | -0.305 (0.0008) | 0.0281 (0.00006) | 0.616 (0.0013) |
|  |  | 70 | -0.310 (0.0008) | 0.0280 (0.00006) | 0.613 (0.0013) | -0.307 (0.0008) | 0.0281 (0.00006) | 0.614 (0.0013) |
|  |  | 80 | -0.312 (0.0008) | 0.0281 (0.00006) | 0.616 (0.0013) | -0.309 (0.0008) | 0.0282 (0.00006) | 0.618 (0.0013) |
|  |  | 90 | -0.314 (0.0008) | 0.0282 (0.00006) | 0.618 (0.0013) | -0.311 (0.0008) | 0.0283 (0.00006) | 0.619 (0.0013) |
|  | 0.5 | 0 | -0.243 (0.0007) | 0.0271 (0.00005) | 0.563 (0.0015) | -0.242 (0.0008) | 0.0272 (0.00005) | 0.562 (0.0014) |
|  |  | 10 | -0.249 (0.0007) | 0.0272 (0.00006) | 0.572 (0.0014) | -0.246 (0.0008) | 0.0272 (0.00005) | 0.572 (0.0014) |
|  |  | 20 | -0.255 (0.0008) | 0.0273 (0.00005) | 0.580 (0.0014) | -0.250 (0.0008) | 0.0273 (0.00006) | 0.580 (0.0014) |
|  |  | 30 | -0.261 (0.0008) | 0.0274 (0.00006) | 0.585 (0.0014) | -0.255 (0.0008) | 0.0274 (0.00006) | 0.587 (0.0014) |
|  |  | 40 | -0.268 (0.0008) | 0.0274 (0.00006) | 0.588 (0.0014) | -0.261 (0.0008) | 0.0274 (0.00006) | 0.592 (0.0013) |
|  |  | 50 | -0.275 (0.0008) | 0.0275 (0.00006) | 0.594 (0.0014) | -0.266 (0.0008) | 0.0275 (0.00006) | 0.595 (0.0014) |
|  |  | 60 | -0.282 (0.0008) | 0.0277 (0.00006) | 0.600 (0.0014) | -0.272 (0.0008) | 0.0277 (0.00006) | 0.602 (0.0014) |
|  |  | 70 | -0.290 (0.0008) | 0.0278 (0.00006) | 0.606 (0.0013) | -0.278 (0.0008) | 0.0277 (0.00006) | 0.604 (0.0014) |
|  |  | 80 | -0.298 (0.0008) | 0.0279 (0.00006) | 0.608 (0.0014) | -0.285 (0.0008) | 0.0279 (0.00006) | 0.609 (0.0014) |
|  |  | 90 | -0.308 (0.0008) | 0.0281 (0.00006) | 0.614 (0.0013) | -0.294 (0.0009) | 0.0284 (0.00006) | 0.622 (0.0014) |
|  | 0.7 | 0 | -0.125 (0.0005) | 0.0259 (0.00004) | 0.361 (0.0013) | -0.125 (0.0005) | 0.0258 (0.00004) | 0.362 (0.0014) |
|  |  | 10 | -0.134 (0.0006) | 0.0258 (0.00004) | 0.399 (0.0014) | -0.127 (0.0006) | 0.0260 (0.00004) | 0.397 (0.0014) |
|  |  | 20 | -0.145 (0.0006) | 0.0260 (0.00004) | 0.430 (0.0015) | -0.132 (0.0006) | 0.0263 (0.00004) | 0.437 (0.0014) |
|  |  | 30 | -0.158 (0.0007) | 0.0261 (0.00004) | 0.457 (0.0014) | -0.141 (0.0007) | 0.0264 (0.00005) | 0.469 (0.0015) |
|  |  | 40 | -0.174 (0.0007) | 0.0263 (0.00005) | 0.483 (0.0014) | -0.153 (0.0007) | 0.0266 (0.00005) | 0.500 (0.0015) |
|  |  | 50 | -0.192 (0.0007) | 0.0266 (0.00005) | 0.510 (0.0014) | -0.169 (0.0007) | 0.0268 (0.00005) | 0.527 (0.0014) |
|  |  | 60 | -0.212 (0.0008) | 0.0268 (0.00005) | 0.533 (0.0014) | -0.187 (0.0008) | 0.0270 (0.00005) | 0.552 (0.0014) |
|  |  | 70 | -0.236 (0.0008) | 0.0270 (0.00005) | 0.554 (0.0014) | -0.208 (0.0008) | 0.0272 (0.00005) | 0.572 (0.0014) |
|  |  | 80 | -0.262 (0.0008) | 0.0273 (0.00006) | 0.577 (0.0014) | -0.232 (0.0008) | 0.0277 (0.00006) | 0.593 (0.0015) |
|  |  | 90 | -0.290 (0.0008) | 0.0277 (0.00006) | 0.598 (0.0013) | -0.264 (0.0009) | 0.0295 (0.00009) | 0.642 (0.0019) |
| Mechanism 3 | 0.1 | 0 | -0.000 (0.0017) | 0.0595 (0.00015) | 0.819 (0.0008) | -0.003 (0.0017) | 0.0596 (0.00015) | 0.819 (0.0008) |
|  |  | 10 | -0.000 (0.0017) | 0.0595 (0.00014) | 0.819 (0.0008) | -0.002 (0.0017) | 0.0596 (0.00014) | 0.819 (0.0008) |
|  |  | 20 | -0.001 (0.0018) | 0.0596 (0.00015) | 0.820 (0.0008) | -0.003 (0.0017) | 0.0598 (0.00015) | 0.820 (0.0008) |
|  |  | 30 | -0.001 (0.0017) | 0.0595 (0.00014) | 0.819 (0.0008) | -0.002 (0.0017) | 0.0597 (0.00015) | 0.820 (0.0008) |
|  |  | 40 | -0.000 (0.0018) | 0.0598 (0.00015) | 0.821 (0.0008) | -0.003 (0.0017) | 0.0599 (0.00015) | 0.821 (0.0008) |
|  |  | 50 | -0.001 (0.0018) | 0.0598 (0.00015) | 0.821 (0.0007) | -0.002 (0.0017) | 0.0596 (0.00014) | 0.819 (0.0008) |
|  |  | 60 | -0.000 (0.0018) | 0.0598 (0.00015) | 0.821 (0.0008) | -0.002 (0.0017) | 0.0597 (0.00015) | 0.820 (0.0008) |
|  |  | 70 | -0.001 (0.0018) | 0.0599 (0.00015) | 0.822 (0.0008) | -0.002 (0.0017) | 0.0598 (0.00014) | 0.820 (0.0008) |
|  |  | 80 | -0.000 (0.0018) | 0.0599 (0.00015) | 0.822 (0.0008) | -0.003 (0.0017) | 0.0597 (0.00015) | 0.820 (0.0008) |
|  |  | 90 | -0.001 (0.0018) | 0.0599 (0.00015) | 0.821 (0.0008) | -0.002 (0.0017) | 0.0598 (0.00015) | 0.821 (0.0008) |
|  | 0.3 | 0 | 0.000 (0.0015) | 0.0566 (0.00014) | 0.798 (0.0009) | -0.000 (0.0016) | 0.0564 (0.00014) | 0.799 (0.0009) |
|  |  | 10 | -0.002 (0.0016) | 0.0569 (0.00014) | 0.801 (0.0009) | -0.001 (0.0016) | 0.0565 (0.00014) | 0.799 (0.0009) |
|  |  | 20 | -0.002 (0.0016) | 0.0574 (0.00014) | 0.804 (0.0008) | -0.000 (0.0016) | 0.0566 (0.00014) | 0.800 (0.0009) |
|  |  | 30 | -0.004 (0.0016) | 0.0579 (0.00015) | 0.808 (0.0009) | 0.000 (0.0016) | 0.0570 (0.00014) | 0.803 (0.0009) |
|  |  | 40 | -0.003 (0.0016) | 0.0580 (0.00014) | 0.808 (0.0008) | -0.000 (0.0016) | 0.0575 (0.00014) | 0.806 (0.0008) |
|  |  | 50 | -0.003 (0.0016) | 0.0584 (0.00014) | 0.811 (0.0008) | 0.000 (0.0016) | 0.0574 (0.00014) | 0.806 (0.0009) |
|  |  | 60 | -0.003 (0.0016) | 0.0589 (0.00015) | 0.814 (0.0008) | 0.000 (0.0016) | 0.0576 (0.00014) | 0.807 (0.0008) |
|  |  | 70 | -0.002 (0.0017) | 0.0591 (0.00015) | 0.815 (0.0008) | -0.000 (0.0016) | 0.0580 (0.00014) | 0.810 (0.0008) |
|  |  | 80 | -0.002 (0.0017) | 0.0596 (0.00014) | 0.818 (0.0008) | 0.000 (0.0016) | 0.0584 (0.00014) | 0.812 (0.0008) |
|  |  | 90 | -0.001 (0.0017) | 0.0598 (0.00015) | 0.819 (0.0008) | -0.000 (0.0017) | 0.0588 (0.00015) | 0.815 (0.0008) |
|  | 0.5 | 0 | -0.001 (0.0013) | 0.0494 (0.00011) | 0.735 (0.0011) | -0.001 (0.0013) | 0.0495 (0.00011) | 0.737 (0.0011) |
|  |  | 10 | -0.008 (0.0014) | 0.0504 (0.00011) | 0.747 (0.0011) | -0.000 (0.0013) | 0.0500 (0.00011) | 0.743 (0.0010) |
|  |  | 20 | -0.012 (0.0014) | 0.0518 (0.00011) | 0.761 (0.0010) | -0.001 (0.0014) | 0.0508 (0.00012) | 0.750 (0.0010) |
|  |  | 30 | -0.015 (0.0014) | 0.0526 (0.00013) | 0.767 (0.0010) | -0.001 (0.0014) | 0.0516 (0.00012) | 0.758 (0.0010) |
|  |  | 40 | -0.017 (0.0015) | 0.0536 (0.00013) | 0.776 (0.0010) | -0.001 (0.0014) | 0.0524 (0.00012) | 0.765 (0.0010) |
|  |  | 50 | -0.018 (0.0015) | 0.0546 (0.00013) | 0.784 (0.0009) | -0.002 (0.0015) | 0.0530 (0.00013) | 0.771 (0.0010) |
|  |  | 60 | -0.018 (0.0015) | 0.0557 (0.00013) | 0.792 (0.0009) | -0.001 (0.0015) | 0.0540 (0.00013) | 0.779 (0.0009) |
|  |  | 70 | -0.016 (0.0016) | 0.0568 (0.00014) | 0.800 (0.0009) | -0.002 (0.0015) | 0.0549 (0.00013) | 0.786 (0.0009) |
|  |  | 80 | -0.013 (0.0016) | 0.0580 (0.00015) | 0.809 (0.0008) | -0.002 (0.0015) | 0.0558 (0.00014) | 0.793 (0.0009) |
|  |  | 90 | -0.008 (0.0017) | 0.0591 (0.00014) | 0.816 (0.0008) | -0.002 (0.0016) | 0.0573 (0.00014) | 0.804 (0.0009) |
|  | 0.7 | 0 | -0.000 (0.0008) | 0.0366 (0.00006) | 0.520 (0.0014) | -0.000 (0.0008) | 0.0364 (0.00006) | 0.515 (0.0014) |
|  |  | 10 | -0.021 (0.0009) | 0.0376 (0.00007) | 0.552 (0.0015) | -0.000 (0.0008) | 0.0374 (0.00007) | 0.542 (0.0014) |
|  |  | 20 | -0.037 (0.0009) | 0.0388 (0.00007) | 0.582 (0.0014) | -0.000 (0.0009) | 0.0384 (0.00007) | 0.565 (0.0013) |
|  |  | 30 | -0.049 (0.0010) | 0.0403 (0.00008) | 0.612 (0.0014) | -0.001 (0.0009) | 0.0397 (0.00008) | 0.592 (0.0014) |
|  |  | 40 | -0.059 (0.0010) | 0.0420 (0.00009) | 0.640 (0.0013) | -0.002 (0.0010) | 0.0410 (0.00008) | 0.619 (0.0013) |
|  |  | 50 | -0.066 (0.0011) | 0.0440 (0.00009) | 0.671 (0.0013) | -0.003 (0.0011) | 0.0427 (0.00009) | 0.649 (0.0012) |
|  |  | 60 | -0.071 (0.0012) | 0.0462 (0.00010) | 0.702 (0.0011) | -0.005 (0.0012) | 0.0446 (0.00009) | 0.678 (0.0013) |
|  |  | 70 | -0.071 (0.0013) | 0.0490 (0.00012) | 0.733 (0.0011) | -0.006 (0.0012) | 0.0470 (0.00011) | 0.709 (0.0013) |
|  |  | 80 | -0.062 (0.0014) | 0.0527 (0.00013) | 0.768 (0.0010) | -0.008 (0.0014) | 0.0500 (0.00013) | 0.742 (0.0012) |
|  |  | 90 | -0.040 (0.0016) | 0.0570 (0.00014) | 0.802 (0.0009) | -0.009 (0.0015) | 0.0533 (0.00015) | 0.772 (0.0012) |


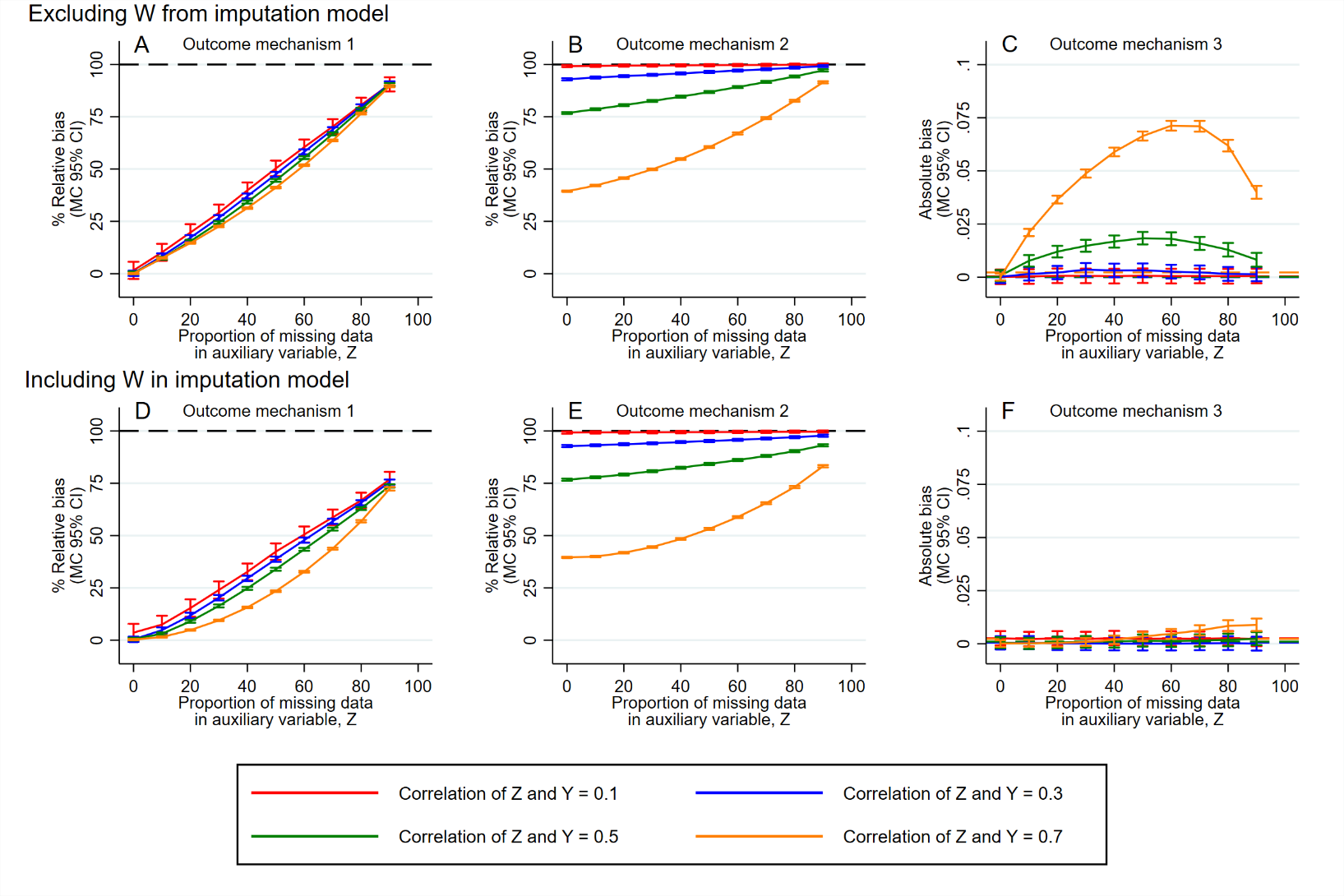


#### **Figure S7**: Plots of bias (relative to CCA for outcome mechanisms 1 and 2, and absolute for outcome mechanism 3) for the exposure (X) coefficient against the proportion of missing data in Z for sensitivity analysis that excluded W from the imputation model under missing auxiliary mechanism 3 (plots A-C).

For ease of comparison, we show the results of models in which W was included in the imputation model for missing auxiliary mechanism 3 (plots D-F; these are also shown as plots C,F and I in Figure 2 of the main text).


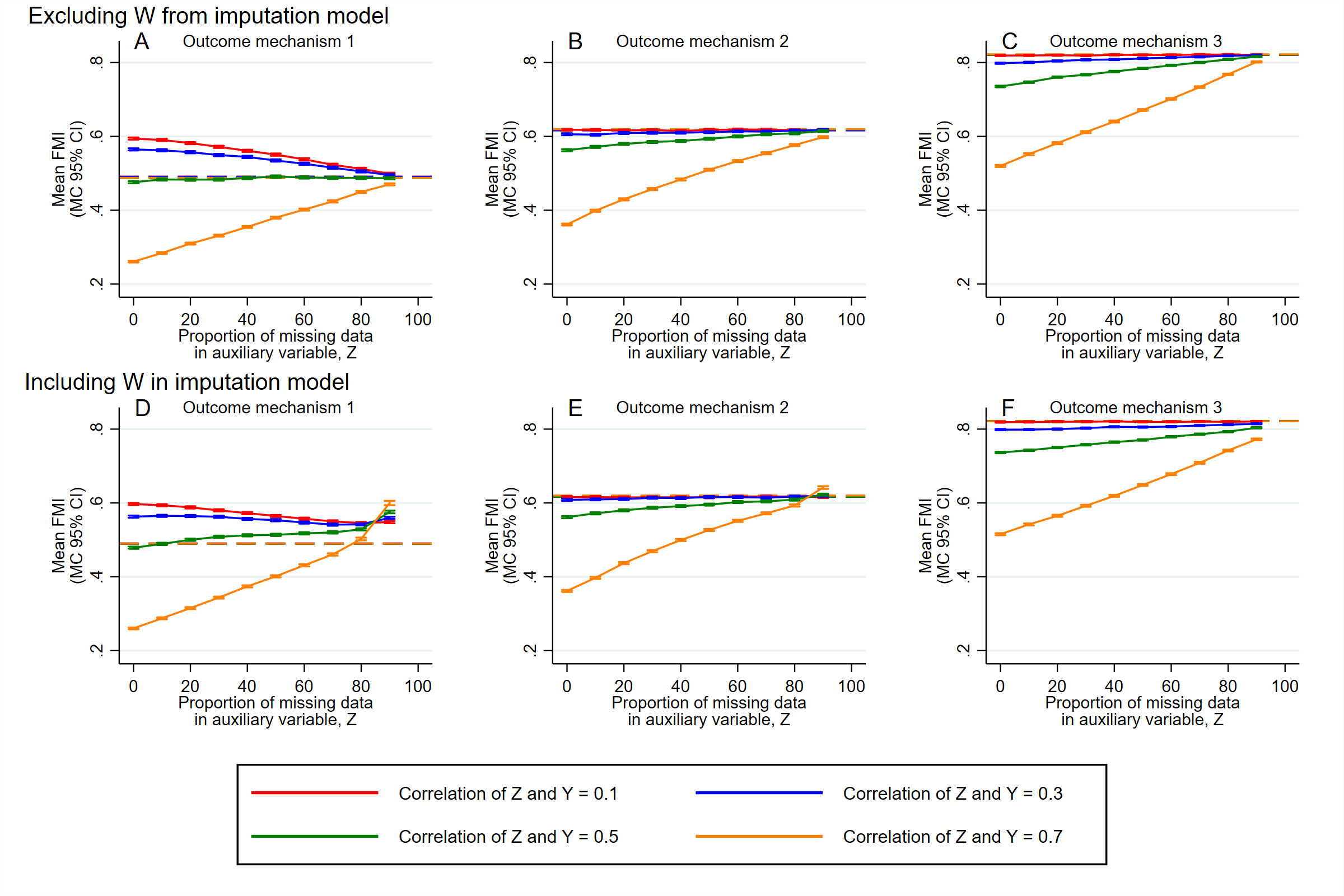


#### **Figure S8**: Plots of the FMI for the exposure (X) coefficient against the proportion of missing data in Z for sensitivity analysis that excluded W from the imputation model under missing auxiliary mechanism 3 (plots A-C).

For ease of comparison, we show the results of models in which W was included in the imputation model for missing auxiliary mechanism 3 (plots D-F; these are also shown as plots C,F and I in Figure S4).

# **Appendix S4 - Individual plots for simulation study Figures 2, S3 and S4 by outcome missingness mechanism**

## Bias – Figure 2


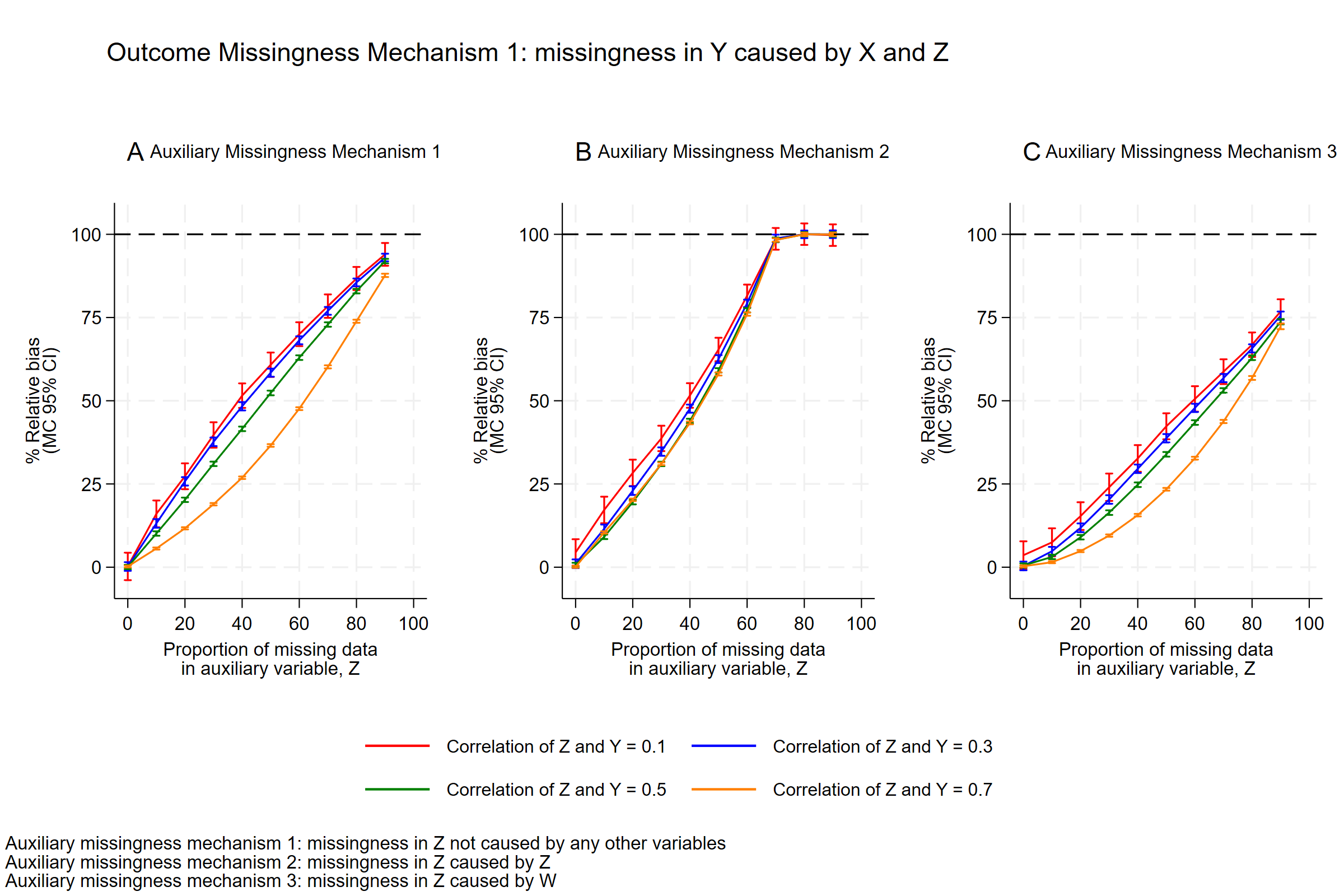


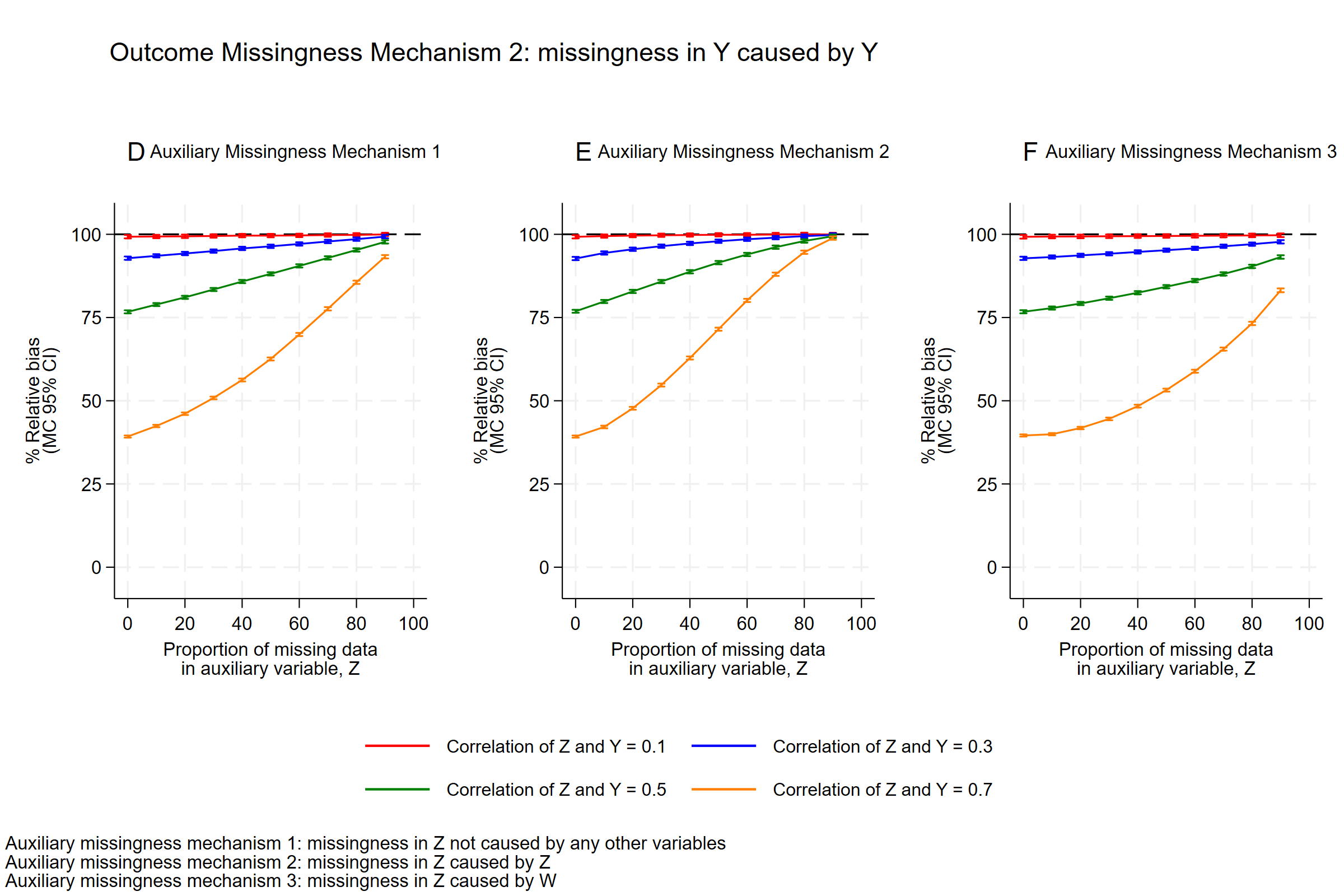


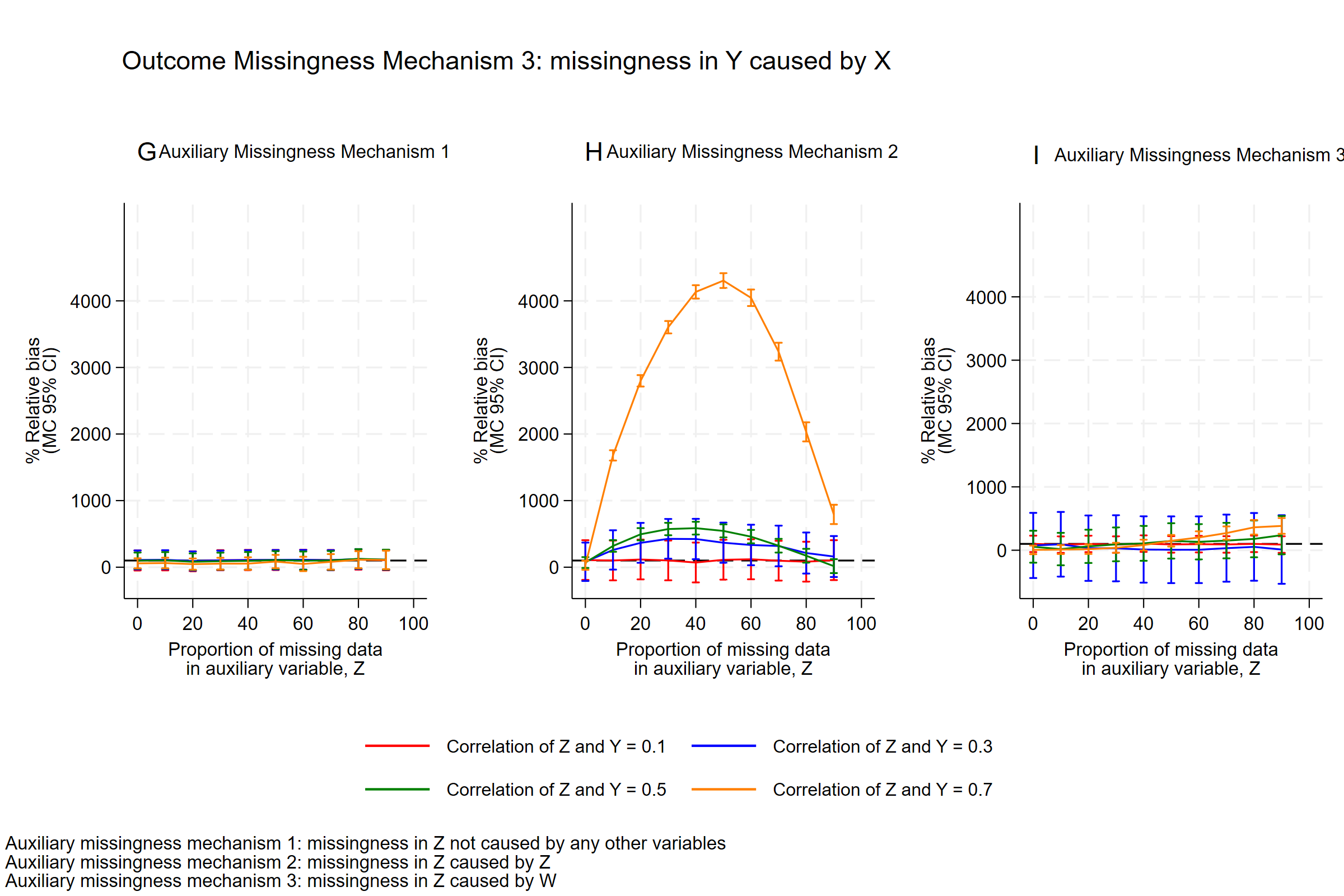


## SE – Figure S3


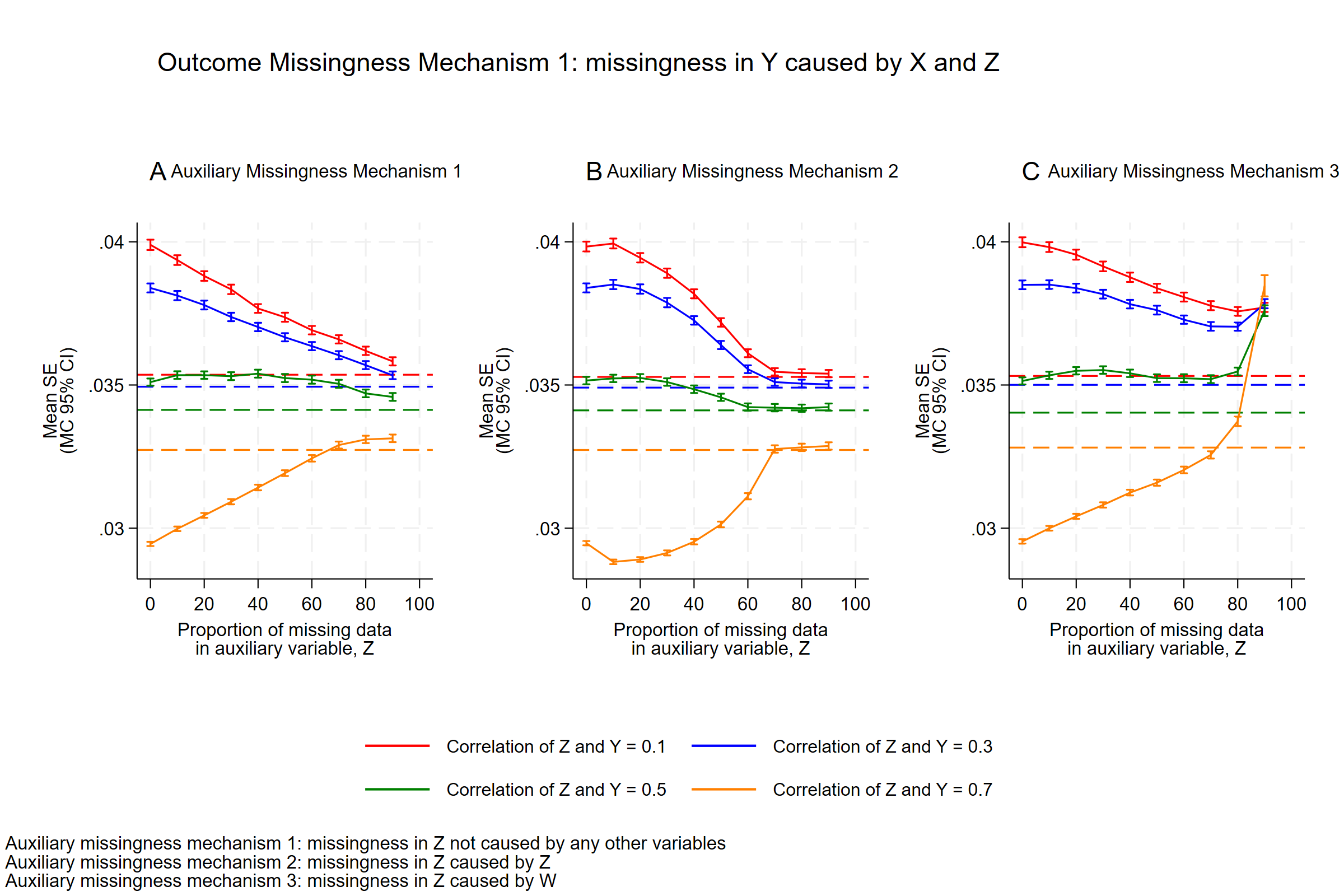


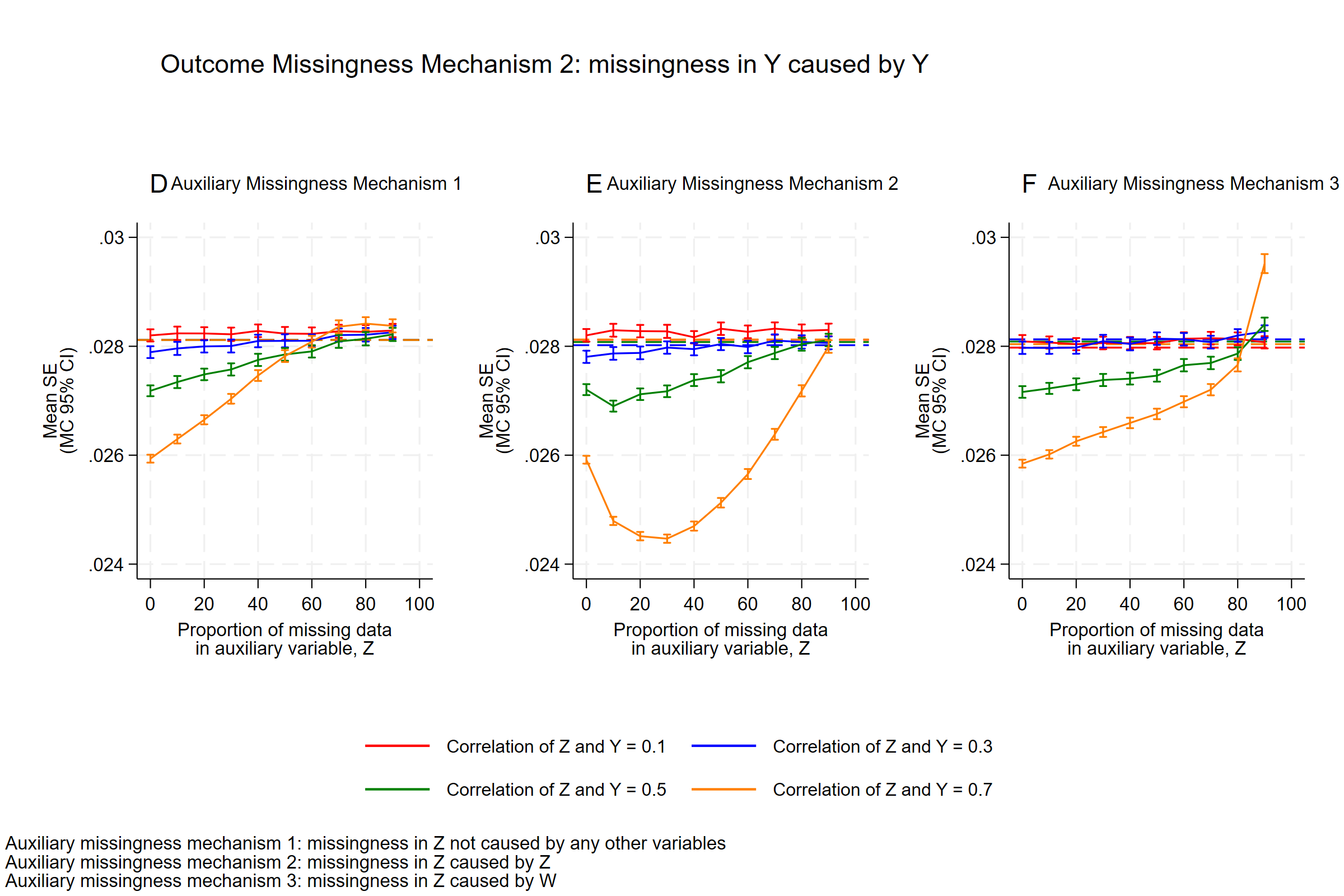


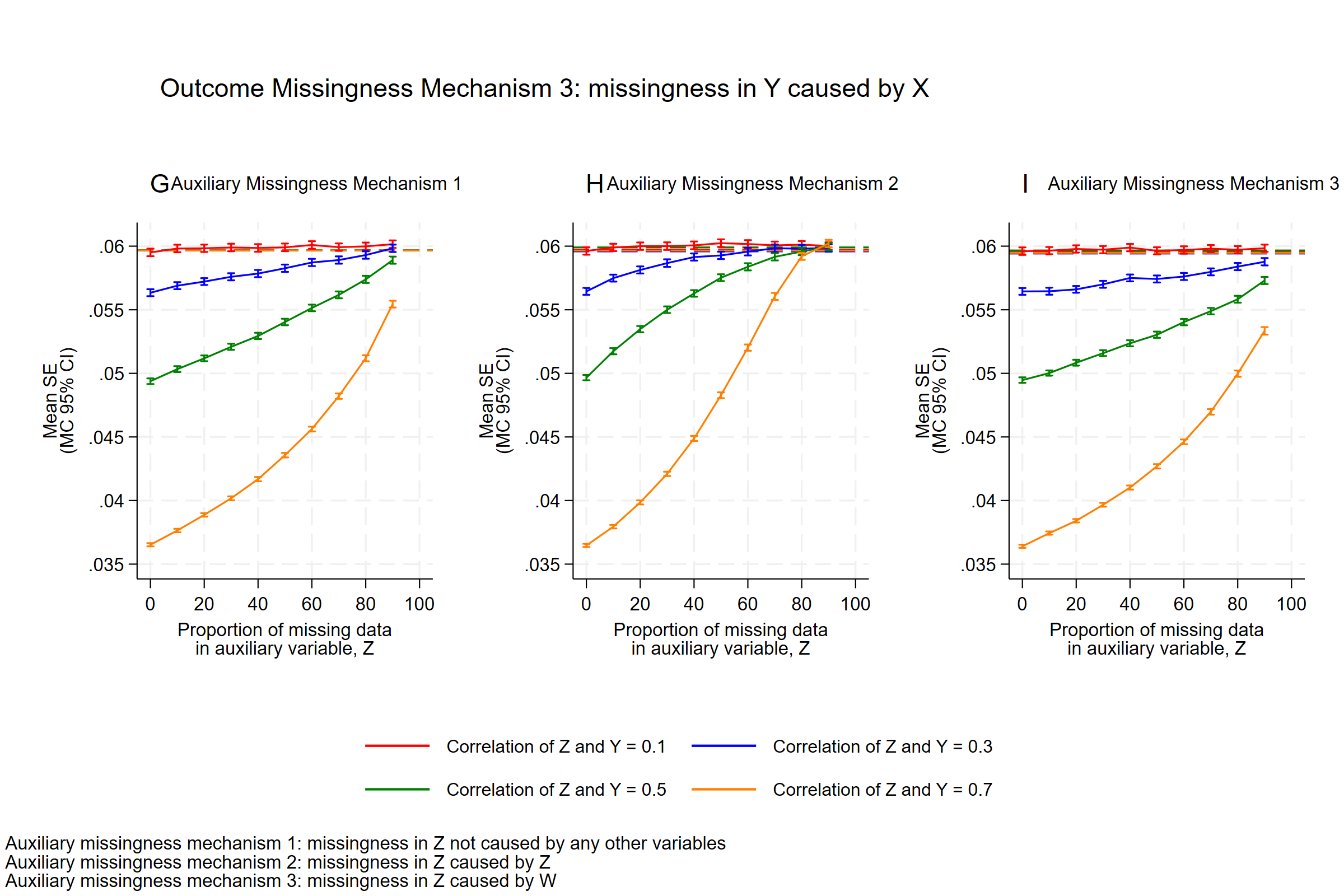


## FMI – Figure S4


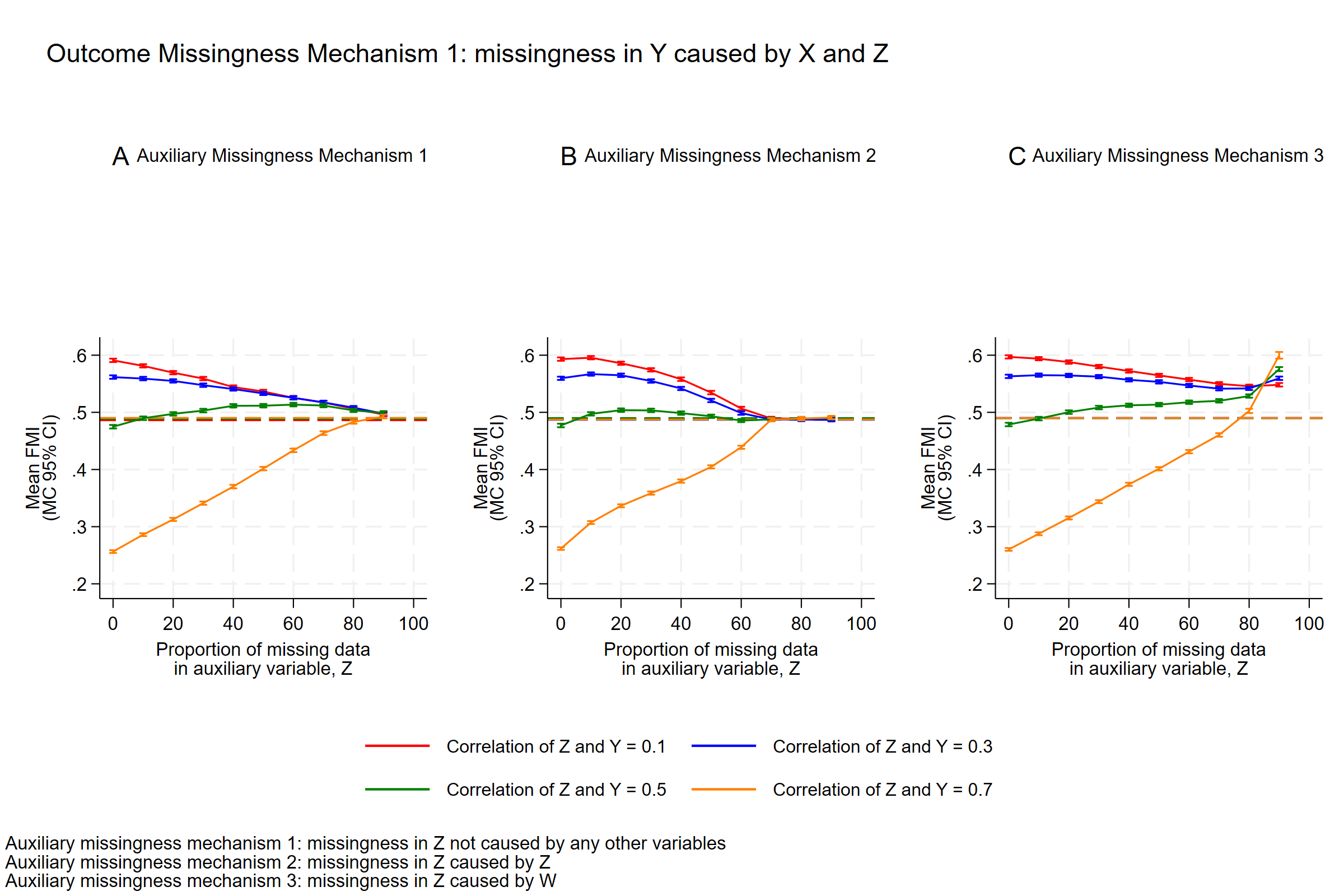


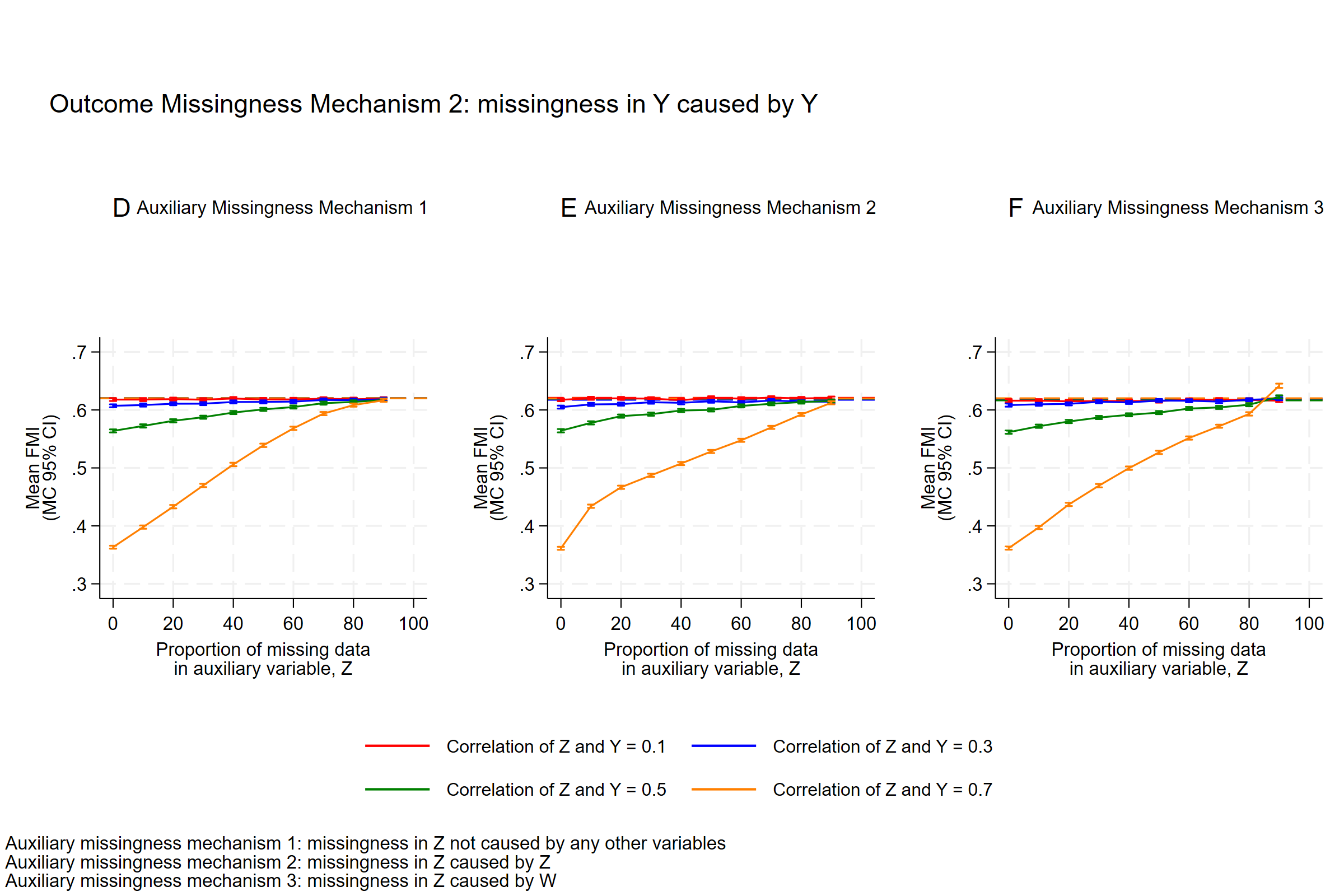


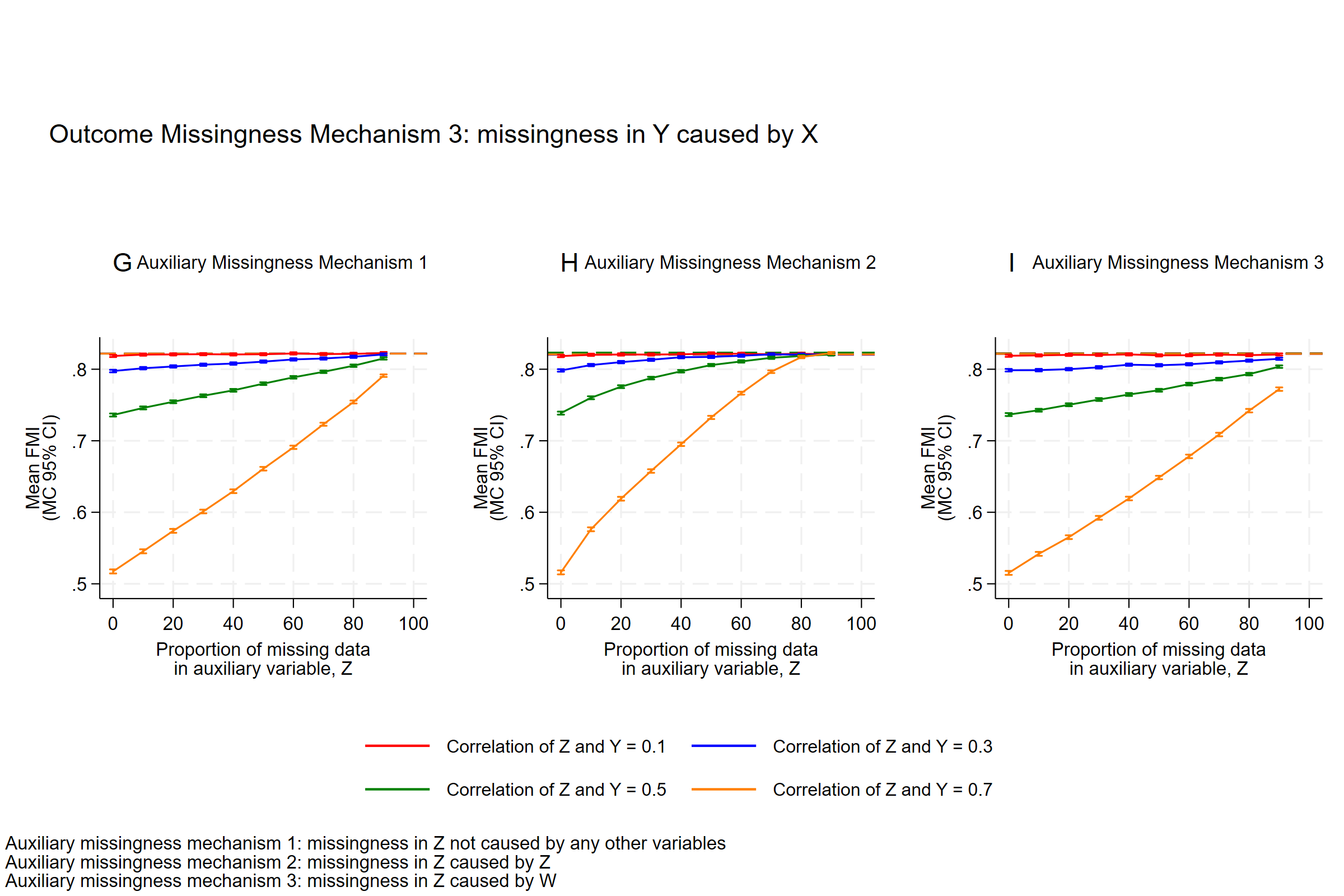


# References

1. Keogh RH, Morris TP. Multiple imputation in Cox regression when there are time-varying effects of covariates. *Stat Med*. Nov 10 2018;37(25):3661-3678. doi:10.1002/sim.7842

2. Hiscock R, Bauld L, Amos A, Fidler JA, Munafo M. Socioeconomic status and smoking: a review. *Ann N Y Acad Sci*. Feb 2012;1248:107-23. doi:10.1111/j.1749-6632.2011.06202.x

3. Lu Y, Tong S, Oldenburg B. Determinants of smoking and cessation during and after pregnancy. *Health Promotional International*. Dec 2001;16(4):355-65. doi:10.1093/heapro/16.4.355

4. Hair NL, Hanson JL, Wolfe BL, Pollak SD. Association of Child Poverty, Brain Development, and Academic Achievement. *JAMA Pediatrics*. Sep 2015;169(9):822-9. doi:10.1001/jamapediatrics.2015.1475

5. Eriksen HL, Kesmodel US, Underbjerg M, Kilburn TR, Bertrand J, Mortensen EL. Predictors of intelligence at the age of 5: family, pregnancy and birth characteristics, postnatal influences, and postnatal growth. *PLoS One*. 2013;8(11):e79200. doi:10.1371/journal.pone.0079200

6. Hackman DA, Gallop R, Evans GW, Farah MJ. Socioeconomic status and executive function: developmental trajectories and mediation. *Dev Sci*. Sep 2015;18(5):686-702. doi:10.1111/desc.12246

7. Lynn R, Kanazawa S. A longitudinal study of sex differences in intelligence at ages 7, 11 and 16 years. *Pers Indiv Differ*. 2011/08/01/ 2011;51(3):321-324. doi:<https://doi.org/10.1016/j.paid.2011.02.028>

8. Cornish RP, Macleod J, Carpenter JR, Tilling K. Multiple imputation using linked proxy outcome data resulted in important bias reduction and efficiency gains: a simulation study. *Emerg Themes Epidemiol*. 2017;14:14. doi:10.1186/s12982-017-0068-0

9. Cornish RP, Tilling K, Boyd A, Davies A, Macleod J. Using linked educational attainment data to reduce bias due to missing outcome data in estimates of the association between the duration of breastfeeding and IQ at 15 years. *Int J Epidemiol*. Jun 2015;44(3):937-45. doi:10.1093/ije/dyv035

10. Boyd A, Golding J, Macleod J, et al. Cohort Profile: the 'children of the 90s'--the index offspring of the Avon Longitudinal Study of Parents and Children. *International journal of epidemiology*. 2013;42(1):111-27.

11. Department for Education. Permanent and fixed-period exclusions in England: 2008 to 2009. 31 August, 2023. Accessed 31 August, 2023. <https://www.gov.uk/government/statistics/permanent-and-fixed-period-exclusions-in-england-academic-year-2008-to-2009>
